# C–H functionalization of amines with aryl halides by nickel-photoredox catalysis†
†Electronic supplementary information (ESI) available: Experimental details and characterization data. See DOI: 10.1039/c6sc02815b



**DOI:** 10.1039/c6sc02815b

**Published:** 2016-07-28

**Authors:** Derek T. Ahneman, Abigail G. Doyle

**Affiliations:** a Department of Chemistry , Princeton University , Princeton , NJ 08544 , USA . Email: agdoyle@princeton.edu

## Abstract

We describe the functionalization of α-amino C–H bonds with aryl halides using a combination of nickel and photoredox catalysis. This direct C–H, C–X coupling uses inexpensive and readily available starting materials to generate benzylic amines, an important class of bioactive molecules. Mechanistically, this method features the direct arylation of α-amino radicals mediated by a nickel catalyst. This reactivity is demonstrated for a range of aryl halides and *N*-aryl amines, with orthogonal scope to existing C–H activation and photoredox methodologies. We also report reactions with several complex aryl halides, demonstrating the potential utility of this approach in late-stage functionalization.

## Abstract 2

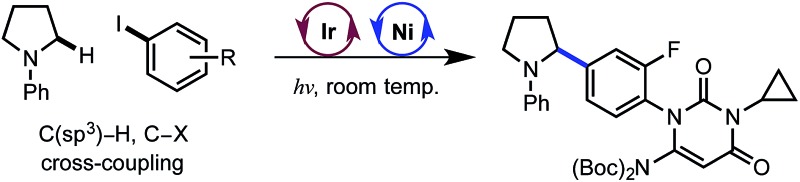
A redox-neutral method for α-amino C(sp^3^)–H arylation is described using nickel and photoredox catalysis.

## Introduction

The direct functionalization of C(sp^3^)–H bonds constitutes a powerful method for the rapid elaboration of simple organic substrates.^1^ A critical goal is to identify selective methods for converting common C–H bonds to useful functionality. Within this domain, transition metal catalysis has emerged as a prolific strategy, primarily due to its modularity and selectivity.^2^ Herein, we report the selective functionalization of α-amino C(sp^3^)–H bonds with aryl halides using a nickel-photoredox dual catalyst system. This method delivers benzylic amines, a well-represented motif among bioactive natural products and pharmaceutical compounds.^3^

**Scheme 1:**
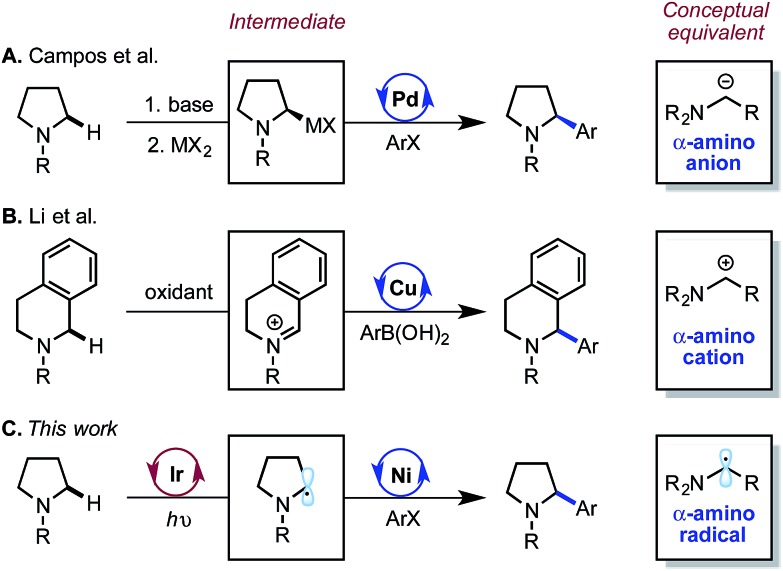
Strategies for α-amino C–H arylation where transition metals engage distinct intermediates.

Due to the importance and prevalence of saturated amines in organic synthesis, various mechanistically divergent strategies have been reported for the arylation of α-amino C(sp^3^)–H bonds by transition metal catalysis.^4^ Seminal work from Sames and co-workers first established the direct C(sp^3^)–H arylation of pyrrolidines catalyzed by Ru(0).^5^ More recently, the Yu group has reported a Pd(ii)-catalyzed α-C(sp^3^)–H arylation of thioamides using aryl boronic acids.^6^ While enabling, these methods typically require elevated temperature and a metal-chelating directing group, and have not proven amenable to asymmetric catalysis.^7^ In an effort to address these limitations, researchers have investigated approaches in which the C(sp^3^)–H activation and the metal-catalyzed functionalization mechanisms are decoupled (Scheme 1). Campos and co-workers at Merck pioneered a procedure for the enantioselective arylation of *N*-Boc-pyrrolidine *via* an asymmetric lithiation/Pd-catalyzed Negishi coupling.^8^ Additionally, the Li group described the Cu-catalyzed arylation of benzylic iminium ions derived from *in situ* oxidation of saturated amines.^9^ Conceptually, these two approaches utilize α-anion and α-cation intermediates in cross coupling. Our laboratory sought to explore a third possibility in which α-amino radicals are engaged by a metal catalyst to achieve formal C(sp^3^)–H arylation. We expected that the ability to generate α-amino radicals at room temperature from simple amines would expand the scope and enhance the functional group tolerance within this important class of reactions.^10^ Furthermore, the catalytic generation of these intermediates would obviate the need for stoichiometric activating reagents, as necessary for the α-anion and α-cation approaches.

The capture of organic radicals by transition metal catalysts remains an underexplored strategy in cross coupling.^11^ Photoredox catalysis was selected for radical generation due to its complementarities with transition metal catalysis. For example, photoredox catalysts are capable of activating organic molecules at nontraditional sites (such as C–H and C–CO_2_H) and are proficient in coupling saturated systems.^12^ Unfortunately, the coupling partners have largely been limited to radicophiles or persistent radicals and achieving asymmetric catalysis has been challenging. The MacMillan group has reported the photoredox-catalyzed α-C(sp^3^)–H arylation of *N*-aryl amines with cyanoarenes or heteroaryl halides.^13^ This work highlights the ability of photoredox catalysis to generate α-amino radicals from C–H bonds under incredibly mild conditions. However, a limitation is the requirement for coupling partners derived only from electron-deficient (hetero)arenes. We set out to demonstrate that combining the unique reactivity profile of photoredox catalyzed α-amino radical formation with the modularity conferred by a nickel catalyst in cross coupling would provide a useful complement to this system by significantly expanding the scope of possible coupling partners and offering opportunities for catalyst-controlled stereoinduction.^14,15^

## Results and discussion

**Fig. 1:**
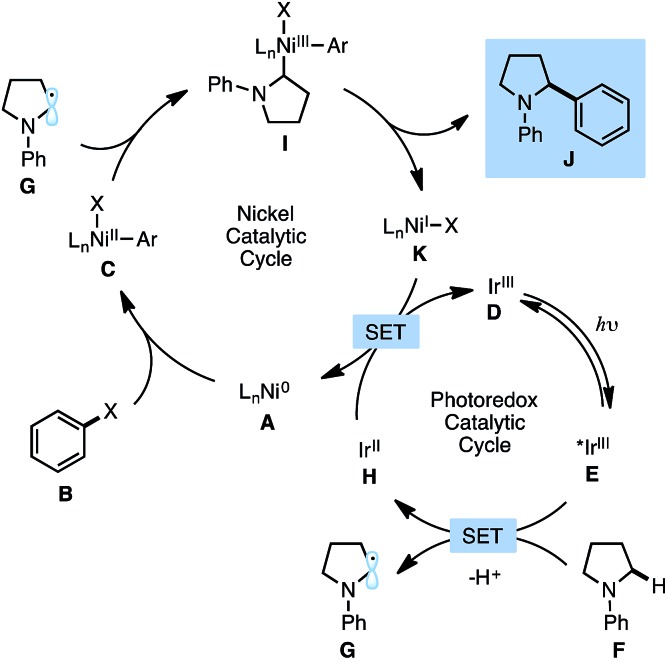
Proposed mechanism for α-amino C–H functionalization with aryl halides.

Fig. 1 describes the proposed mechanism for the dual catalytic protocol. The Ni(ii) precatalyst is first reduced to Ni(0) by the photocatalyst, presumably using the *N*-aryl amine as a sacrificial reductant. Ni(0) catalyst **A** is then capable of performing oxidative addition with aryl halide **B** to generate Ni(ii) aryl halide complex **C**. Concurrently, [Ir(dF-CF_3_-ppy)_2_(dtbbpy)]PF_6_ **D** is excited by blue light to produce excited state **E** (dF-CF_3_-ppy = 2-(2,4-difluorophenyl)-5-(trifluoromethyl)pyridine, dtbbpy = 4,4′-di-*tert*-butyl-2,2′-bipyridine). The photoexcited Ir(iii) catalyst **E** is sufficiently oxidizing (*E*red1/2[*Ir^III^/Ir^II^] = +1.21 V *vs.* SCE in MeCN)^16^ to react with *N*-phenylpyrrolidine **F** (*E*red1/2 = +0.70 V *vs.* SCE in MeCN)^17^ to generate a radical cation, which upon deprotonation delivers α-amino radical **G**. The α-amino radical is capable of intercepting Ni(ii) aryl halide **C** to generate Ni(iii) intermediate **I**, which undergoes reductive elimination to furnish product **J** and Ni(i) species **K**. Finally, the reduced form of the photocatalyst **H** (*E*red1/2[Ir^III^/Ir^II^] = –1.37 V *vs.* SCE in MeCN)^16^ reduces Ni(i) species **K** (*E*red1/2[Ni^II^/Ni^0^] = –1.2 V *vs.* SCE in DMF)^18^ by a single electron transfer event to regenerate both catalysts simultaneously.^19^
1
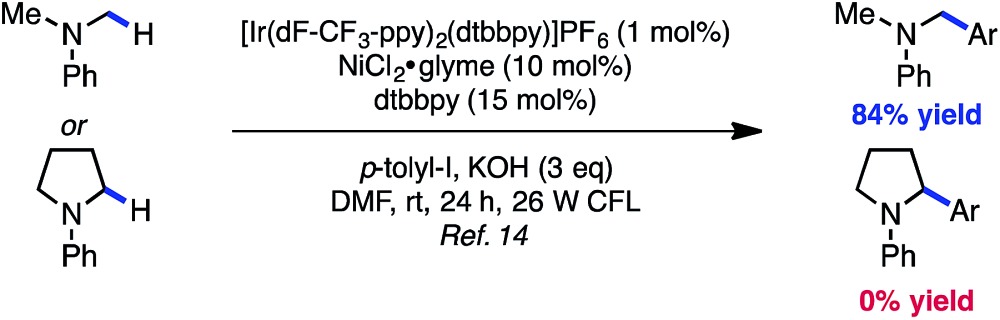



Preliminary findings from our laboratory in collaboration with the MacMillan laboratory using dimethylaniline as a model system revealed the feasibility of this approach.^14,20^ Unfortunately, substrates containing β-hydrogens were not competent under our initially reported conditions (eqn (1)). We therefore sought to identify conditions that would enable coupling of a wider range of amine coupling partners. Our investigation began with the coupling of *N*-phenylpyrrolidine with 4-iodotoluene, using NiCl_2_·glyme as catalyst. The optimization efforts first focused on identifying a ligand system for Ni capable of carrying out this reaction (Table 1, entries 1–8). Among the bi- and tridentate amine ligands evaluated, only two ligand classes, bis(pyrazolyl)pyridine (bpp) and bis(oxazoline) (BiOx), delivered measurable amounts of benzylic amine **1**. Furthermore, the steric characteristics of the BiOx ligand proved to be critical to reaction efficiency. Contrary to most literature reports,^21^ more encumbered ligands tended to generate more β-hydride elimination product *N*-phenylpyrrole. The optimized system utilizes the parent bis(oxazoline) ligand with hydrogens at the 2-position and performs α-arylation in 88% yield on 0.10 mmol scale (entry 8). Reactions employing bulkier ligands proceeded in diminished yield, with benzyl substitution delivering 54% yield and the iso-propyl variant not producing any product (entries 6 and 7). Air-stable Ni(ii) salts proved most efficient in the reaction, with NiCl_2_·glyme being optimal. Ni(0) sources such as Ni(cod)_2_ were not competent under the reported conditions (entry 9).^22^ We also explored the reaction using lower ligand loadings. On small scale, there was virtually no difference observed between 30 mol% and 20 mol% ligand (entries 8 and 10). However, on 0.40 mmol scale, a lower variance in yield was observed at higher ligand loadings. Blue LED's are superior to compact fluorescence lamps in this system due to superior overlap with the absorption spectrum of the photocatalyst (entry 11). Importantly, the amine stoichiometry can be halved with only a minor decrease in yield (entry 12). Performing the reaction under more concentrated reaction conditions resulted in erosion of reaction efficiency (entry 13). Finally, the reaction can be carried out on the benchtop, albeit in diminished yield (entry 14). As anticipated, control reactions performed without photocatalyst, Ni catalyst, or light each delivered no product. Notably, all reagents used in the optimized reaction conditions are commercially available.

**Table 1:** Reaction optimization^*a*^

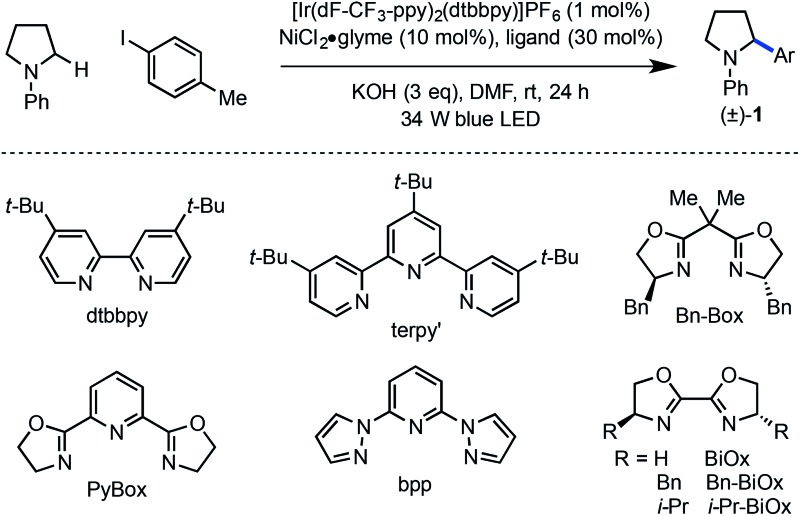			
Entry^*a*^	Ligand	Conditions^*b*^	Yield^*c*^ (%)
1	dtbbpy	As shown	0
2	terpy′	As shown	0
3	Bn-Box	As shown	0
4	PyBox	As shown	0
5	bpp	As shown	10
6	Bn-BiOx	As shown	54
7	*i*-Pr-BiOx	As shown	0
**8**	**BiOx**	**As shown**	**88**
9	BiOx	Ni(cod)_2_ instead of NiCl_2_·glyme	0
10	BiOx	20% BiOx instead of 30% BiOx	87
11	BiOx	CFL instead of blue LED	12
12	BiOx	1.5 eq. amine instead of 3.0 eq.	75
13	BiOx	0.08 M instead of 0.02 M	31
14	BiOx	Set up outside of glovebox	51

^*a*^Ar = *p*-tolyl.

^*b*^1.0 equiv. aryl iodide; 3.0 equiv. amine.

^*c*^Yield determined by ^1^H NMR spectroscopy using 1,3-bis(trifluoromethyl)-5-bromobenzene as external standard.

The electrophile scope of the reaction was evaluated (Table 2).^23^ Substitution at the *meta* and *para* positions of the aryl halide is well-tolerated (**1**, **2**), however *ortho* substitution results in diminished yield (**3**). The reaction allows for diverse electronic properties of the haloarene, including both electron-deficient (**4–6**) and electron-rich (**7–9**) substrates. Notably, the high yields observed using electron-rich aryl halides demonstrate the complementarity between this approach and previously reported photoredox reactions that require electron-deficient cyanoarenes.^13^ A chlorinated iodoarene (**10**) is also competent in the reaction, highlighting the potential for further product elaboration. The coupling of pharmaceutically relevant heterocycles is also efficient, including indole (**11**) and quinoline (**12**). Furthermore, bromopyridines are tolerated in the reaction, albeit in diminished yield (**13**, **14**). Finally, a vinyl triflate delivers similar yields under these conditions, underscoring the potential to use other cross-coupling electrophiles under similar reaction conditions.

**Table 2:** Aryl halide scope^*a*^ ^,^ ^*b*^

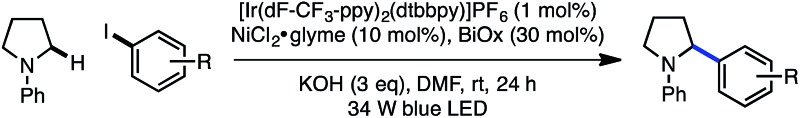
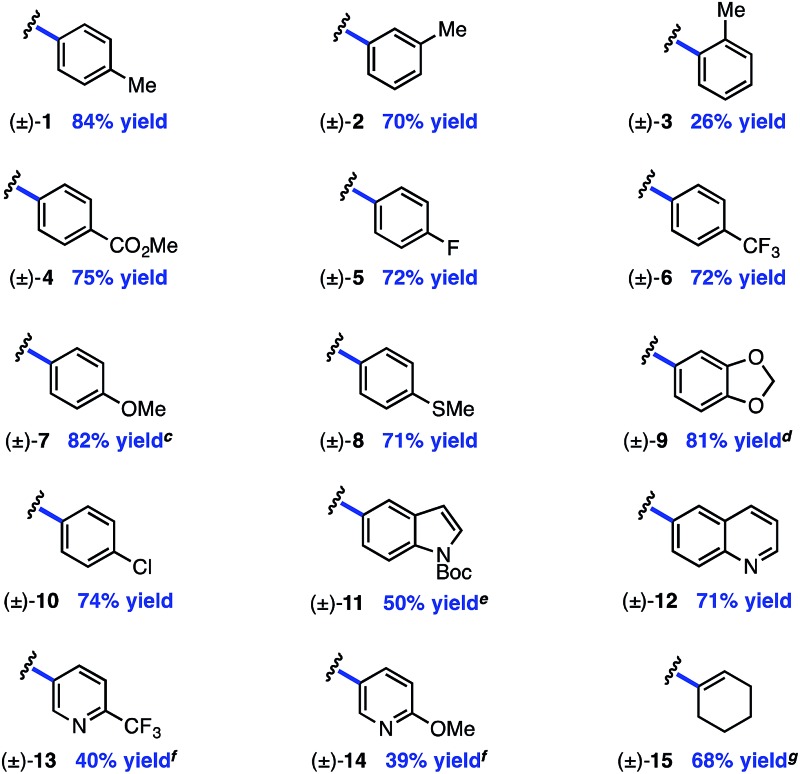

^*a*^Yield of isolated product is the average of two runs (0.40 mmol).

^*b*^1.0 equiv. aryl halide; 3.0 equiv. amine.

^*c*^Contains 5% hydrodehalogenation product.

^*d*^Contains 6% hydrodehalogenation product.

^*e*^Contains 10% inseparable impurity.

^*f*^Bromopyridine used as starting material.

^*g*^Vinyl triflate used as starting material.

Next, the amine scope of the cross-coupling reaction was investigated (Table 3). An *N*-aryl amine is currently required for the photoredox voltage-gated mechanism of C–H activation; however, using a 2-pyridyl group in place of simple phenyl also affords product in good efficiency (**16**). Acyclic *N*-methyl-*N*-alkylanilines deliver good yields in the reaction (**17**, **18**) and in the case of different alkyl groups, arylation takes place at the methyl carbon with excellent regioselectivity.^24^ A key factor for *N*-aryl amine success in the reaction appears to be radical nucleophilicity, with greater stereoelectronic overlap between the radical and amine lone pair providing enhanced efficiency. For instance, the yield is diminished for piperidine (**21**) but the flatter morpholine performs well (**22**). Finally, *N*-phenylazepane is tolerated as the amine coupling partner (**23**).

**Table 3:** Amine scope^*a*^ ^,^ ^*b*^ ^,^ ^*c*^

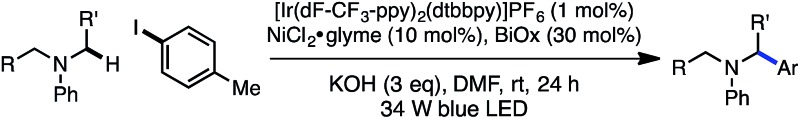
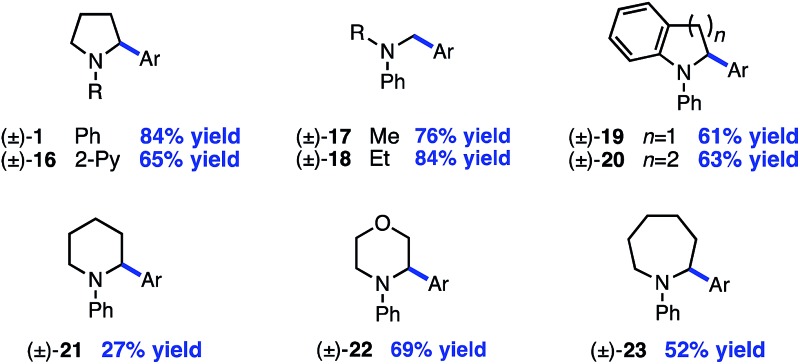

^*a*^Yield of isolated product is the average of two runs (0.40 mmol).

^*b*^1.0 equiv. aryl iodide; 3.0 equiv. amine.

^*c*^Ar = *p*-tolyl.

The reaction is compatible with a number of pharmaceutically relevant functional groups (Table 4). Notably, complete retention of existing stereocenters bearing abstractable C–H bonds is observed, and the reaction takes place selectively in the presence of weaker benzylic C–H bonds (**24**).^10^ Aldehydes and electron-rich heterocycles are also well-tolerated (**25**). Finally, substrates bearing cyclopropanes and α,β-unsaturated amides are compatible with the reported conditions (**26**). These promising results indicate that this direct C–H, C–X cross-coupling technology may find utility in the late-stage functionalization of bioactive compounds.

**Table 4:** Cross-coupling using complex aryl halides^*a*^ ^,^ ^*b*^

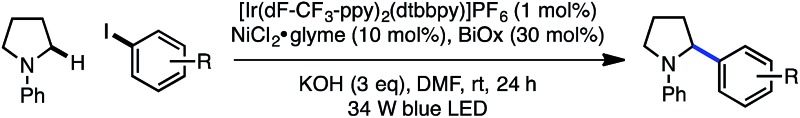
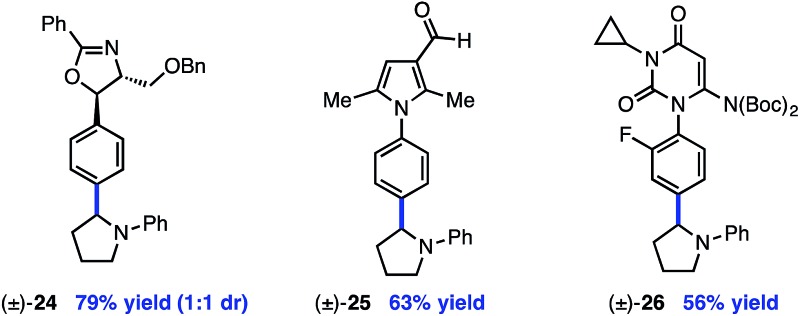

^*a*^Yield of isolated product (0.40 mmol).

^*b*^1.0 equiv. aryl iodide; 3.0 equiv. amine.

A key advantage of the metallophotoredox strategy over competing technologies is the ability to achieve catalyst-controlled selectivity under mild conditions.^15a,25^ The development of an enantioselective variant would circumvent the challenges associated with the harsh conditions of C–H activation and the difficulty of enantioinduction using a photoredox catalyst alone. Gratifyingly, the use of a chiral BiOx ligand generated product with modest enantioinduction, demonstrating the ability of the nickel catalyst to dictate facial selectivity in the arylation protocol (eqn (2)).^26^ We hope this strategy will prove enabling in the asymmetric functionalization of C–H bonds.2
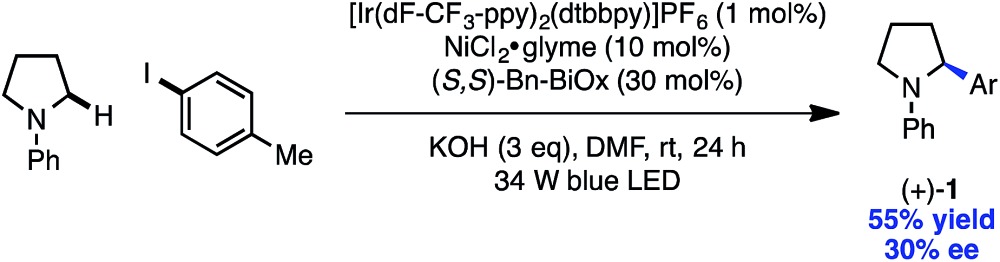



## Conclusions

In summary, we have developed a direct cross-coupling of amines with aryl halides using a nickel-photoredox dual catalyst system. This mild cross coupling protocol provides direct access to benzylic amines from inexpensive and readily available starting materials without the need for prefunctionalization. The electrophile scope is also notable, with a range of electronically diverse (hetero)aryl halides and even a vinyl triflate acting as viable coupling partners, making this technology complementary to existing photoredox methods. Finally, this chemistry is compatible with complex aryl halides, underscoring the opportunity for late-stage functionalization of bioactive molecules.
