# Supplementary material for: C–H functionalization of amines with aryl halides by nickel-photoredox catalysis
Source: Chem Sci. 2016 Jul 28;7(12):7002–6. doi: 10.1039/c6sc02815b (PMC5207500; doi:10.1039/c6sc02815b)

Electronic Supplementary Material (ESI) for Chemical Science.
This journal is © The Royal Society of Chemistry 2016

Supporting Information

Table of Contents

I. General Information S2

II. Compound Index S4

III. Reaction Optimization Studies S7

IV. Emission and Absorption Spectra S8

V. Substrate Preparation S9

VI. Product Characterization S13

VII. Enantioselective Result S27

VIII. NMR Spectra S28

I. General Information

General Procedures. Unless otherwise noted, reactions were performed under a nitrogen atmosphere with the exclusion of moisture. Reactions were monitored by thin-layer chromatography (TLC) on EMD Silica Gel 60 F_{254} plates, visualizing with UV light (254 nm). Organic solutions were concentrated under reduced pressure using a rotary evaporator (25 °C, <50 torr). Automated column chromatography was performed using pre-packed silica gel cartridges on a Biotage SP4 (40-53 µm, 60 Å). Preparative TLC was performed on SiliaPlate Prep plates (1000 µm, 20 x 20 cm). Preparative supercritical fluid chromatography (SFC) was carried out by Lotus Separations.

Materials. Commercial reagents were purchased from Sigma-Aldrich, Alfa Aesar, Strem, Oakwood, Combi-Blocks, Enamine, AstaTech, or ChemShuttle and used as received with the following exceptions. Tetrahydrofuran (THF) and toluene were dried by passing through activated alumina columns;[1] *N,N*-dimethylformamide (DMF) was dried by passing through a column of activated molecular sieves and degassed prior to use. $Ni(cod)_2$ was purchased from Strem and stored at −40 °C in a nitrogen-filled glovebox. Nickel (II) chloride dimethoxyethane (Strem) was stored at room temperature in a nitrogen-filled glovebox.[2] Potassium hydroxide (Sigma-Aldrich) was very finely ground with a mortar and pestle in the glovebox before use.

Instrumentation. Proton nuclear magnetic resonance (^{1}H NMR) spectra were recorded on a Bruker 500 MHz AVANCE spectrometer. Proton chemical shifts are reported in parts per million downfield from tetramethylsilane and are referenced to residual protium in the NMR solvent ($CHCl_3$ = δ 7.26 ppm). Carbon nuclear magnetic resonance (^{13}C NMR) were recorded on a Bruker 500 AVANCE spectrometer (125 MHz). Chemical shifts for carbon are reported in parts per million downfield from tetramethylsilane and are referenced to the carbon resonances of the solvent residual peak ($CDCl_3$ = δ 77.16 ppm). Fluorine nuclear magnetic resonance (^{19}F NMR) were reported on a Bruker 300 AVANCE (282 MHz) spectrometer. NMR data are represented as follows: chemical shift (δ ppm), multiplicity (s = singlet, d = doublet, t = triplet, q = quartet, m = multiplet), coupling constant in Hertz (Hz), integration.

High-resolution mass spectrometry was performed on an Agilent 6220 LC/MS using electrospray ionization time-of-flight (ESI-TOF). FT-IR spectra were recorded on a Perkin-Elmer

[1] A. B. Pangborn, M. A. Giardello, R. H. Grubbs, R. K. Rosen, F. J. Timmers, *Organometallics* **1996**, *15*, 1518–1520.
[2] $NiCl_2$•glyme that had been in the glovebox for extended periods of time (>6 months) gave lower yields, presumably due to loss of glyme. See: M. R. Prinsell, D. A. Everson, D. J. Weix, *Chem. Commun.* **2010**, *46*, 5743–5745, Supporting Information.

Paragon 500 and are reported in terms of frequency of absorption (cm^{-1}). Reversed-phase liquid chromatography/mass spectrometry (LC/MS) was performed on an Agilent 1260 Infinity analytical LC and Agilent 6120 Quadrupole LC/MS system using electrospray ionization/atmospheric-pressure chemical ionization (ESI/APCI) and UV detection at 254 nm and 280 nm. High-performance liquid chromatography (HPLC) was performed on an Agilent 1200 series instrument with a binary pump and a diode array detector. The optical rotation was taken with a Jasco P-1010 polarimeter Na/Hal lamp with a 0.5 dm/1 mL cell in spectral grade chloroform.

Light Sources. Screening scale reactions (0.10 mmol) and scaled-up reactions (0.40 mmol) were carried out using Blue Kessil H150 LED Grow Lights. Standard compact fluorescent lamps (26 W, 1600 lumens) gave diminished yield on screening scale.

II. Compound Index

ID	Structure	Preparation	NMR Spectra
**SI-1**	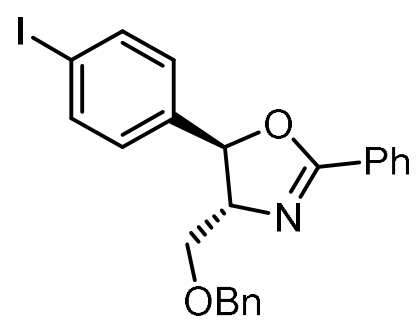	**S11**	**S28**
**SI-2**	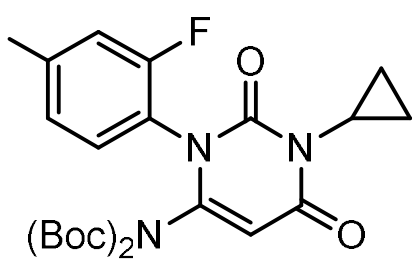	**S11**	**S30**
**1**	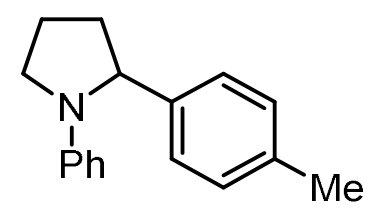	**S13**	**S35**
**2**	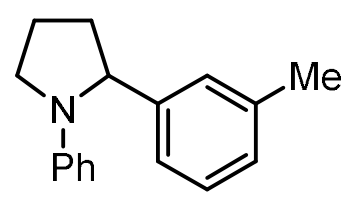	**S14**	**S37**
**3**	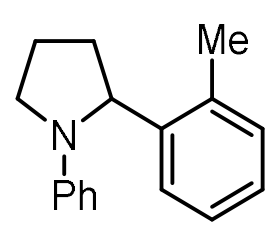	**S14**	**S39**
**4**	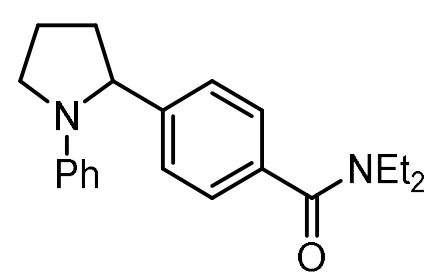	**S15**	**S41**
**5**	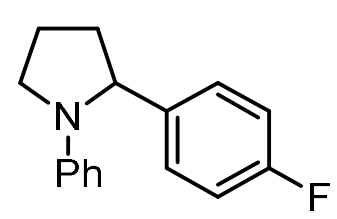	**S15**	**S43**
**6**	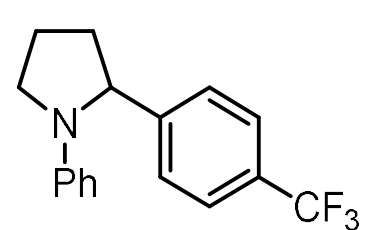	**S16**	**S46**
**7**	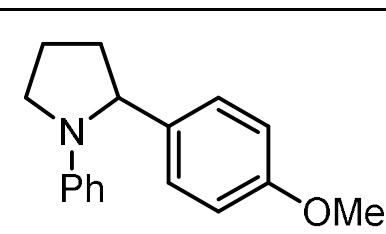	**S16**	**S49**
**8**	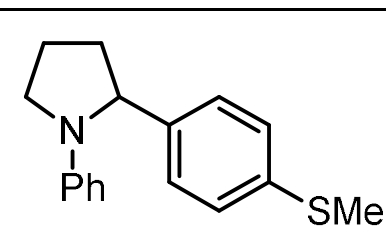	**S17**	**S51**
**9**	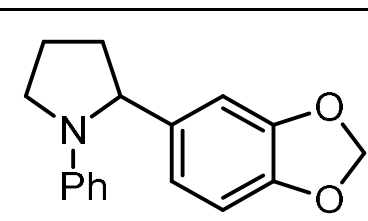	**S17**	**S53**

ID	Structure	Preparation	NMR Spectra
**10**	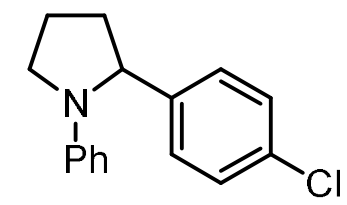	**S18**	**S55**
**11**	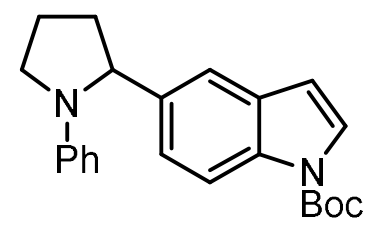	**S18**	**S57**
**12**	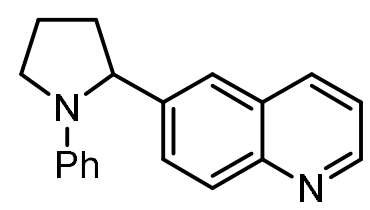	**S19**	**S59**
**13**	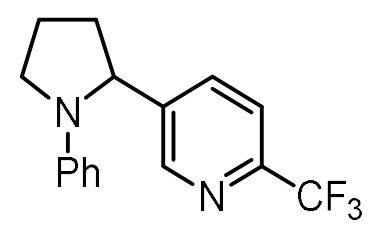	**S19**	**S61**
**14**	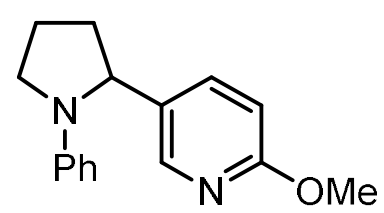	**S20**	**S64**
**15**	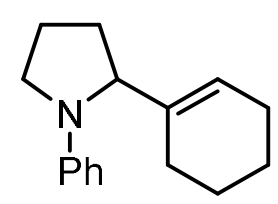	**S20**	**S66**
**16**	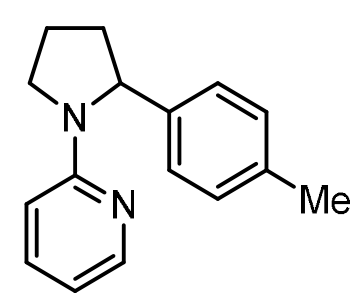	**S21**	**S68**
**17**	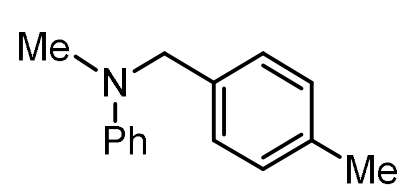	**S21**	**S70**
**18**	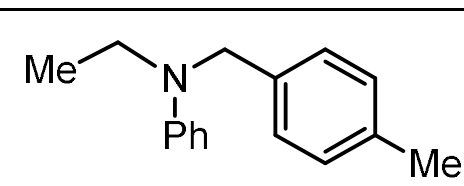	**S22**	**S72**
**19**	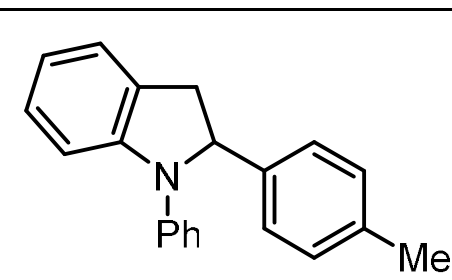	**S22**	**S74**
**20**	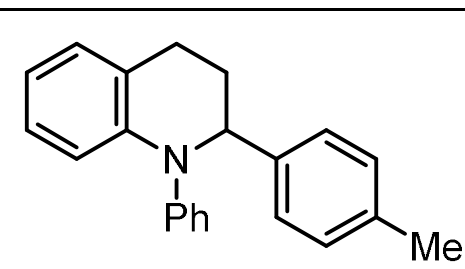	**S23**	**S76**
**21**	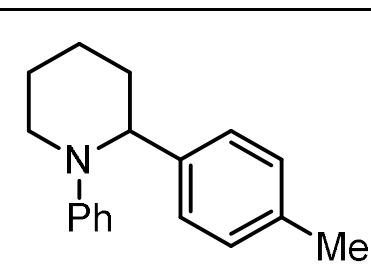	**S23**	**S78**

ID	Structure	Preparation	NMR Spectra
**22**	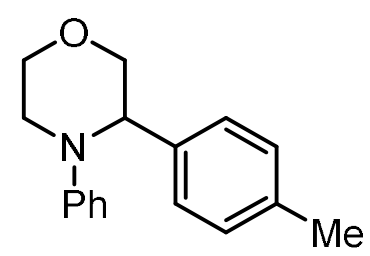	**S24**	**S80**
**23**	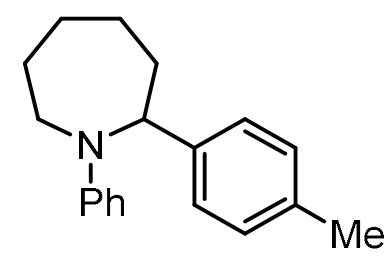	**S24**	**S82**
**24**	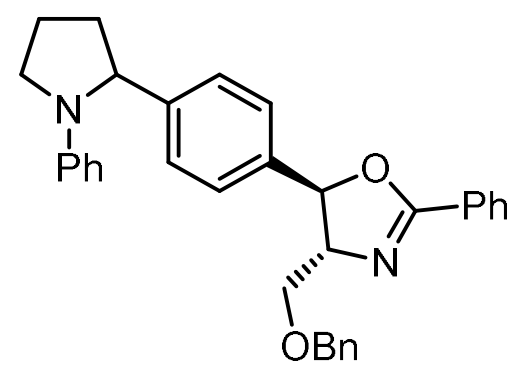	**S25**	**S84**
**25**	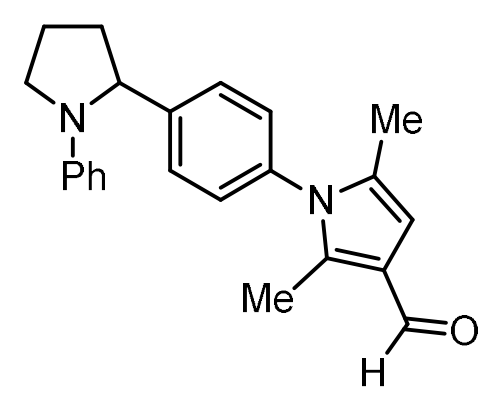	**S25**	**S86**
**26**	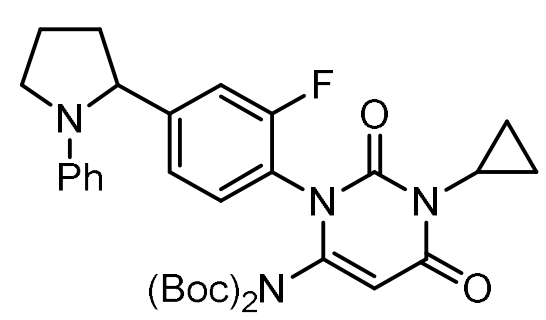	**S26**	**S88**

III. Reaction Optimization Studies

Procedure for Reaction Optimization (Table 1, Entries 1−13). A two-dram vial equipped with a PTFE-coated stir bar was transferred to a N_2-filled glovebox and charged with the appropriate ligand (0.030 mmol, 0.30 equiv). The appropriate nickel source (0.010 mmol, 0.10 equiv) in DMF (1.0 mL) was added and the solution stirred until the ligand was fully dissolved (5 min). At that point, the following prestirred solutions were added to the reaction vials: [Ir(dF-CF_3-ppy)$_2$(dtbbpy)]PF_6 (1.1 mg, 1.0 μmol, 0.010 equiv) in DMF (1.0 mL); *p*-iodotoluene (21.8 mg, 0.10 mmol, 1.0 equiv) in DMF (1.0 mL); *N*-phenylpyrrolidine (43.0 μL, 0.30 mmol, 3.0 equiv) in DMF (1.0 mL); KOH (16.8 mg, 0.30 mmol, 3.0 equiv) in DMF (1.0 mL). The reaction vial was closed with a screw cap containing a Teflon septum and sealed with electrical tape. The reaction was removed from the glovebox and irradiated with the appropriate lighting source (with cooling fan to keep the reaction at 25 °C). After 24 hours, the reaction was exposed to air and shaken. An aliquot was taken from the ether layer, diluted in acetone-d_6, and analyzed by ^{1}H NMR spectroscopy.

Procedure for Reaction Setup Outside of Glovebox (Table 1, Entry 14). A two-dram vial equipped with a PTFE-coated stir bar was charged with $NiCl_2$•glyme (2.2 mg, 0.010 mmol, 0.10 equiv) and BiOx (4.2 mg, 0.030 mmol, 0.30 equiv). Another two-dram vial equipped with a PTFE-coated stir bar was charged with [Ir(dF-CF_3-ppy)$_2$(dtbbpy)]PF_6 (1.1 mg, 1.0 μmol, 0.010 equiv), *p*-iodotoluene (21.8 mg, 0.10 mmol, 1.0 equiv), and KOH (16.8 mg, 0.30 mmol, 3.0 equiv). The two vials were put under N_2 and DMF was added to each (1.0 mL to Ni/ligand; 4.0 mL to Ir/ArX/KOH). The solutions were sparged with N_2 for 15 minutes. To the Ir/ArX/KOH solution were added the entire Ni/ligand solution (1.0 mL) and *N*-phenylpyrrolidine (43.0 μL, 0.30 mmol, 3.0 equiv). The vial was sealed with parafilm and irradiated with a Kessil lamp (with cooling fan to keep the reaction at 25 °C). After 24 hours, the reaction was exposed to air and shaken. 1,3-bis(trifluoromethyl)-5-bromobenzene (17.2 μL, 1.0 equiv) was added to the reaction as external standard. A 1 mL aliquot was taken, diluted with water (3 mL) and diethyl ether (3 mL), and shaken. An aliquot was taken from the ether layer, diluted in acetone-d_6, and analyzed by ^{1}H NMR spectroscopy.

IV. Emission and Absorption Spectra

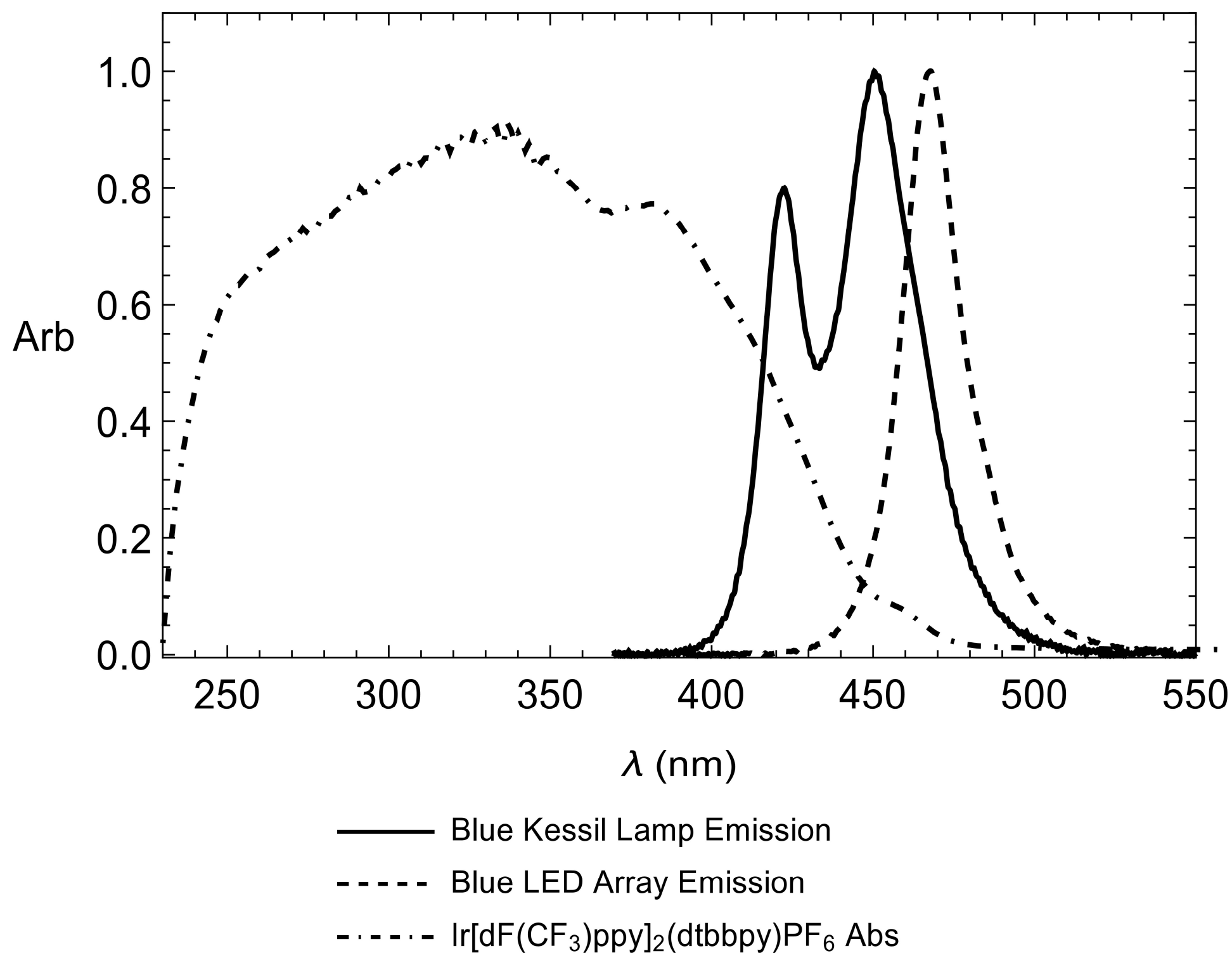

Arb = arbitrary units (spectra not to scale).

V. Substrate Preparation

Synthesis of Amine Substrates

The following amine substrates were purchased commercially from the indicated suppliers, brought into a N_2-filled glovebox, and used as received: *N*,*N*-dimethylaniline (Sigma-Aldrich), *N*-phenylpyrrolidine (Alfa Aesar), *N*-phenylpiperidine (Sigma-Aldrich), *N*-phenylmorpholine (Sigma-Aldrich). *N*-phenylindoline, [3] *N*-phenyl-1,2,3,4-tetrahydroquinoline, [4] and 1-phenylazepane[5] were prepared according to known procedures.

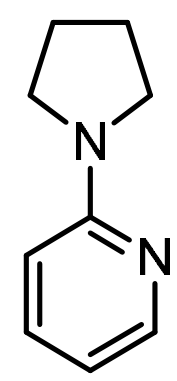

2-(pyrrolidin-1-yl)pyridine. Prepared according to a known procedure.[6] Spectroscopic data matched those previously reported.[7]

To a 25-mL conical flask fitted with a reflux condenser was added 2-bromopyridine (2.00 mL, 21.0 mmol, 1.0 equiv) and pyrrolidine (3.88 mL, 47.2 mmol, 2.25 equiv). The reaction was heated to 120 °C for 20 minutes. Upon cooling to room temperature, the reaction mixture was diluted with dichloromethane (75 mL) and washed with 1 M Na_2CO_3 (75 mL), water (75 mL), and brine (75 mL). The organic layer was dried over $MgSO_4$, filtered, and the solvent was removed *in vacuo* to afford the desired compound as a colorless oil (2.39 g, 77% yield).

^{1}H NMR (300 MHz, $CDCl_3$): δ 8.17 – 8.12 (m, 1H), 7.41 (ddd, *J* = 8.7, 7.1, 1.9 Hz, 1H), 6.53 – 6.45 (m, 1H), 6.34 (d, *J* = 8.5 Hz, 1H), 3.44 (t, *J* = 6.6 Hz, 4H), 2.04 – 1.95 (m, 4H).

^{13}C NMR (125 MHz, $CDCl_3$): δ 157.4, 148.3, 137.0, 111.1, 106.6, 46.7, 25.7.

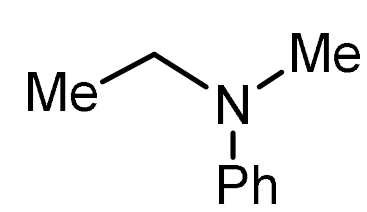

***N*-ethyl-*N*-methylaniline.** Prepared according to a known procedure.[8] Spectroscopic data matched those previously reported.[9]

---

[3] B. L. Korbad, S.-H. Lee, *Chem. Commun.* **2014**, *50*, 8985–8988.
[4] J. Meneyrol, P. Helissey, C. Tratrat, S. Giorgi-Renault, H.-P. Husson, *Synth. Commun.* **2001**, *31*, 987–992.
[5] A. Noble, D. W. C. MacMillan, *J. Am. Chem. Soc.* **2014**, *136*, 11602–11605.
[6] S. Narayan, T. Seelhammer, R. E. Gawley, *Tet. Lett.* **2004**, *45*, 757–759.
[7] Y. Zhang, X. Yang, Q. Yao, D. Ma, *Org. Lett.* **2012**, *14*, 3056–3059.
[8] N. Sakai, K. Fujii, T. Konakahara, *Tet. Lett.* **2008**, *49*, 6873–6875.
[9] I. Sorribes, K. Junge, M. Beller, *J. Am. Chem. Soc.* **2014**, *136*, 14314–14319.

To a 100-mL conical flask fitted with a reflux condenser were added chloroform (33 mL), *N*-methyl-*N*-phenylacetimide (5.00 g, 33.5 mmol, 1.0 equiv), $InBr_3$ (594 mg, 1.68 mmol, 0.050 equiv), and triethylsilane (21.4 mL, 134 mmol, 4.0 equiv). The reaction was heated to 60 °C for 16 hours, during which period the solution turned from colorless to yellow and then to orange. After cooling to room temperature, 1 M HCl (200 mL) was added to the mixture, which was then basified with 1 M NaOH and extracted with dichloromethane (3 × 200 mL). The combined organic layer was dried over $MgSO_4$, filtered, and the solvent was removed *in vacuo*. Purification of the crude reaction mixture was carried out using automated chromatographic separation on silica gel using a gradient of 1% EtOAc/Hex → 10% EtOAc/Hex to afford a colorless oil, which was then distilled to deliver the desired compound as a colorless oil (3.48 g, 77% yield).

^{1}H NMR (500 MHz, $CDCl_3$): δ 7.19 – 7.12 (m, 2H), 6.64 (d, *J* = 8.2 Hz, 2H), 6.61 (t, *J* = 7.2 Hz, 1H), 3.32 (q, *J* = 7.1 Hz, 2H), 2.82 (s, 3H), 1.04 (t, *J* = 7.1 Hz, 3H).

^{13}C NMR (125 MHz, $CDCl_3$): δ 149.2, 129.3, 116.1, 112.5, 46.9, 37.6, 11.3.

Synthesis of Aryl Halide Substrates

The following aryl iodides were purchased commercially from the indicated suppliers and used as received: *p*-iodotoluene (Sigma-Aldrich), *m*-iodotoluene (Sigma-Aldrich), *o*-iodotoluene (Sigma-Aldrich), *N*,*N*-diethyl-4-iodobenzamide (Combi-Blocks), *p*-fluoroiodobenzene (Sigma-Aldrich), 1-iodo-4-(trifluoromethyl)benzene (Sigma-Aldrich), 1-iodo-4-methoxybenzene (Sigma-Aldrich), 1-iodo-4-(methylsulfanyl)benzene (Oakwood), 1-iodo-3,4-methylenedioxybenzene (Oakwood), *p*-chloroiodobenzene (Sigma-Aldrich), *tert*-butyl 5-iodo-1*H*-indole-1-carboxylate (Combi-Blocks), 6-iodoquinoline (Combi-Blocks), 5-bromo-2-(trifluoromethyl)pyridine (Oakwood), 5-bromo-2-methoxypyridine (Oakwood), cyclohex-1-en-1-yl trifluoromethanesulfonate (Sigma-Aldrich), and 1-(4-iodophenyl)-2,5-dimethyl-1*H*-pyrrole-3-carbaldehyde (Enamine).

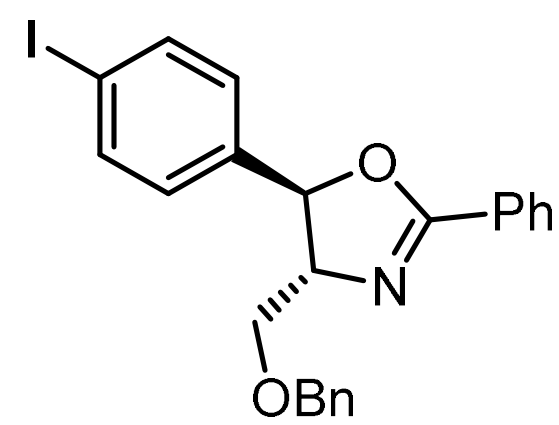

(4*R*,5*R*)-4-((benzyloxy)methyl)-5-(4-iodophenyl)-2-phenyl-4,5-dihydrooxazole (SI-1). Prepared according to a related procedure.[10]

To a scintillation vial with a teflon cap was added NaH (131 mg [60 wt%], 3.29 mmol, 2.19 equiv) and DMF (2.0 mL). ((4*R*,5*R*)-5-(4-iodophenyl)-2-phenyl-4,5-dihydrooxazol-4-yl)methanol [AstaTech] (569 mg, 1.50 mmol, 1.0 equiv) in THF (5.0 mL) was added and the reaction was stirred for 30 min, at which point benzyl bromide (272 µL, 2.29 mmol, 1.53 equiv) and tetrabutylammonium iodide (49.9 mg, 0.135 mmol, 0.090 equiv) were added. The reaction mixture was stirred for 1 h, quenched carefully with water (10 mL), and extracted with diethyl ether (4 x 10 mL). The combined organic layer was dried over $MgSO_4$, filtered, and the solvent was removed *in vacuo*. The crude reaction mixture was purified using automated chromatographic separation on silica gel using a gradient of 5% EtOAc/Hex → 50% EtOAc/Hex to afford the desired compound as a white solid (641 mg, 91% yield).

^{1}H NMR (500 MHz, $CDCl_3$): δ 7.93 (d, *J* = 7.2 Hz, 2H), 7.58 (d, *J* = 8.4 Hz, 2H), 7.41 (t, *J* = 7.4 Hz, 1H), 7.34 (t, *J* = 7.5 Hz, 2H), 7.30 – 7.17 (m, 5H), 6.98 (d, *J* = 8.3 Hz, 2H), 5.38 (d, *J* = 6.8 Hz, 1H), 4.56 – 4.47 (m, 2H), 4.22 (td, *J* = 7.0, 4.2 Hz, 1H), 3.77 (dd, *J* = 9.7, 4.2 Hz, 1H), 3.54 (dd, *J* = 9.6, 7.4 Hz, 1H).

^{13}C NMR (125 MHz, $CDCl_3$): δ 164.1, 140.8, 138.0, 137.8, 131.8, 128.5, 127.9, 127.8, 127.5, 127.4, 93.7, 83.5, 75.1, 73.5, 71.7. (Two peaks are obscured due to overlap.)

HRMS (ESI-TOF): Calculated for $C_{23}H_{21}INO_2^+$ ($[M + H]^+$): 470.0611, found 470.0607.

FTIR (thin film): 2857, 1649, 1484, 1450, 1322, 1083, 1059, 736, 692 cm^{-1}.

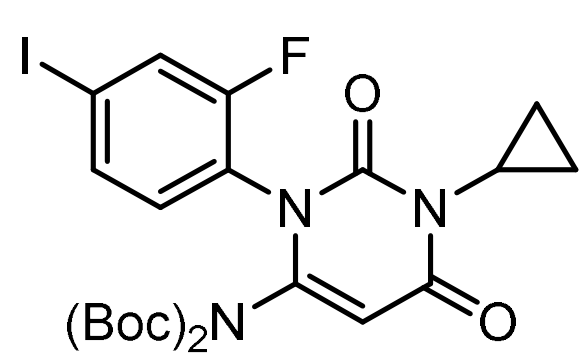

6-bis(*tert*-butoxycarbonyl)amino-3-cyclopropyl-1-(2-fluoro-4-iodophenyl)pyrimidine-2,4(1*H*,3*H*)-dione (SI-2). Prepared according to a related procedure.[11]

To a 100-mL conical flask were added 6-amino-3-cyclopropyl-1-(2-fluoro-4-iodophenyl)pyrimidine-2,4(1*H*,3*H*)-dione [ChemShuttle] (581 mg, 1.50 mmol, 1.0 equiv) and

[10] Eisai Co., Ltd. Patent: WO2003/99195 A2, 2003 (p. 58).
[11] J. M. Rodriguez, A. D. Hamilton, *Angew. Chem. Int. Ed.* **2007**, *46*, 8614–8617.

THF (30 mL). Di-*tert*-butyl dicarbonate (663 mg, 3.04 mmol, 2.03 equiv) in THF (5.0 mL) and 4-(dimethylamino)pyridine (18.3 mg, 0.15 mmol, 0.10 equiv) in THF (2.5 mL) were added and the reaction was stirred for 1 h. The solution was concentrated *in vacuo* and purified using automated chromatographic separation on silica gel using a gradient of 5% EtOAc/Hex → 40% EtOAc/Hex to afford the desired compound as a colorless oil (560 mg, 64% yield).

^{1}H NMR (500 MHz, CDCl$_3$): δ 7.59 – 7.50 (m, 2H), 7.07 (t, *J* = 7.9 Hz, 1H), 5.81 (s, 1H), 2.74 (tt, *J* = 7.3, 4.0 Hz, 1H), 1.49 (s, 9H), 1.35 (s, 9H), 1.18 – 1.11 (m, 2H), 0.86 – 0.81 (m, 2H).

^{13}C NMR (125 MHz, CDCl$_3$): δ 163.3, 158.0 (d, *J* = 259.6 Hz), 151.2, 149.8, 148.1, 146.1, 134.0 (d, *J* = 3.7 Hz), 131.2, 126.3 (d, *J* = 22.0 Hz), 122.7 (d, *J* = 12.9 Hz), 103.5, 95.4 (d, *J* = 7.5 Hz), 85.8, 85.7, 28.0, 27.7, 25.5, 8.7, 8.7.

^{19}F NMR (282 MHz, CDCl$_3$): δ –113.9.

HRMS (ESI-TOF): Calculated for $C_{23}H_{28}FIN_3O_6^+$ ([M + H]$^+$): 588.1001, found 588.0997.

FTIR (thin film): 2980, 1724, 1681, 1494, 1345, 1238, 1152, 1119, 861 cm^{-1}.

VI. Product Characterization

General Procedure. A threaded 20 x 125 mm reaction tube containing a magnetic stir bar was transferred to a N_2-filled glovebox. The following solutions were added to the reaction tube: [Ir(dF-CF_3-ppy)$_2$(dtbbpy)]PF_6 (4.5 mg, 4.0 μmol, 0.010 equiv) in DMF (2.0 mL); $NiCl_2$•glyme (8.8 mg, 0.040 mmol, 0.10 equiv) and BiOx (16.8 mg, 0.120 mmol, 0.30 equiv) in DMF (2.0 mL) [prestirred for 5 min]; aryl halide (0.40 mmol, 1.0 equiv) in DMF (2.0 mL); amine (1.2 mmol, 3.0 equiv) in DMF (2.0 mL); KOH (67.3 mg, 1.2 mmol, 3.0 equiv) in DMF (2.0 mL). The reaction mixture was diluted with DMF (10.0 mL), closed with a screw cap containing a Teflon septum, and sealed with electrical tape. The reaction was removed from the glovebox and irradiated with a 34 W blue LED lamp (8 cm away, with cooling fan to keep the reaction at 25 °C). After 24 hours, the reaction was diluted with water (75 mL) and extracted with Et_2O (3 x 75 mL). The combined organics were washed with brine (75 mL), dried over $MgSO_4$, filtered, and concentrated *in vacuo*. Purification of the crude reaction mixture by preparative thin layer chromatography (TLC) using the indicated solvent system provided the desired product.

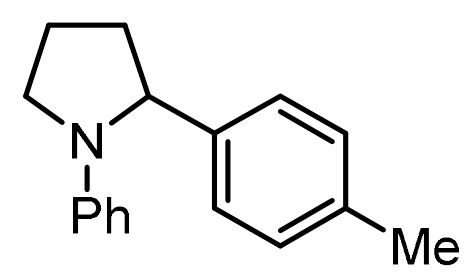

1-phenyl-2-(*p*-tolyl)pyrrolidine (1). Prepared according to General Procedure using *p*-iodotoluene (87.2 mg, 0.40 mmol, 1.0 equiv) and *N*-phenylpyrrolidine (172 μL, 1.2 mmol, 3.0 equiv). The reaction was purified by preparative TLC using 10% DCM/hexanes as eluent. The title compound was isolated as a white solid. Run 1: 80.6 mg, 85% yield. Run 2: 78.5 mg, 83% yield.
^{1}H NMR (500 MHz, $CDCl_3$): δ 7.18 – 7.12 (m, 6H), 6.63 (t, *J* = 7.2 Hz, 1H), 6.49 (d, *J* = 8.1 Hz, 2H), 4.70 (dd, *J* = 8.1, 1.0 Hz, 1H), 3.73 – 3.66 (m, 1H), 3.39 (q, *J* = 9.0 Hz, 1H), 2.42 – 2.34 (m, 1H), 2.32 (s, 3H), 2.11 – 1.83 (m, 3H).
^{13}C NMR (125 MHz, $CDCl_3$): δ 147.3, 141.7, 136.2, 129.3, 129.1, 126.0, 115.8, 112.4, 62.8, 49.2, 36.3, 23.2, 21.2.
HRMS (ESI-TOF): Calculated for $C_{17}H_{20}N^+$ ($[M + H]^+$): 238.1590, found 238.1588.
FTIR (thin film): 2968, 1599, 1505, 1363, 993, 747, 691 cm^{-1}.

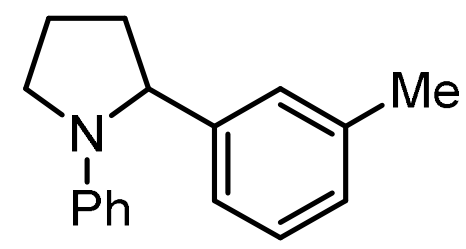

1-phenyl-2-(*m*-tolyl)pyrrolidine (2). Prepared according to General Procedure using *m*-iodotoluene (51.4 µL, 0.40 mmol, 1.0 equiv) and *N*-phenylpyrrolidine (172 µL, 1.2 mmol, 3.0 equiv). The reaction was purified by preparative TLC using 10% DCM/hexanes as eluent. The title compound was isolated as a white solid. Run 1: 62.0 mg, 65% yield. Run 2: 70.0 mg, 74% yield.

^{1}H NMR (500 MHz, $CDCl_3$): δ 7.22 – 7.11 (m, 3H), 7.08 – 6.99 (m, 3H), 6.63 (t, *J* = 7.2 Hz, 1H), 6.50 (d, *J* = 8.1 Hz, 2H), 4.68 (dd, *J* = 8.3, 1.2 Hz, 1H), 3.76 – 3.67 (m, 1H), 3.48 – 3.34 (m, 1H), 2.43 – 2.34 (m, 1H), 2.32 (s, 3H), 2.12 – 2.01 (m, 1H), 2.00 – 1.87 (m, 2H).

^{13}C NMR (125 MHz, $CDCl_3$): δ 147.4, 144.9, 138.2, 129.1, 128.5, 127.5, 126.7, 123.1, 115.8, 112.4, 63.1, 49.3, 36.2, 23.2, 21.7.

HRMS (ESI-TOF): Calculated for $C_{17}H_{20}N^{+}$ ($[M + H]^{+}$): 238.1590, found 238.1586.

FTIR (thin film): 2967, 1600, 1506, 1361, 1186, 993, 782, 747, 691 cm^{-1}.

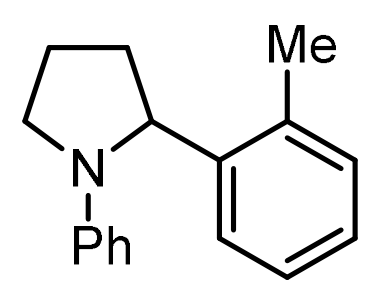

1-phenyl-2-(*o*-tolyl)pyrrolidine (3). Prepared according to General Procedure using *o*-iodotoluene (50.9 µL, 0.40 mmol, 1.0 equiv) and *N*-phenylpyrrolidine (172 µL, 1.2 mmol, 3.0 equiv). The reaction was purified by preparative TLC using 10% DCM/hexanes as eluent. The title compound was isolated as a white solid. Run 1: 24.1 mg, 25% yield. Run 2: 26.0 mg, 27% yield.

^{1}H NMR (500 MHz, $CDCl_3$): δ 7.22 (d, *J* = 7.4 Hz, 1H), 7.16 (t, *J* = 7.7 Hz, 3H), 7.13 – 7.06 (m, 2H), 6.65 (t, *J* = 7.2 Hz, 1H), 6.44 (d, *J* = 8.2 Hz, 2H), 4.86 (d, *J* = 8.2 Hz, 1H), 3.82 – 3.70 (m, 1H), 3.45 (q, *J* = 9.2 Hz, 1H), 2.46 (s, 3H), 2.45 – 2.35 (m, 1H), 2.13 – 1.98 (m, 2H), 1.92 – 1.83 (m, 1H).

^{13}C NMR (125 MHz, $CDCl_3$): δ 147.1, 142.1, 134.2, 130.7, 129.1, 126.6, 126.2, 125.6, 115.8, 112.3, 60.5, 49.1, 34.0, 23.1, 19.4.

HRMS (ESI-TOF): Calculated for $C_{17}H_{20}N^{+}$ ($[M + H]^{+}$): 238.1590, found 238.1585.

FTIR (thin film): 2967, 1599, 1501, 1483, 1365, 993, 745, 691 cm^{-1}.

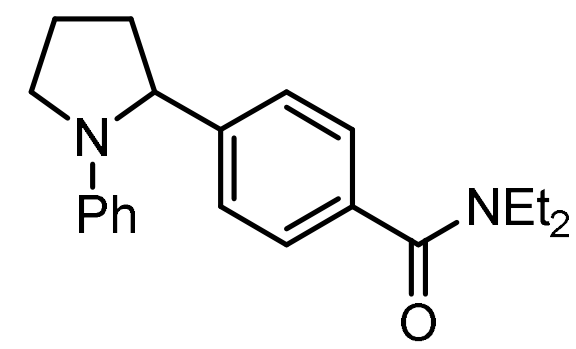

***N*,*N*-diethyl-4-(1-phenylpyrrolidin-2-yl)benzamide (4).** Prepared according to General Procedure using *N*,*N*-diethyl-4-iodobenzamide (121 mg, 0.40 mmol, 1.0 equiv) and *N*-phenylpyrrolidine (172 μL, 1.2 mmol, 3.0 equiv). The reaction was purified by preparative TLC using DCM as eluent. The title compound was isolated as a yellow oil. Run 1: 109 mg, 84% yield. Run 2: 106 mg, 82% yield.

^{1}H NMR (500 MHz, $CDCl_3$): δ 7.34 (d, *J* = 8.1 Hz, 2H), 7.30 – 7.24 (m, 2H), 7.18 (t, *J* = 8.0 Hz, 2H), 6.68 (t, *J* = 7.3 Hz, 1H), 6.51 (d, *J* = 8.0 Hz, 2H), 4.75 (d, *J* = 7.9 Hz, 1H), 3.77 – 3.70 (m, 1H), 3.57 (br s, 2H), 3.43 (q, *J* = 8.7 Hz, 1H), 3.30 (br s, 2H), 2.47 – 2.36 (m, 1H), 2.09 – 1.98 (m, 2H), 1.98 – 1.91 (m, 1H), 1.26 (br s, 3H), 1.14 (br s, 3H).

^{13}C NMR (125 MHz, $CDCl_3$): δ 171.4, 147.1, 146.0, 135.6, 129.1, 126.8, 126.1, 116.0, 112.5, 62.8, 49.2, 43.4, 39.3, 36.1, 23.2, 14.4, 13.0.

HRMS (ESI-TOF): Calculated for $C_{21}H_{27}N_2O^+$ ([M + H]$^+$): 323.2118, found 323.2117.

FTIR (thin film): 2970, 1628, 1598, 1505, 1425, 1363, 1287, 1095, 748, 692 cm^{-1}.

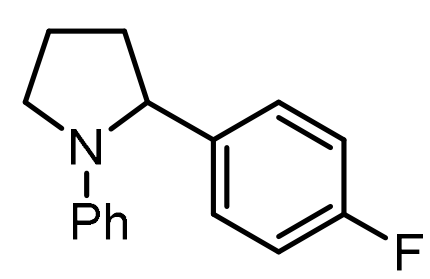

2-(4-fluorophenyl)-1-phenylpyrrolidine (5). Prepared according to General Procedure using *p*-fluoroiodobenzene (46.1 μL, 0.40 mmol, 1.0 equiv) and *N*-phenylpyrrolidine (172 μL, 1.2 mmol, 3.0 equiv). The reaction was purified by preparative TLC using 10% DCM/hexanes as eluent. The title compound was isolated as a colorless oil. Run 1: 67.9 mg, 70% yield. Run 2: 70.0 mg, 73% yield.

^{1}H NMR (500 MHz, $CDCl_3$): δ 7.22 – 7.13 (m, 4H), 6.98 (t, *J* = 8.7 Hz, 2H), 6.65 (t, *J* = 7.2 Hz, 1H), 6.48 (d, *J* = 8.0 Hz, 2H), 4.70 (dd, *J* = 8.1, 1.3 Hz, 1H), 3.74 – 3.67 (m, 1H), 3.40 (q, *J* = 8.9 Hz, 1H), 2.43 – 2.33 (m, 1H), 2.05 – 1.95 (m, 2H), 1.94 – 1.86 (m, 1H).

^{13}C NMR (125 MHz, $CDCl_3$): δ 161.8 (d, *J* = 244.1 Hz), 147.1, 140.3, 129.2, 127.5 (d, *J* = 7.9 Hz), 116.1, 115.4 (d, *J* = 21.3 Hz), 112.5, 62.4, 49.2, 36.3, 23.2.

^{19}F NMR (282 MHz, $CDCl_3$): δ –116.8.

HRMS (ESI-TOF): Calculated for $C_{16}H_{17}FN^+$ ([M + H]$^+$): 242.1340, found 242.1343.

FTIR (thin film): 2969, 1598, 1505, 1361, 1219, 1153, 831, 748, 692 cm^{-1}.

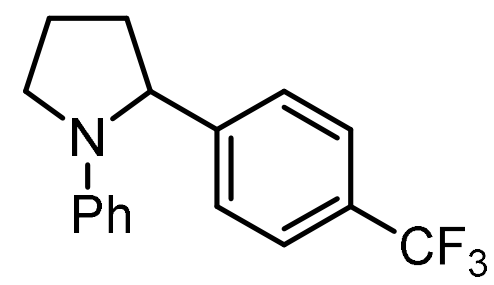

1-phenyl-2-(4-(trifluoromethyl)phenyl)pyrrolidine (6). Prepared according to General Procedure using 1-iodo-4-(trifluoromethyl)benzene (59.1 μL, 0.40 mmol, 1.0 equiv) and *N*-phenylpyrrolidine (172 μL, 1.2 mmol, 3.0 equiv). The reaction was purified by preparative TLC using 10% DCM/hexanes as eluent. The title compound was isolated as a white solid. Run 1: 85.1 mg, 73% yield. Run 2: 82.5 mg, 71% yield.

^{1}H NMR (500 MHz, $CDCl_3$): δ 7.55 (d, *J* = 8.1 Hz, 2H), 7.35 (d, *J* = 8.1 Hz, 2H), 7.19 – 7.13 (m, 2H), 6.67 (t, *J* = 7.3 Hz, 1H), 6.47 (d, *J* = 7.9 Hz, 2H), 4.77 (dd, *J* = 7.6, 1.0 Hz, 1H), 3.77 – 3.71 (m, 1H), 3.43 (q, *J* = 8.4 Hz, 1H), 2.48 – 2.38 (m, 1H), 2.05 – 1.97 (m, 2H), 1.96 – 1.90 (m, 1H).

^{13}C NMR (125 MHz, $CDCl_3$): δ 149.0, 147.0, 129.2, 129.0 (q, *J* = 32.4 Hz), 127.6 (q, *J* = 273.6 Hz), 126.4, 125.6 (q, *J* = 3.5 Hz), 116.4, 112.5, 62.8, 49.3, 36.1, 23.2.

^{19}F NMR (282 MHz, $CDCl_3$): δ –62.3.

HRMS (ESI-TOF): Calculated for $C_{17}H_{17}F_3N^+$ ($[M + H]^+$): 292.1308, found 292.1308.

FTIR (thin film): 2973, 1600, 1505, 1324, 1162, 1122, 1067, 748, 692 cm^{-1}.

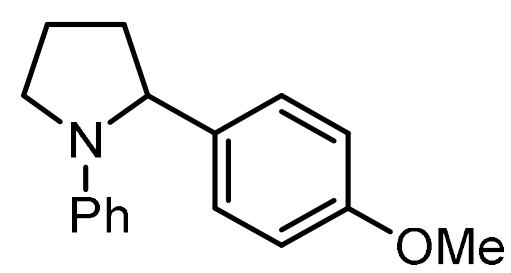

2-(4-methoxyphenyl)-1-phenylpyrrolidine (7). Prepared according to General Procedure using 1-iodo-4-methoxybenzene (93.6 mg, 0.40 mmol, 1.0 equiv) and *N*-phenylpyrrolidine (172 μL, 1.2 mmol, 3.0 equiv). The reaction was purified by preparative TLC using 10% DCM/hexanes as eluent. The title compound was isolated as a colorless oil which contained 5% hydrodehalogenation product as an inseparable impurity. Run 1: 83.3 mg, 82% yield. Run 2: 82.3 mg, 81% yield.

^{1}H NMR (500 MHz, $CDCl_3$): δ 7.18 – 7.11 (m, 4H), 6.84 (d, *J* = 8.6 Hz, 2H), 6.63 (t, *J* = 7.2 Hz, 1H), 6.50 (d, *J* = 7.9 Hz, 2H), 4.69 (d, *J* = 7.5 Hz, 1H), 3.78 (s, 3H), 3.73 – 3.66 (m, 1H), 3.39 (q, *J* = 9.0 Hz, 1H), 2.41 – 2.29 (m, 1H), 2.08 – 1.94 (m, 2H), 1.94 – 1.87 (m, 1H).

^{13}C NMR (125 MHz, $CDCl_3$): δ 158.4, 147.3, 136.7, 129.1, 127.0, 115.8, 113.9, 112.4, 62.4, 55.4, 49.1, 36.3, 23.2.

HRMS (ESI-TOF): Calculated for $C_{17}H_{20}NO^+$ ($[M + H]^+$): 254.1539, found 254.1535.

FTIR (thin film): 2966, 1599, 1505, 1363, 1246, 1174, 1035, 747, 692 cm^{-1}.

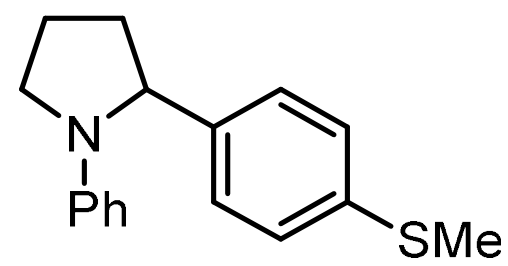

2-(4-(methylthio)phenyl)-1-phenylpyrrolidine (8). Prepared according to General Procedure using 1-iodo-4-(methylsulfanyl)benzene (100 mg, 0.40 mmol, 1.0 equiv) and *N*-phenylpyrrolidine (172 µL, 1.2 mmol, 3.0 equiv). The reaction was purified by preparative TLC using 10% DCM/hexanes as eluent. The title compound was isolated as a white solid. Run 1: 78.0 mg, 72% yield. Run 2: 75.4 mg, 70% yield.

^{1}H NMR (500 MHz, $CDCl_3$): δ 7.22 – 7.11 (m, 6H), 6.64 (t, *J* = 7.3 Hz, 1H), 6.48 (d, *J* = 8.2 Hz, 2H), 4.68 (dd, *J* = 8.2, 1.3 Hz, 1H), 3.73 – 3.66 (m, 1H), 3.39 (q, *J* = 9.0 Hz, 1H), 2.46 (s, 3H), 2.42 – 2.31 (m, 1H), 2.05 – 1.95 (m, 2H), 1.94 – 1.87 (m, 1H).

^{13}C NMR (125 MHz, $CDCl_3$): δ 147.2, 141.9, 136.3, 129.1, 127.0, 126.6, 116.0, 112.4, 62.6, 49.2, 36.2, 23.2, 16.2.

HRMS (ESI-TOF): Calculated for $C_{17}H_{20}NS^+$ ([M + H]$^+$): 270.1311, found 270.1315.

FTIR (thin film): 2968, 1597, 1505, 1363, 1183, 816, 747, 692 cm^{-1}.

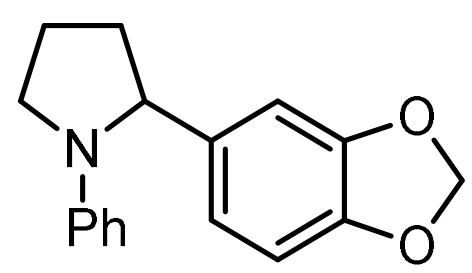

2-(benzo[*d*][1,3]dioxol-5-yl)-1-phenylpyrrolidine (9). Prepared according to General Procedure using 1-iodo-3,4-methylenedioxybenzene (50.6 µL, 0.40 mmol, 1.0 equiv) and *N*-phenylpyrrolidine (172 µL, 1.2 mmol, 3.0 equiv). The reaction was purified by preparative TLC using 10% DCM/hexanes as eluent. The title compound was isolated as a brown oil which contained 6% hydrodehalogenation product as an inseparable impurity. Run 1: 83.8 mg, 78% yield. Run 2: 88.5 mg, 83% yield.

^{1}H NMR (500 MHz, $CDCl_3$): δ 7.15 (t, *J* = 7.9 Hz, 2H), 6.75 – 6.68 (m, 3H), 6.64 (t, *J* = 7.3 Hz, 1H), 6.50 (d, *J* = 8.0 Hz, 2H), 5.92 (d, *J* = 6.6 Hz, 2H), 4.63 (dd, *J* = 8.2, 1.6 Hz, 1H), 3.71 – 3.65 (m, 1H), 3.37 (q, *J* = 9.1 Hz, 1H), 2.38 – 2.29 (m, 1H), 2.07 – 1.93 (m, 2H), 1.92 – 1.86 (m, 1H).

^{13}C NMR (125 MHz, $CDCl_3$): δ 147.9, 147.2, 146.3, 139.0, 129.1, 118.9, 116.0, 112.5, 108.4, 106.6, 101.0, 62.9, 49.2, 36.3, 23.2.

HRMS (ESI-TOF): Calculated for $C_{17}H_{18}NO_2^+$ ([M + H]$^+$): 268.1332, found 268.1327.

FTIR (thin film): 2879, 1598, 1503, 1485, 1357, 1235, 1038, 748, 692 cm^{-1}.

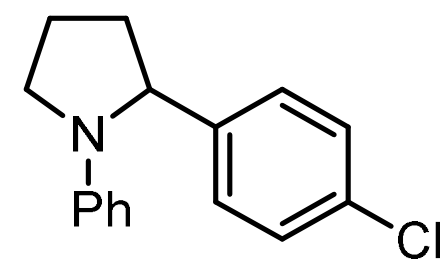

2-(4-chlorophenyl)-1-phenylpyrrolidine (10). Prepared according to General Procedure using *p*-chloroiodobenzene (95.4 mg, 0.40 mmol, 1.0 equiv) and *N*-phenylpyrrolidine (172 µL, 1.2 mmol, 3.0 equiv). The reaction was purified by preparative TLC using 10% DCM/hexanes as eluent. The title compound was isolated as a white solid. Run 1: 75.0 mg, 73% yield. Run 2: 76.7 mg, 74% yield.

^{1}H NMR (500 MHz, CDCl$_3$): δ 7.28 (t, *J* = 4.2 Hz, 2H), 7.20 – 7.14 (m, 4H), 6.67 (t, *J* = 7.3 Hz, 1H), 6.48 (d, *J* = 7.9 Hz, 2H), 4.70 (dd, *J* = 8.3, 1.8 Hz, 1H), 3.75 – 3.69 (m, 1H), 3.41 (q, *J* = 8.5 Hz, 1H), 2.45 – 2.35 (m, 1H), 2.06 – 1.97 (m, 2H), 1.95 – 1.88 (m, 1H).

^{13}C NMR (125 MHz, CDCl$_3$): δ 147.1, 143.3, 132.3, 129.2, 128.7, 127.5, 116.2, 112.5, 62.5, 49.2, 36.2, 23.2.

HRMS (ESI-TOF): Calculated for $C_{16}H_{17}ClN^+$ ($[M + H]^+$): 258.1044, found 258.1047.

FTIR (thin film): 2972, 1598, 1504, 1488, 1362, 1013, 748, 692 cm^{-1}.

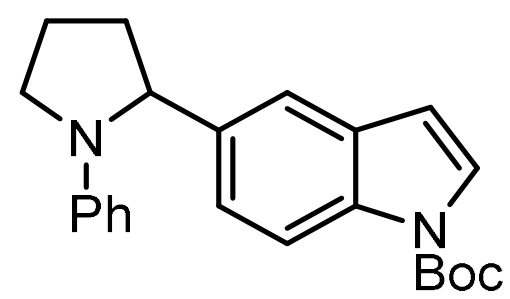

***tert*-butyl 5-(1-phenylpyrrolidin-2-yl)-1*H*-indole-1-carboxylate (11).** Prepared according to General Procedure using *tert*-butyl 5-iodo-1*H*-indole-1-carboxylate (137 mg, 0.40 mmol, 1.0 equiv) and *N*-phenylpyrrolidine (172 µL, 1.2 mmol, 3.0 equiv). The reaction was purified twice by preparative TLC: Plate 1 used 10% EtOAc/hexanes as eluent; Plate 2 used 20% DCM/hexanes as eluent. The title compound was isolated as a white solid. Run 1: 72.9 mg, 50% yield. Run 2: 72.8 mg, 50% yield. The final product contained 10% (5% yield) of an inseparable impurity.

^{1}H NMR (500 MHz, CDCl$_3$): δ 8.19 – 7.94 (m, 1H), 7.57 (s, 1H), 7.39 (s, 1H), 7.21 (d, *J* = 8.6 Hz, 1H), 7.13 (t, *J* = 7.9 Hz, 2H), 6.62 (t, *J* = 7.3 Hz, 1H), 6.52 (d, *J* = 8.3 Hz, 2H), 6.49 (d, *J* = 3.6 Hz, 1H), 4.82 (d, *J* = 7.8 Hz, 1H), 3.78 – 3.72 (m, 1H), 3.46 – 3.40 (m, 1H), 2.46 – 2.36 (m, 1H), 2.13 – 2.03 (m, 1H), 2.03 – 1.93 (m, 2H), 1.66 (s, 9H).

^{13}C NMR (125 MHz, CDCl$_3$): δ 147.3, 140.2, 139.2, 134.2, 130.9, 129.1, 126.3, 122.5, 118.1, 115.8, 115.3, 112.5, 107.5, 83.7, 63.1, 49.3, 36.6, 28.3, 23.2.

HRMS (ESI-TOF): Calculated for $C_{23}H_{27}N_2O_2^+$ ($[M + H]^+$): 363.2067, found 363.2066.

FTIR (thin film): 2973, 1733, 1598, 1504, 1470, 1358, 1160, 747, 692 cm^{-1}.

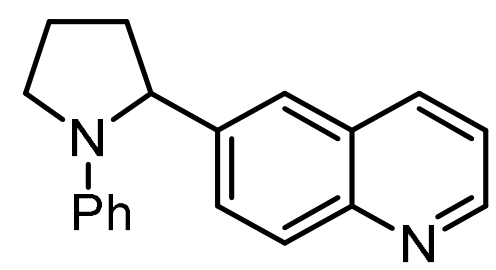

6-(1-phenylpyrrolidin-2-yl)quinoline (12). Prepared according to General Procedure using 6-iodoquinoline (102 mg, 0.40 mmol, 1.0 equiv) and *N*-phenylpyrrolidine (172 µL, 1.2 mmol, 3.0 equiv). The crude reaction mixture was purified using automated chromatographic separation on silica gel using a gradient of 5% EtOAc/Hex → 60% EtOAc/Hex to afford the desired compound as a brown oil. Run 1: 74.7 mg, 68% yield. Run 2: 80.0 mg, 73% yield.

^{1}H NMR (300 MHz, $CDCl_3$): δ 8.87 (dd, *J* = 4.2, 1.5 Hz, 1H), 8.08 (t, *J* = 7.6 Hz, 2H), 7.68 – 7.60 (m, 2H), 7.36 (dd, *J* = 8.3, 4.2 Hz, 1H), 7.18 – 7.12 (m, 2H), 6.66 (t, *J* = 7.3 Hz, 1H), 6.54 (d, *J* = 8.1 Hz, 2H), 4.89 (d, *J* = 7.6 Hz, 1H), 3.84 – 3.77 (m, 1H), 3.51 – 3.43 (m, 1H), 2.52 – 2.42 (m, 1H), 2.11 – 1.99 (m, 3H).

^{13}C NMR (125 MHz, $CDCl_3$): δ 150.0, 147.7, 147.2, 143.0, 136.2, 129.9, 129.2, 128.4, 128.3, 124.2, 121.3, 116.2, 112.6, 63.1, 49.4, 35.9, 23.3.

HRMS (ESI-TOF): Calculated for $C_{19}H_{19}N_2^+$ ($[M + H]^+$): 275.1543, found 275.1541.

FTIR (thin film): 2970, 1598, 1503, 1361, 1185, 838, 748, 692 cm^{-1}.

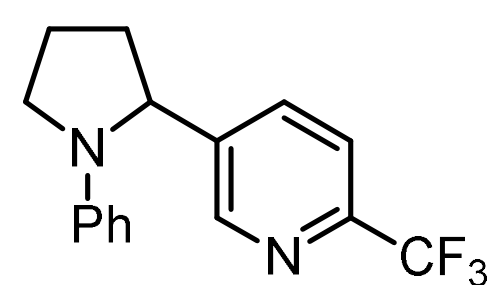

5-(1-phenylpyrrolidin-2-yl)-2-(trifluoromethyl)pyridine (13). Prepared according to General Procedure using 5-bromo-2-(trifluoromethyl)pyridine (90.4 mg, 0.40 mmol, 1.0 equiv) and *N*-phenylpyrrolidine (172 µL, 1.2 mmol, 3.0 equiv). The reaction was purified twice by preparative TLC: Plate 1 used 30% EtOAc/hexanes as eluent; Plate 2 used DCM as eluent. The title compound was isolated as a white solid. Run 1: 49.0 mg, 42% yield. Run 2: 44.9 mg, 38% yield.

^{1}H NMR (500 MHz, $CDCl_3$): δ 8.67 (s, 1H), 7.67 (d, *J* = 7.9 Hz, 1H), 7.59 (d, *J* = 8.0 Hz, 1H), 7.17 (t, *J* = 7.7 Hz, 2H), 6.70 (t, *J* = 7.2 Hz, 1H), 6.46 (d, *J* = 8.1 Hz, 2H), 4.83 (d, *J* = 8.3 Hz, 1H), 3.76 (t, *J* = 7.5 Hz, 1H), 3.44 (q, *J* = 9.0, 8.5 Hz, 1H), 2.55 – 2.44 (m, 1H), 2.11 – 2.04 (m, 1H), 2.04 – 1.92 (m, 2H).

^{13}C NMR (125 MHz, $CDCl_3$): δ 148.7, 146.9 (q, *J* = 34.7 Hz), 146.6, 143.6, 135.1, 129.4, 121.8 (q, *J* = 274.0 Hz), 120.4 (q, *J* = 2.5 Hz), 116.9, 112.7, 60.6, 49.3, 35.9, 23.2.

^{19}F NMR (282 MHz, $CDCl_3$): δ –67.7.

HRMS (ESI-TOF): Calculated for $C_{16}H_{16}F_3N_2^+$ ($[M + H]^+$): 293.1260, found 293.1259.

FTIR (thin film): 2973, 1600, 1505, 1338, 1135, 1083, 749, 693 cm^{-1}.

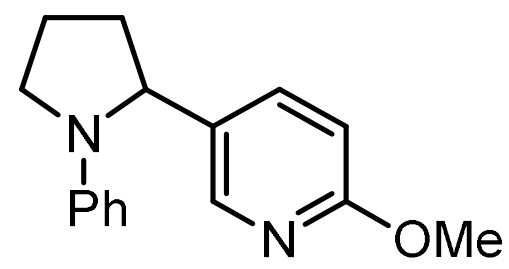

2-methoxy-5-(1-phenylpyrrolidin-2-yl)pyridine (14). Prepared according to General Procedure using 5-bromo-2-methoxypyridine (51.8 µL, 0.40 mmol, 1.0 equiv) and *N*-phenylpyrrolidine (172 µL, 1.2 mmol, 3.0 equiv). The reaction was purified by preparative TLC using 30% EtOAc/hexanes as eluent. The title compound was isolated as a colorless oil. Run 1: 40.9 mg, 40% yield. Run 2: 38.9 mg, 38% yield.

^{1}H NMR (500 MHz, CDCl$_3$): δ 8.08 – 8.01 (m, 1H), 7.41 (dd, *J* = 8.6, 1.7 Hz, 1H), 7.15 (t, *J* = 7.8 Hz, 2H), 6.71 – 6.61 (m, 2H), 6.49 (d, *J* = 8.0 Hz, 2H), 4.71 (d, *J* = 7.1 Hz, 1H), 3.91 (s, 3H), 3.73 – 3.65 (m, 1H), 3.39 (q, *J* = 8.4 Hz, 1H), 2.43 – 2.33 (m, 1H), 2.07 – 1.97 (m, 2H), 1.94 – 1.86 (m, 1H).

^{13}C NMR (125 MHz, CDCl$_3$): δ 163.4, 147.0, 144.7, 136.9, 132.6, 129.2, 116.2, 112.5, 110.8, 60.3, 53.5, 49.1, 36.1, 23.2.

HRMS (ESI-TOF): Calculated for $C_{16}H_{19}N_2O^+$ ($[M + H]^+$): 255.1492, found 255.1495.

FTIR (thin film): 2944, 1600, 1505, 1490, 1358, 1280, 1027, 748, 692 cm^{-1}.

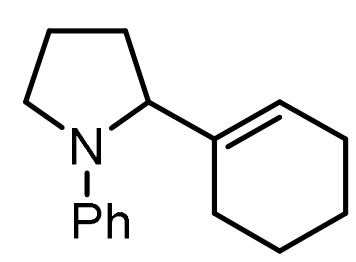

2-(cyclohex-1-en-1-yl)-1-phenylpyrrolidine (15). Prepared according to General Procedure using cyclohex-1-en-1-yl trifluoromethanesulfonate (70.0 µL, 0.40 mmol, 1.0 equiv) and *N*-phenylpyrrolidine (172 µL, 1.2 mmol, 3.0 equiv). The reaction was purified by preparative TLC using 10% DCM/hexanes as eluent. The title compound was isolated as a colorless oil. Run 1: 62.7 mg, 69% yield. Run 2: 60.5 mg, 67% yield.

^{1}H NMR (500 MHz, CDCl$_3$): δ 7.23 – 7.16 (m, 2H), 6.64 (t, *J* = 7.2 Hz, 1H), 6.54 (d, *J* = 8.0 Hz, 2H), 5.52 – 5.47 (m, 1H), 3.89 (d, *J* = 7.4 Hz, 1H), 3.51 – 3.44 (m, 1H), 3.26 (q, *J* = 8.6 Hz, 1H), 2.05 – 1.89 (m, 7H), 1.86 – 1.80 (m, 1H), 1.69 – 1.60 (m, 2H), 1.56 (dt, *J* = 9.5, 4.8 Hz, 2H).

^{13}C NMR (125 MHz, CDCl$_3$): δ 147.6, 137.8, 129.0, 121.2, 115.3, 112.0, 64.6, 48.9, 32.0, 25.7, 25.0, 23.7, 23.0, 22.9.

HRMS (ESI-TOF): Calculated for $C_{16}H_{22}N^+$ ($[M + H]^+$): 228.1747, found 228.1743.

FTIR (thin film): 2927, 1597, 1505, 1359, 1186, 993, 746, 690 cm^{-1}.

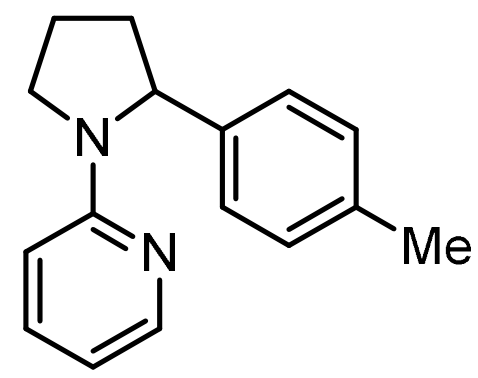

2-(2-(*p*-tolyl)pyrrolidin-1-yl)pyridine (16). Prepared according to General Procedure using *p*-iodotoluene (87.2 mg, 0.40 mmol, 1.0 equiv) and 2-(pyrrolidin-1-yl)pyridine (178 µL, 1.2 mmol, 3.0 equiv). The reaction was purified by preparative TLC using 30% EtOAc/hexanes as eluent. The title compound was isolated as a yellow solid. Run 1: 67.0 mg, 70% yield. Run 2: 56.9 mg, 60% yield.

^{1}H NMR (500 MHz, $CDCl_3$): δ 8.16 (dd, *J* = 3.9, 1.0 Hz, 1H), 7.31 – 7.24 (m, 1H), 7.10 (s, 4H), 6.52 – 6.46 (m, 1H), 6.15 (d, *J* = 8.5 Hz, 1H), 4.86 (d, *J* = 7.0 Hz, 1H), 3.93 – 3.85 (m, 1H), 3.74 – 3.66 (m, 1H), 2.45 – 2.36 (m, 1H), 2.32 (s, 3H), 2.04 – 1.92 (m, 3H).

^{13}C NMR (125 MHz, $CDCl_3$): δ 157.2, 148.3, 141.1, 136.8, 136.4, 129.3, 125.9, 111.6, 107.6, 61.6, 48.2, 36.3, 23.0, 21.2.

HRMS (ESI-TOF): Calculated for $C_{16}H_{19}N_2^+$ ($[M + H]^+$): 239.1543, found 239.1541.

FTIR (thin film): 2969, 1597, 1483, 1440, 1378, 992, 769, 733 cm^{-1}.

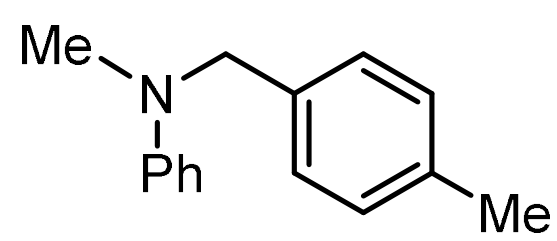

***N*-methyl-*N*-(4-methylbenzyl)aniline (17).** Prepared according to General Procedure using *p*-iodotoluene (87.2 mg, 0.40 mmol, 1.0 equiv) and dimethylaniline (152 µL, 1.2 mmol, 3.0 equiv). The reaction was purified by preparative TLC using 10% DCM/hexanes as eluent. The title compound was isolated as a colorless oil. Run 1: 60.5 mg, 72% yield. Run 2: 67.5 mg, 80% yield.

^{1}H NMR (500 MHz, $CDCl_3$): δ 7.22 (t, *J* = 8.0 Hz, 2H), 7.13 (s, 4H), 6.76 (d, *J* = 8.4 Hz, 2H), 6.71 (t, *J* = 7.3 Hz, 1H), 4.50 (s, 2H), 3.00 (s, 3H), 2.33 (s, 3H).

^{13}C NMR (125 MHz, $CDCl_3$): δ 149.9, 136.6, 136.0, 129.4, 129.3, 126.8, 116.5, 112.4, 56.5, 38.6, 21.2.

HRMS (ESI-TOF): Calculated for $C_{15}H_{18}N^+$ ($[M + H]^+$): 212.1434, found 212.1432.

FTIR (thin film): 2921, 1599, 1506, 1372, 1350, 1115, 747, 691 cm^{-1}.

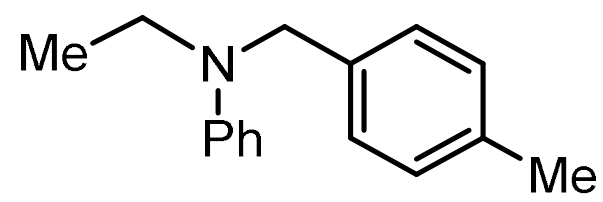

***N*-ethyl-*N*-(4-methylbenzyl)aniline (18).** Prepared according to General Procedure using *p*-iodotoluene (87.2 mg, 0.40 mmol, 1.0 equiv) and *N*-ethyl-*N*-methylaniline (162 µL, 1.2 mmol, 3.0 equiv). The reaction was purified by preparative TLC using 10% DCM/hexanes as eluent. The title compound was isolated as a colorless oil. Run 1: 77.1 mg, 86% yield. Run 2: 72.9 mg, 81% yield.

^{1}H NMR (500 MHz, $CDCl_3$): δ 7.22 – 7.18 (m, 2H), 7.17 – 7.10 (m, 4H), 6.71 (d, *J* = 8.3 Hz, 2H), 6.67 (t, *J* = 7.2 Hz, 1H), 4.50 (s, 2H), 3.47 (q, *J* = 7.0 Hz, 2H), 2.34 (s, 3H), 1.21 (t, *J* = 7.1 Hz, 3H).

^{13}C NMR (125 MHz, $CDCl_3$): δ 148.5, 136.3, 136.1, 129.2, 129.2, 126.5, 115.9, 112.1, 53.6, 45.0, 21.1, 12.1.

HRMS (ESI-TOF): Calculated for $C_{16}H_{20}N^+$ ($[M + H]^+$): 226.1590, found 226.1592.

FTIR (thin film): 2970, 1599, 1506, 1353, 1244, 1038, 746, 692 cm^{-1}.

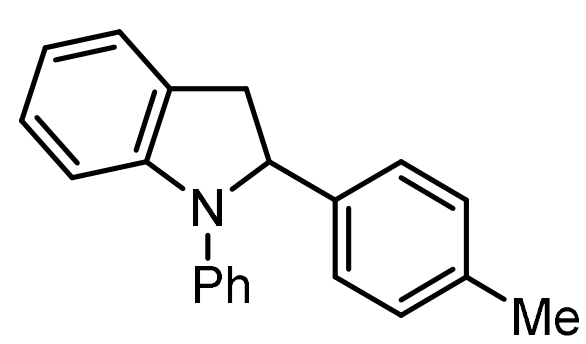

1-phenyl-2-(*p*-tolyl)indoline (19). Prepared according to General Procedure using *p*-iodotoluene (87.2 mg, 0.40 mmol, 1.0 equiv) and *N*-phenylindoline (234 µL, 1.2 mmol, 3.0 equiv). The reaction was purified by two runs of preparative SFC in sequence: The first run used 10% EtOH/CO_2 as eluent (Chiralpak AS-H, Peak 1 taken) and the second run used 10% EtOH/CO_2 as eluent (Chiralpak IA). The title compound was isolated as a brown solid. Run 1: 60.1 mg, 53% yield. Run 2: 78.3 mg, 69% yield.

^{1}H NMR (500 MHz, $CDCl_3$): δ 7.25 – 7.19 (m, 4H), 7.16 (d, *J* = 7.9 Hz, 2H), 7.14 – 7.07 (m, 5H), 6.92 (t, *J* = 7.2 Hz, 1H), 6.81 – 6.76 (m, 1H), 5.24 (dd, *J* = 9.5, 6.3 Hz, 1H), 3.66 (dd, *J* = 15.6, 9.6 Hz, 1H), 2.96 (dd, *J* = 15.6, 6.2 Hz, 1H), 2.30 (s, 3H).

^{13}C NMR (125 MHz, $CDCl_3$): δ 148.1, 143.7, 140.6, 136.9, 129.5, 129.3, 129.1, 127.4, 126.3, 125.2, 121.8, 119.4, 108.8, 67.9, 39.8, 21.2. (One peak is obscured due to overlap.)

HRMS (ESI-TOF): Calculated for $C_{21}H_{20}N^+$ ($[M + H]^+$): 286.1590, found 286.1589.

FTIR (thin film): 2920, 1592, 1500, 1480, 1453, 1378, 745, 695, 668 cm^{-1}.

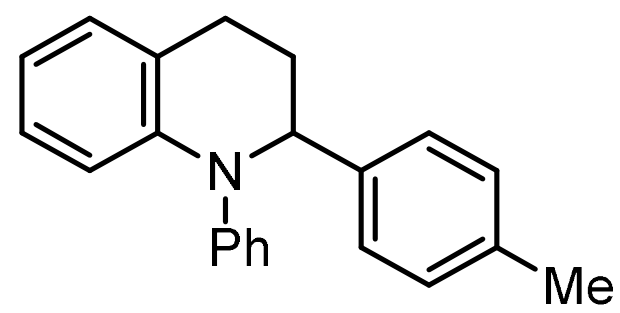

1-phenyl-2-(*p*-tolyl)-1,2,3,4-tetrahydroquinoline (20). Prepared according to General Procedure using *p*-iodotoluene (87.2 mg, 0.40 mmol, 1.0 equiv) and *N*-phenyl-1,2,3,4-tetrahydroquinoline (251 mg, 1.2 mmol, 3.0 equiv). The reaction was purified by preparative SFC using 10% EtOH/CO_2 as eluent (Chiralpak IA). The title compound was isolated as a dark purple oil. Run 1: 68.5 mg, 57% yield. Run 2: 82.6 mg, 69% yield.

<u>^{1}H NMR (500 MHz, CDCl$_3$):</u> δ 7.27 – 7.22 (m, 2H), 7.21 – 7.15 (m, 4H), 7.08 (d, *J* = 7.9 Hz, 2H), 7.06 – 6.97 (m, 3H), 6.88 (d, *J* = 8.2 Hz, 1H), 6.71 (t, *J* = 7.3 Hz, 1H), 4.92 (t, *J* = 4.1 Hz, 1H), 2.69 (dt, *J* = 16.1, 4.2 Hz, 1H), 2.63 – 2.54 (m, 1H), 2.37 – 2.28 (m, 4H), 2.17 – 2.10 (m, 1H).

<u>^{13}C NMR (125 MHz, CDCl$_3$):</u> δ 148.1, 143.9, 141.0, 136.3, 129.4, 129.4, 129.2, 126.7, 126.5, 125.0, 124.2, 123.9, 118.1, 115.8, 63.0, 29.1, 23.8, 21.2.

<u>HRMS (ESI-TOF):</u> Calculated for $C_{22}H_{22}N^+$ ($[M + H]^+$): 300.1747, found 300.1748.

<u>FTIR (thin film):</u> 2925, 1592, 1494, 1459, 1381, 1300, 747, 697 cm^{-1}.

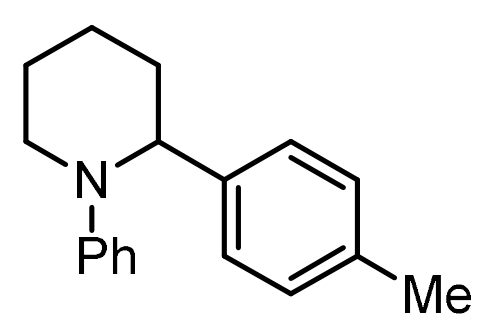

1-phenyl-2-(*p*-tolyl)piperidine (21). Prepared according to General Procedure using *p*-iodotoluene (87.2 mg, 0.40 mmol, 1.0 equiv) and *N*-phenylpiperidine (194 µL, 1.2 mmol, 3.0 equiv). The reaction was purified by preparative TLC using 30% DCM/hexanes as eluent. The title compound was isolated as a brown oil. Run 1: 35.3 mg, 35% yield. Run 2: 30.0 mg, 30% yield.

<u>^{1}H NMR (500 MHz, CDCl$_3$):</u> δ 7.15 (t, *J* = 8.0 Hz, 4H), 7.05 (d, *J* = 7.9 Hz, 2H), 6.90 (d, *J* = 7.9 Hz, 2H), 6.76 (t, *J* = 7.3 Hz, 1H), 4.53 – 4.47 (m, 1H), 3.43 – 3.36 (m, 1H), 3.31 – 3.23 (m, 1H), 2.28 (s, 3H), 2.03 – 1.91 (m, 2H), 1.80 – 1.73 (m, 2H), 1.72 – 1.63 (m, 1H), 1.60 – 1.51 (m, 1H).

<u>^{13}C NMR (125 MHz, CDCl$_3$):</u> δ 152.0, 140.5, 135.8, 129.1, 128.9, 127.2, 119.5, 118.6, 60.6, 50.4, 33.5, 25.8, 22.1, 21.1.

<u>HRMS (ESI-TOF):</u> Calculated for $C_{18}H_{22}N^+$ ($[M + H]^+$): 252.1747, found 252.1745.

<u>FTIR (thin film):</u> 2932, 1597, 1503, 1450, 1242, 928, 813, 748, 696 cm^{-1}.

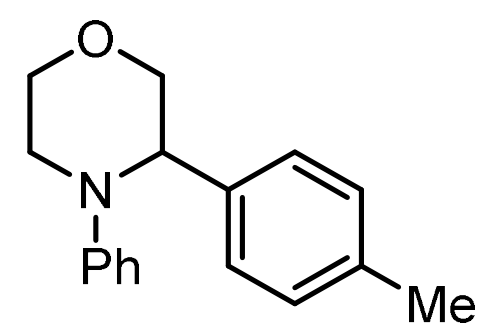

4-phenyl-3-(*p*-tolyl)morpholine (22). Prepared according to General Procedure using *p*-iodotoluene (87.2 mg, 0.40 mmol, 1.0 equiv) and *N*-phenylmorpholine (196 mg, 1.2 mmol, 3.0 equiv). The reaction was purified by preparative TLC using 10% EtOAc/hexanes as eluent. The title compound was isolated as a white solid. Run 1: 68.2 mg, 67% yield. Run 2: 72.3 mg, 71% yield.

^{1}H NMR (500 MHz, $CDCl_3$): δ 7.19 (d, *J* = 8.0 Hz, 2H), 7.15 (t, *J* = 7.8 Hz, 2H), 7.02 (d, *J* = 7.9 Hz, 2H), 6.93 (d, *J* = 8.0 Hz, 2H), 6.85 (t, *J* = 7.3 Hz, 1H), 4.32 (dd, *J* = 8.0, 3.4 Hz, 1H), 4.00 – 3.91 (m, 3H), 3.66 (dd, *J* = 11.5, 8.1 Hz, 1H), 3.44 (dt, *J* = 12.3, 3.3 Hz, 1H), 3.16 – 3.08 (m, 1H), 2.25 (s, 3H).

^{13}C NMR (125 MHz, $CDCl_3$): δ 151.2, 136.8, 136.3, 129.2, 128.9, 127.8, 121.8, 121.3, 73.4, 67.8, 61.5, 53.1, 21.2.

HRMS (ESI-TOF): Calculated for $C_{17}H_{20}NO^+$ ([M + H]$^+$): 254.1539, found 254.1543.

FTIR (thin film): 2851, 1599, 1493, 1215, 1119, 942, 814, 768, 696 cm^{-1}.

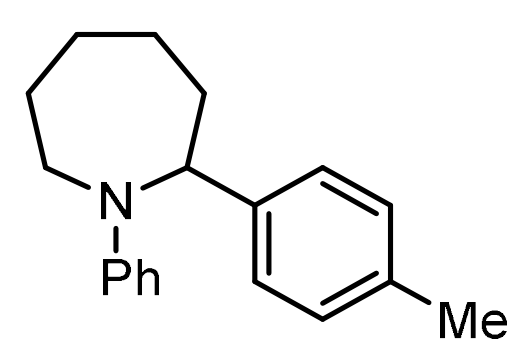

1-phenyl-2-(*p*-tolyl)azepane (23). Prepared according to General Procedure using *p*-iodotoluene (87.2 mg, 0.40 mmol, 1.0 equiv) and *N*-phenylazepane (210 µL, 1.2 mmol, 3.0 equiv). The reaction was purified by preparative TLC using 10% DCM/hexanes as eluent. The title compound was isolated as a white solid. Run 1: 50.3 mg, 50% yield. Run 2: 54.3 mg, 54% yield.

^{1}H NMR (300 MHz, $CDCl_3$): δ 7.18 – 7.07 (m, 6H), 6.61 (t, *J* = 8.1 Hz, 3H), 4.58 (dd, *J* = 11.8, 5.9 Hz, 1H), 3.82 (d, *J* = 15.7 Hz, 1H), 3.56 – 3.47 (m, 1H), 2.51 – 2.41 (m, 1H), 2.32 (s, 3H), 1.96 – 1.84 (m, 2H), 1.84 – 1.68 (m, 3H), 1.46 (q, *J* = 12.1 Hz, 1H), 1.40 – 1.30 (m, 1H).

^{13}C NMR (125 MHz, $CDCl_3$): δ 149.2, 141.2, 136.1, 129.5, 129.2, 125.8, 115.4, 111.4, 62.7, 45.4, 38.9, 29.9, 28.4, 26.6, 21.2.

HRMS (ESI-TOF): Calculated for $C_{19}H_{24}N^+$ ([M + H]$^+$): 266.1903, found 266.1901.

FTIR (thin film): 2924, 1597, 1505, 1387, 745, 693 cm^{-1}.

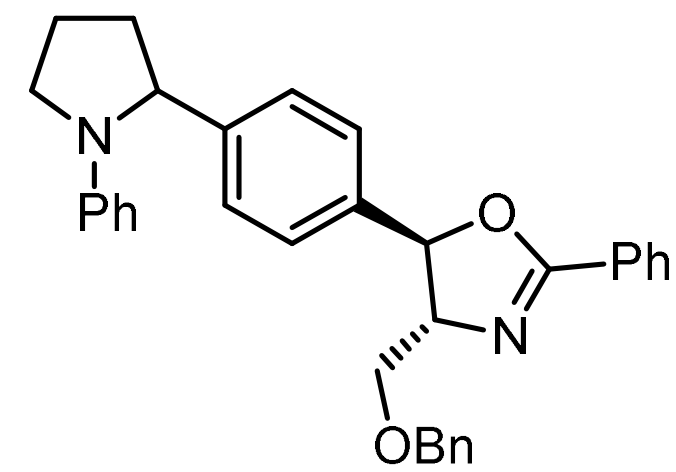

(4*R*,5*R*)-4-((benzyloxy)methyl)-2-phenyl-5-(4-(1-phenylpyrrolidin-2-yl)phenyl)-4,5-dihydrooxazole (24). Prepared according to General Procedure using (4*R*,5*R*)-4-((benzyloxy)methyl)-5-(4-iodophenyl)-2-phenyl-4,5-dihydrooxazole (188 mg, 0.40 mmol, 1.0 equiv) and *N*-phenylpyrrolidine (172 μL, 1.2 mmol, 3.0 equiv). The reaction was purified by preparative SFC using 45% EtOH/CO_2 as eluent (Chiralcel OD-H). The title compound was isolated as a brown oil (155 mg, 79% yield). The dr of the product is roughly 1:1 (integration of the UV trace in the SFC chromatogram gave a 1.06:1 ratio). The diastereomers are identical by ^{1}H and ^{13}C NMR.

^{1}H NMR (300 MHz, $CDCl_3$): δ 8.03 (d, *J* = 7.2 Hz, 2H), 7.50 (t, *J* = 7.4 Hz, 1H), 7.43 (t, *J* = 7.5 Hz, 2H), 7.36 – 7.24 (m, 7H), 7.21 (d, *J* = 8.1 Hz, 2H), 7.14 (t, *J* = 7.9 Hz, 2H), 6.64 (t, *J* = 7.3 Hz, 1H), 6.48 (d, *J* = 8.0 Hz, 2H), 5.50 (d, *J* = 6.8 Hz, 1H), 4.72 (d, *J* = 7.2 Hz, 1H), 4.67 – 4.57 (m, 2H), 4.36 (td, *J* = 6.8, 4.3 Hz, 1H), 3.84 (dd, *J* = 9.7, 4.2 Hz, 1H), 3.72 – 3.63 (m, 2H), 3.40 (q, *J* = 8.9 Hz, 1H), 2.43 – 2.31 (m, 1H), 2.06 – 1.95 (m, 2H), 1.95 – 1.88 (m, 1H).

^{13}C NMR (125 MHz, $CDCl_3$): δ 164.2, 147.2, 144.9, 139.3, 138.2, 131.7, 129.1, 128.6, 128.5, 128.5, 127.9, 127.8, 127.7, 126.4, 126.0, 116.0, 112.4, 84.0, 75.0, 73.5, 71.9, 62.8, 49.2, 36.1, 23.2.

HRMS (ESI-TOF): Calculated for $C_{33}H_{33}N_2O_2^+$ ($[M + H]^+$): 489.2537, found 489.2535.

FTIR (thin film): 2861, 1650, 1599, 1505, 1365, 1063, 747, 694 cm^{-1}.

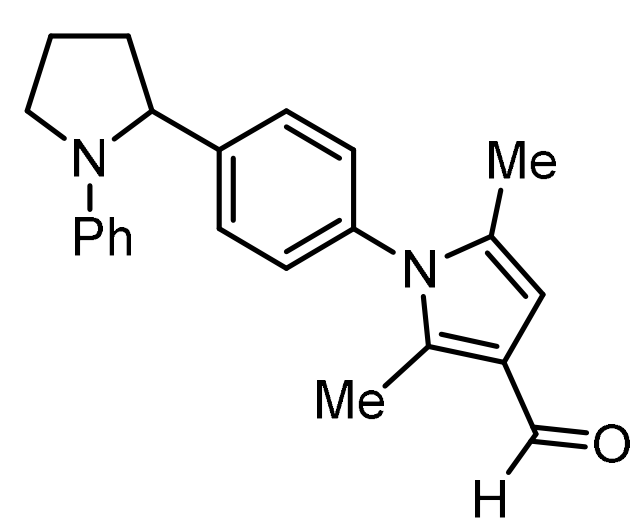

2,5-dimethyl-1-(4-(1-phenylpyrrolidin-2-yl)phenyl)-1*H*-pyrrole-3-carbaldehyde (25). Prepared according to General Procedure using 1-(4-iodophenyl)-2,5-dimethyl-1*H*-pyrrole-3-carbaldehyde (130 mg, 0.40 mmol, 1.0 equiv) and *N*-phenylpyrrolidine (172 μL, 1.2 mmol, 3.0 equiv). The reaction was purified by preparative SFC using 30% EtOH/CO_2 as eluent (Chiralcel AS-H). The title compound was isolated as a brown solid (87.2 mg, 63% yield).

^{1}H NMR (500 MHz, $CDCl_3$): δ 9.87 (s, 1H), 7.37 (d, *J* = 8.2 Hz, 2H), 7.19 (t, *J* = 8.0 Hz, 2H), 7.12 (d, *J* = 8.2 Hz, 2H), 6.69 (t, *J* = 7.3 Hz, 1H), 6.52 (d, *J* = 8.0 Hz, 2H), 6.37 (s, 1H), 4.79 (dd,

J = 8.2, 1.2 Hz, 1H), 3.79 – 3.72 (m, 1H), 3.44 (q, J = 8.7 Hz, 1H), 2.50 – 2.40 (m, 1H), 2.27 (s, 3H), 2.09 – 2.00 (m, 3H), 1.97 (s, 3H).

^{13}C NMR (125 MHz, CDCl$_3$): δ 185.4, 147.1, 145.8, 139.1, 135.5, 131.2, 129.2, 128.1, 127.2, 122.0, 116.3, 112.5, 105.8, 62.7, 49.3, 36.1, 23.3, 12.9, 11.5.

HRMS (ESI-TOF): Calculated for $C_{23}H_{25}N_2O^+$ ($[M + H]^+$): 345.1961, found 345.1965.

FTIR (thin film): 2919, 1659, 1598, 1506, 1425, 1362, 1162, 749, 693 cm^{-1}.

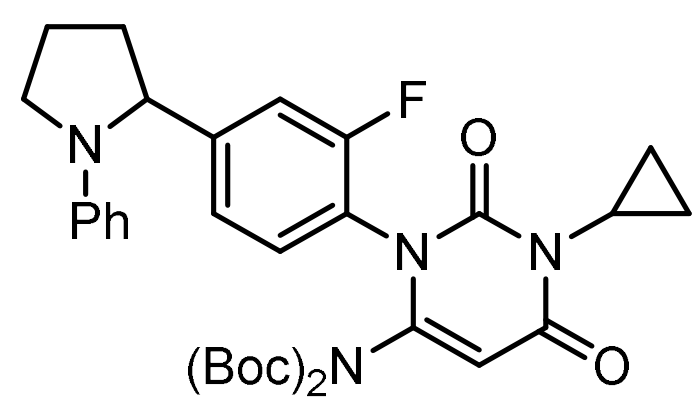

6-bis(*tert*-butoxycarbonyl)amino-3-cyclopropyl-1-(2-fluoro-4-(1-phenylpyrrolidin-2-yl)phenyl)pyrimidine-2,4(1*H*,3*H*)-dione (26). Prepared according to General Procedure using 6-bis(*tert*-butoxycarbonyl)-amino-3-cyclopropyl-1-(2-fluoro-4-iodophenyl)pyrimidine-2,4(1*H*,3*H*)-dione (235 mg, 0.40 mmol, 1.0 equiv) and *N*-phenylpyrrolidine (172 µL, 1.2 mmol, 3.0 equiv). The reaction was purified by preparative SFC using 15% *i*-PrOH/CO_2 as eluent (Chiralcel AD-H). The title compound was isolated as a brown solid (136 mg, 56% yield).

^{1}H NMR (500 MHz, CDCl$_3$): δ 7.32 – 7.23 (m, 1H), 7.14 (td, J = 8.3, 3.5 Hz, 2H), 7.11 – 7.02 (m, 2H), 6.67 (q, J = 7.1 Hz, 1H), 6.49 – 6.41 (m, 2H), 5.80 (d, J = 3.5 Hz, 1H), 4.70 (d, J = 8.4 Hz, 1H), 3.70 (t, J = 7.4 Hz, 1H), 3.39 (q, J = 8.7 Hz, 1H), 2.78 – 2.70 (m, 1H), 2.44 – 2.35 (m, 1H), 2.03 – 1.90 (m, 3H), 1.53 – 1.23 (m, 18H), 1.18 – 1.09 (m, 2H), 0.87 – 0.81 (m, 2H).

^{13}C NMR (125 MHz, CDCl$_3$): δ 163.4, [158.5 (d, J = 254.1 Hz), 158.4 (d, J = 254.1 Hz)], [151.6, 151.6], [149.8 (d, J = 1.8 Hz), 149.7 (d, J = 2.4 Hz)], [149.7, 149.5], [148.1, 147.9], 146.8, [146.6, 146.5], [129.8, 129.5], 129.3, [122.0 (d, J = 3.2 Hz), 121.8 (d, J = 3.1 Hz)], [120.8 (d, J = 4.5 Hz), 120.7 (d, J = 4.5 Hz)], [116.6, 116.6], [114.4 (d, J = 8.1 Hz), 114.2 (d, J = 8.0 Hz)], [112.5, 112.5], [103.3, 103.2], [85.5, 85.4], [85.4, 85.2], [62.5, 62.5], 49.3, 35.9, [28.0, 27.9], [27.6, 27.5], [25.5, 25.4], [23.2, 23.2], [8.7, 8.7], [8.7, 8.7]. [Conformational isomers denoted in brackets.]

^{19}F NMR (282 MHz, CDCl$_3$): δ –116.3, –116.6.

HRMS (ESI-TOF): Calculated for $C_{33}H_{40}FN_4O_6^+$ ($[M + H]^+$): 607.2926, found 607.2925.

FTIR (thin film): 2979, 1725, 1686, 1505, 1413, 1347, 1240, 1123, 751 cm^{-1}.

VII. Enantioselective Result

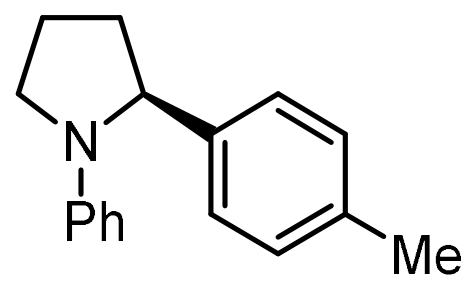

Optical rotation (30% ee): $[\alpha]_D^{20} = +39.1$ (*c* 1.0, $CHCl_3$).

Absolute configuration tentatively assigned as (*S*). (*S*)-1-(4-methoxyphenyl)-2,2-dimethyl-5-phenylpyrrolidine (88% ee): $[\alpha]_D^{20} = +16.3$ (*c* 6.5, $CHCl_3$).[12]

HPLC: Chiralcel OJ-H, Hexane:IPA = 95:5, 1 mL/min, 220 nm

Racemic Standard:

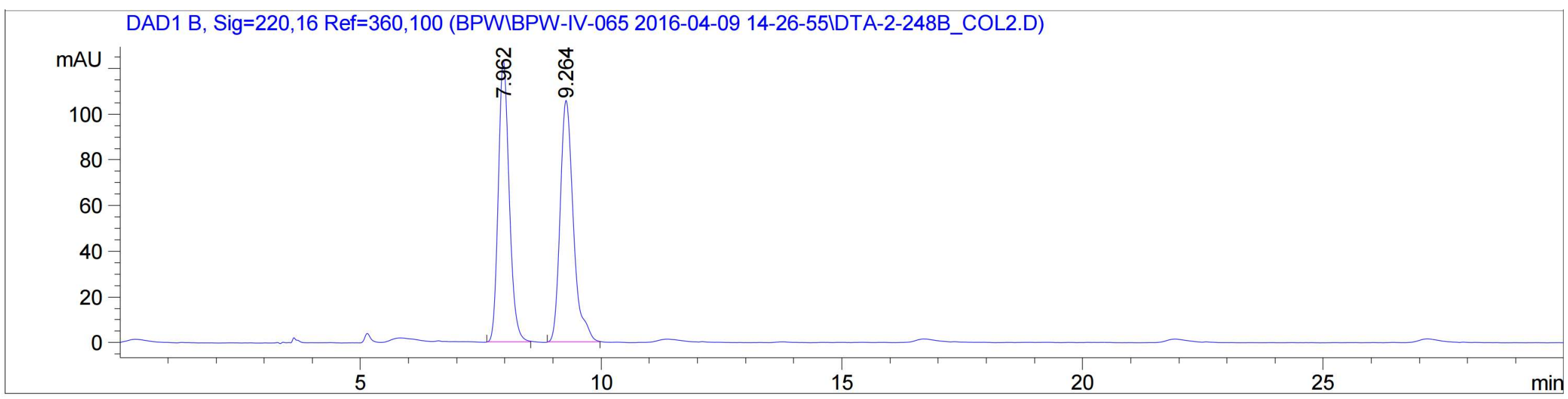

Peak #	RetTime [min]	Type	Width [min]	Area [mAU*s]	Height [mAU]	Area %
1	7.962	BB	0.2419	1939.45605	123.05058	48.9195
2	9.264	BB	0.2933	2025.12732	105.79005	51.0805

Enantioenriched:

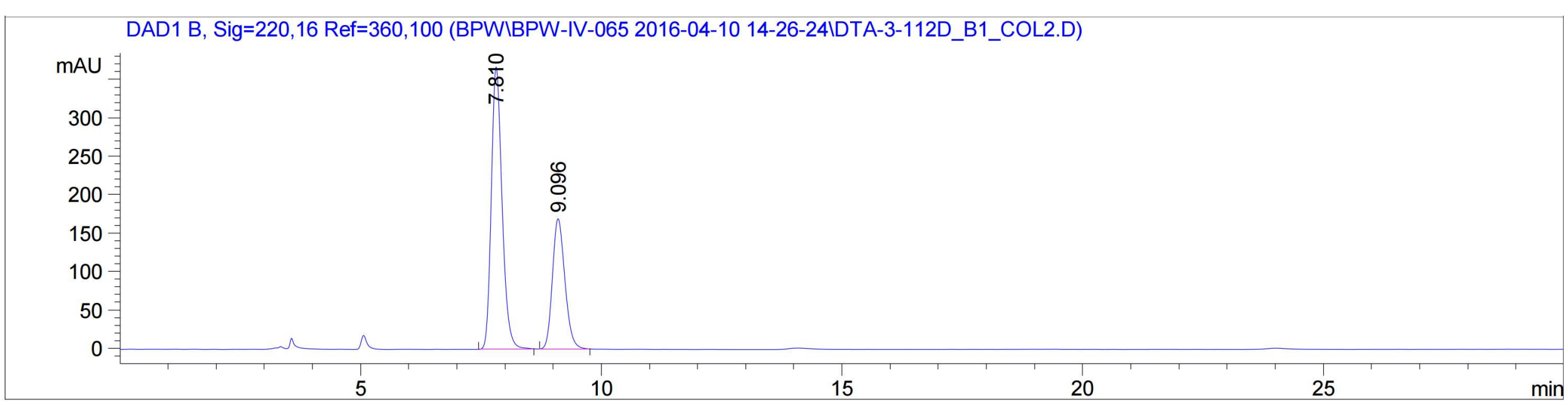

Peak #	RetTime [min]	Type	Width [min]	Area [mAU*s]	Height [mAU]	Area %
1	7.810	BB	0.2400	5730.46826	367.40759	65.1270
2	9.096	BB	0.2788	3068.44727	169.80104	34.8730

[12] I. Coldham, S. P. Robinson, C. A. Baxter, *Synlett* **2012**, *23*, 2405–2407.

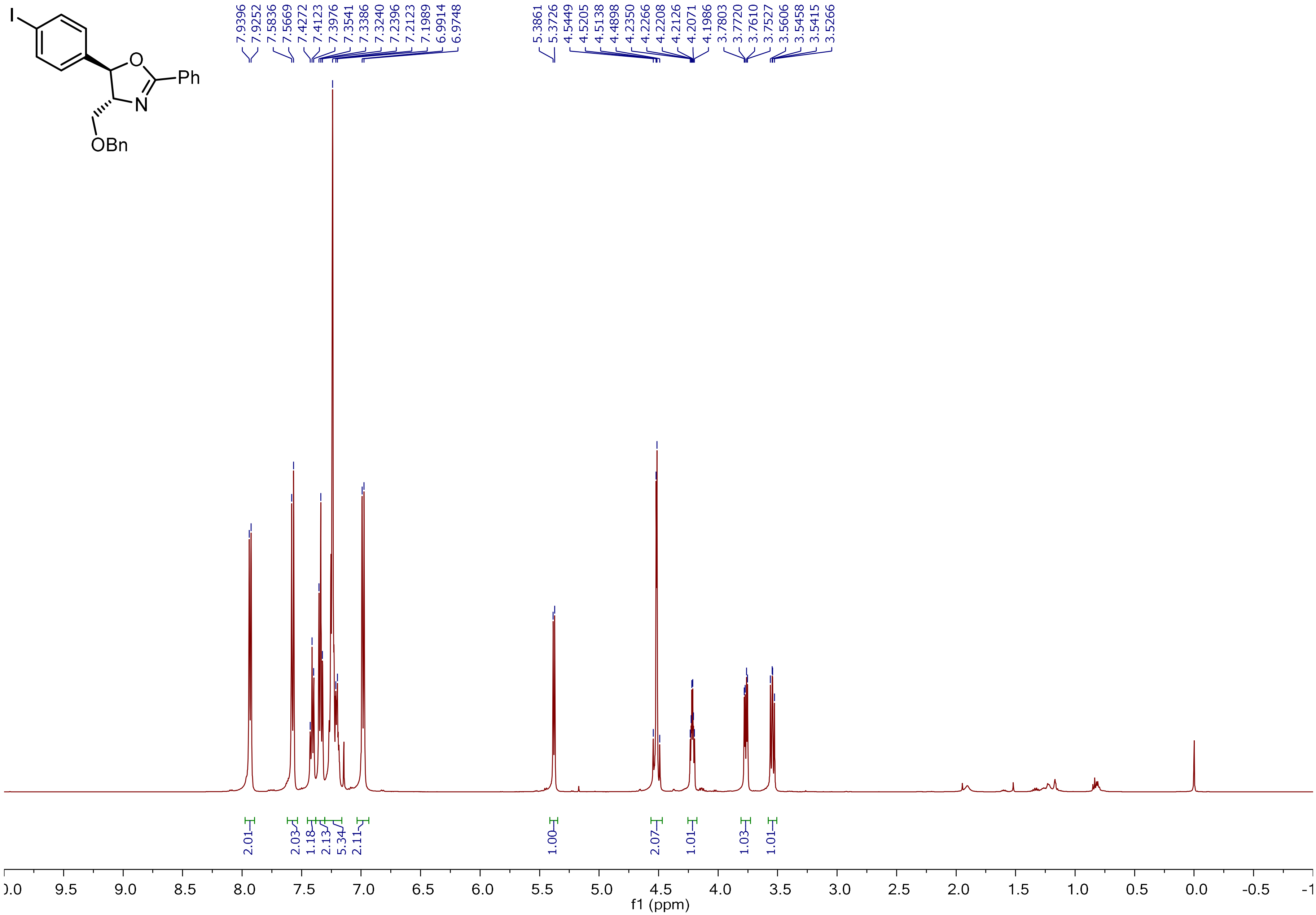

I
O
N
Ph
OBn
7.9396
7.9252
7.5836
7.5669
7.4272
7.4123
7.3976
7.3541
7.3386
7.3240
7.2396
7.2123
7.1989
6.9914
6.9748
5.3861
5.3726
4.5449
4.5205
4.5138
4.4898
4.2350
4.2266
4.2208
4.2126
4.2071
4.1986
3.7803
3.7720
3.7610
3.7527
3.5606
3.5458
3.5415
3.5266
2.01
2.03
1.18
2.13
5.34
2.11
1.00
2.07
1.01
1.03
1.01
9.5
9.0
8.5
8.0
7.5
7.0
6.5
6.0
5.5
5.0
4.5
4.0
3.5
3.0
2.5
2.0
1.5
1.0
0.5
0.0
-0.5
f1 (ppm)

^{13}C NMR (125 MHz, $CDCl_3$): (4*R*,5*R*)-4-((benzyloxy)methyl)-5-(4-iodophenyl)-2-phenyl-4,5-dihydrooxazole (SI-1)

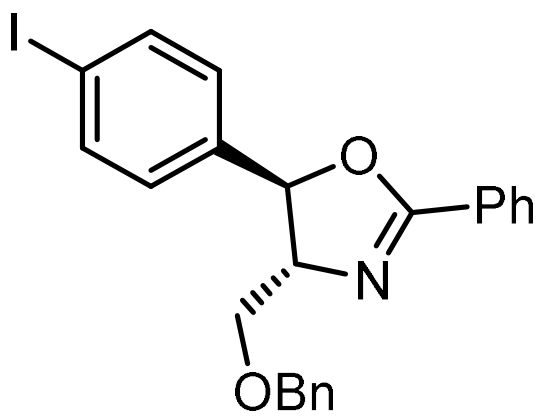

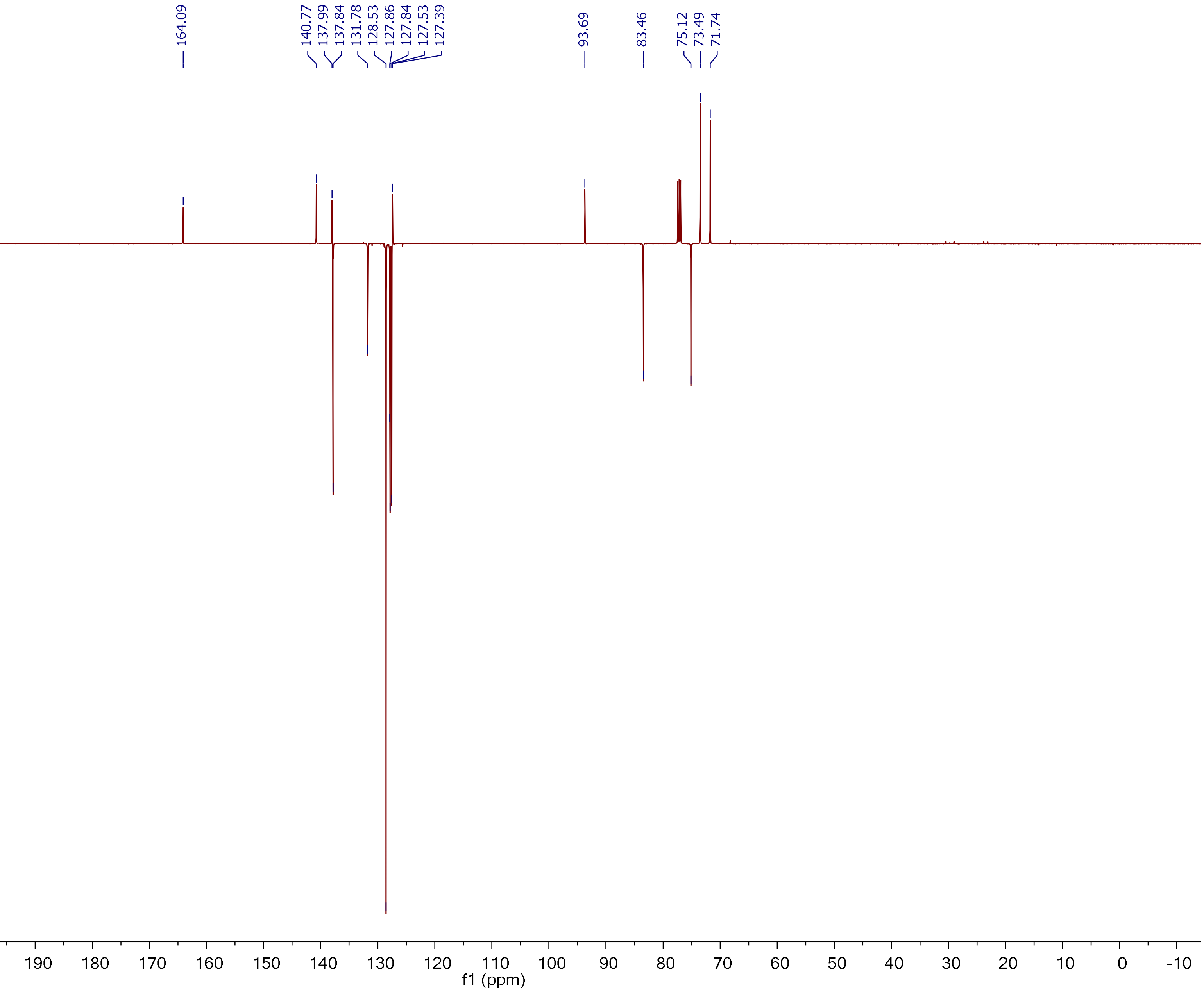

^{1}H NMR (500 MHz, $CDCl_3$): 6-bis(*tert*-butoxycarbonyl)amino-3-cyclopropyl-1-(2-fluoro-4-iodophenyl)pyrimidine-2,4(1*H*,3*H*)-dione (SI-2)

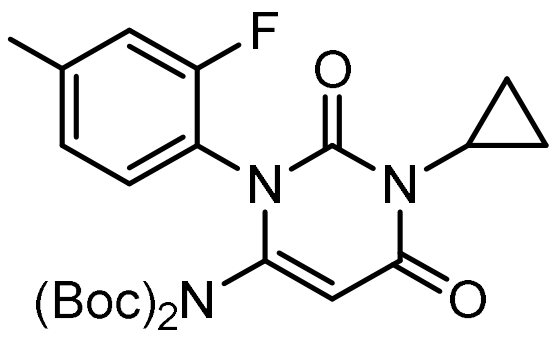

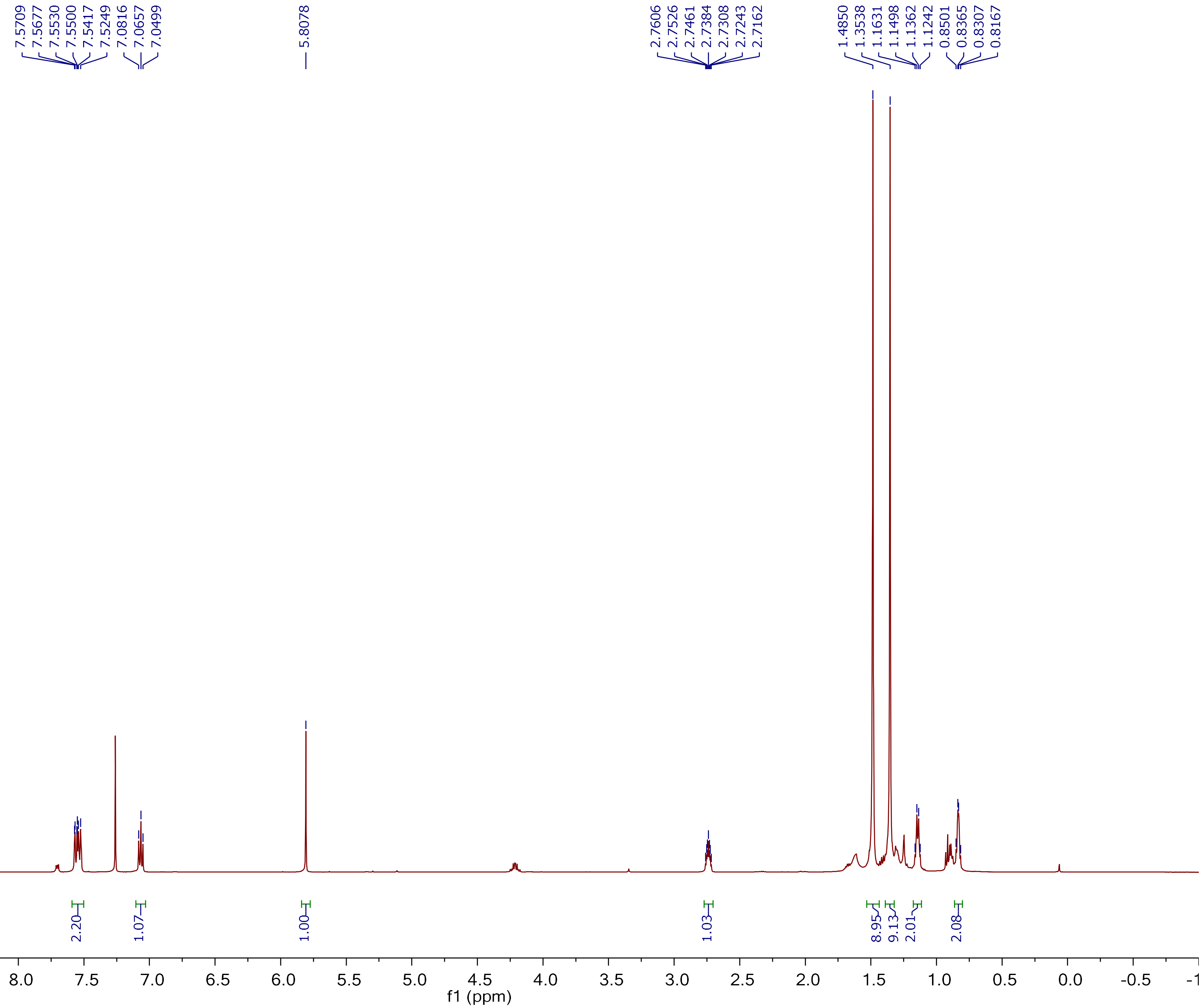

^{13}C NMR (125 MHz, CDCl$_3$): 6-bis(*tert*-butoxycarbonyl)amino-3-cyclopropyl-1-(2-fluoro-4-iodophenyl)pyrimidine-2,4(1*H*,3*H*)-dione (SI-2)

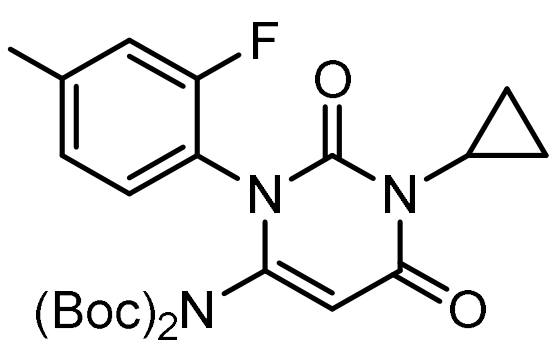

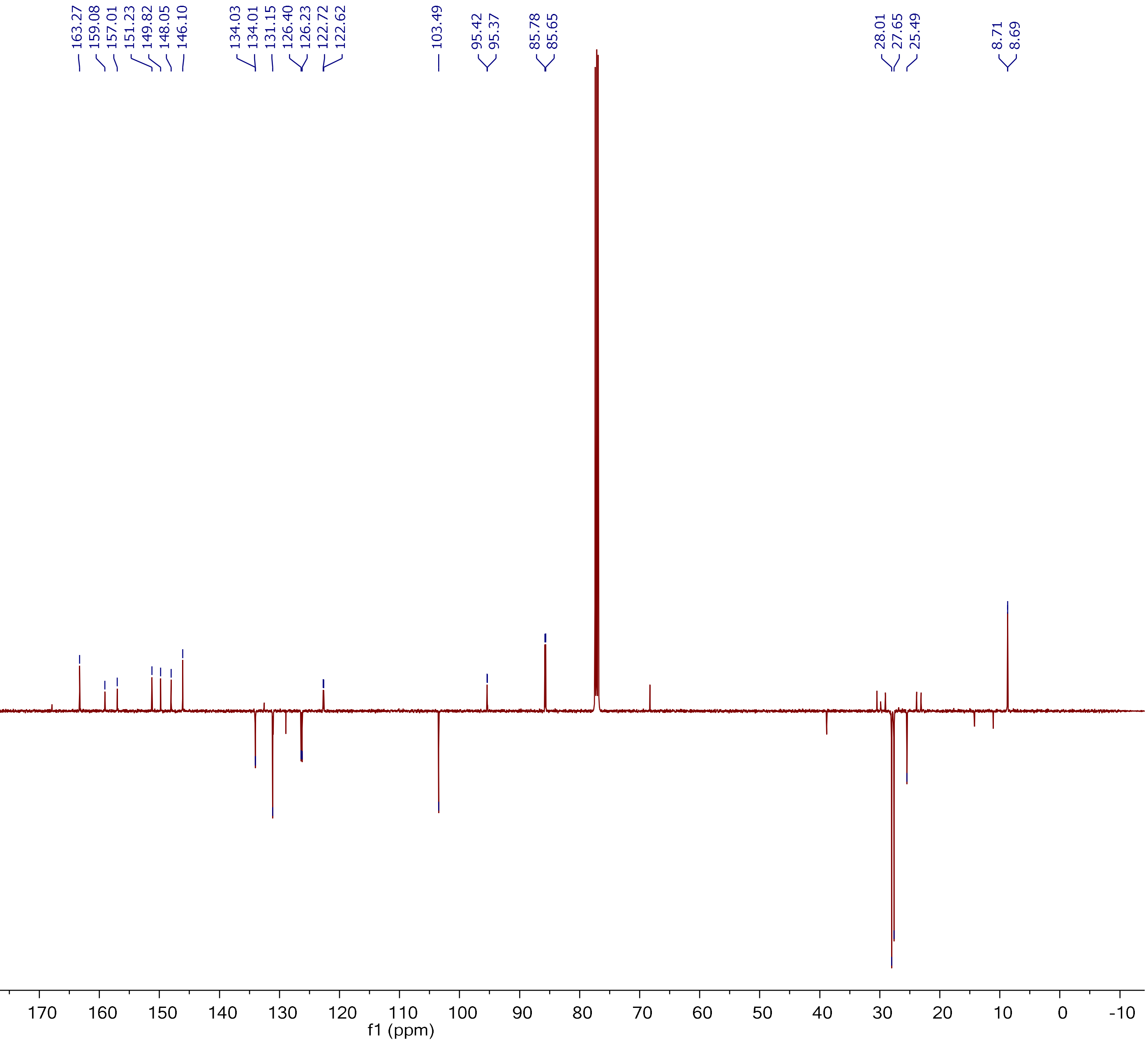

^{19}F NMR (282 MHz, $CDCl_3$): 6-bis(*tert*-butoxycarbonyl)amino-3-cyclopropyl-1-(2-fluoro-4-iodophenyl)pyrimidine-2,4(1*H*,3*H*)-dione (SI-2)

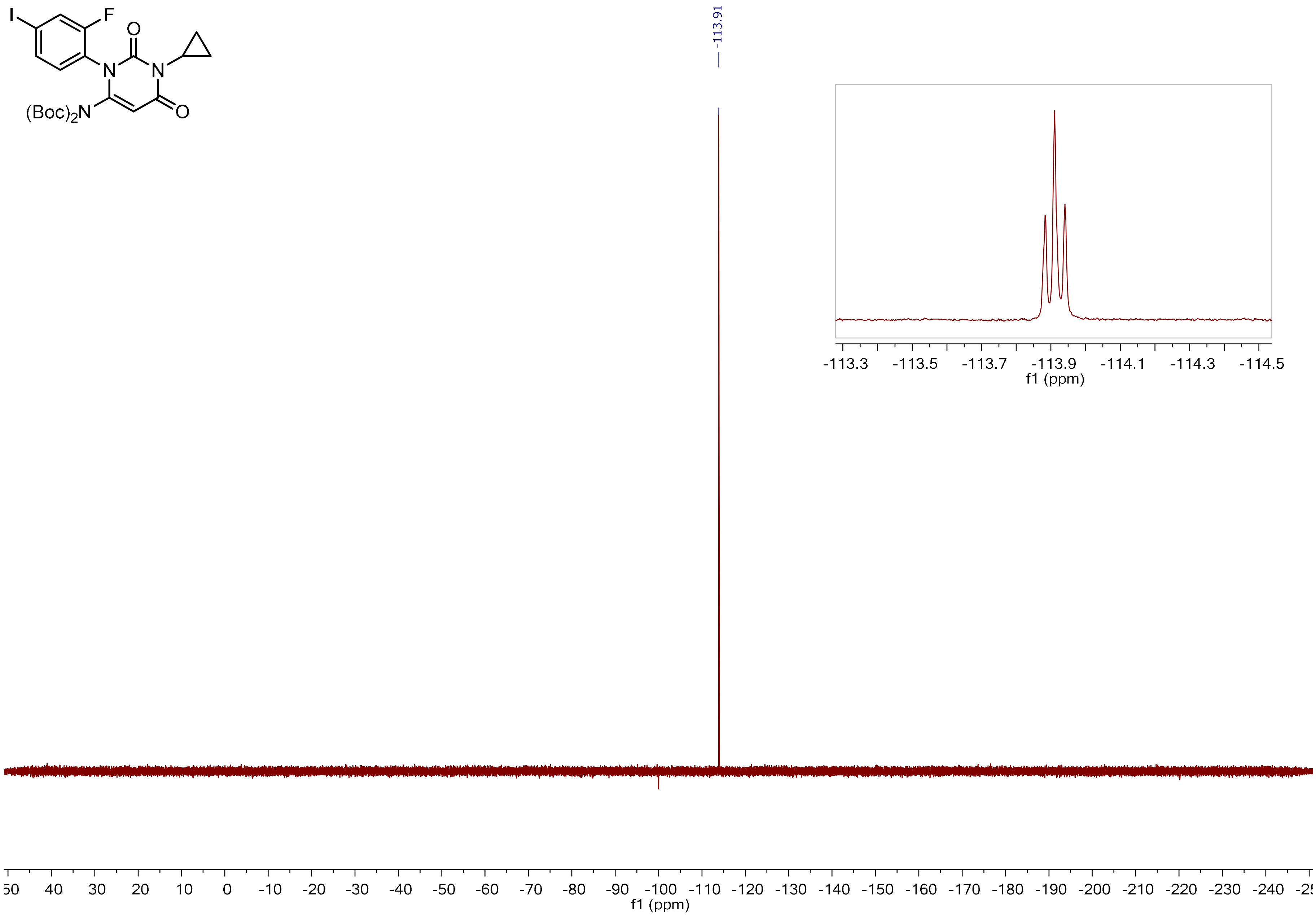

HSQC NMR ($CDCl_3$): 6-bis(*tert*-butoxycarbonyl)amino-3-cyclopropyl-1-(2-fluoro-4-iodophenyl)pyrimidine-2,4(1*H*,3*H*)-dione (SI-2)

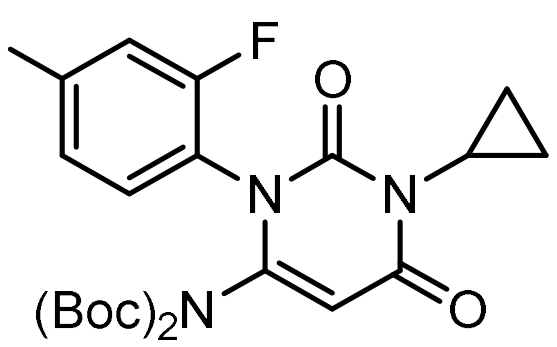

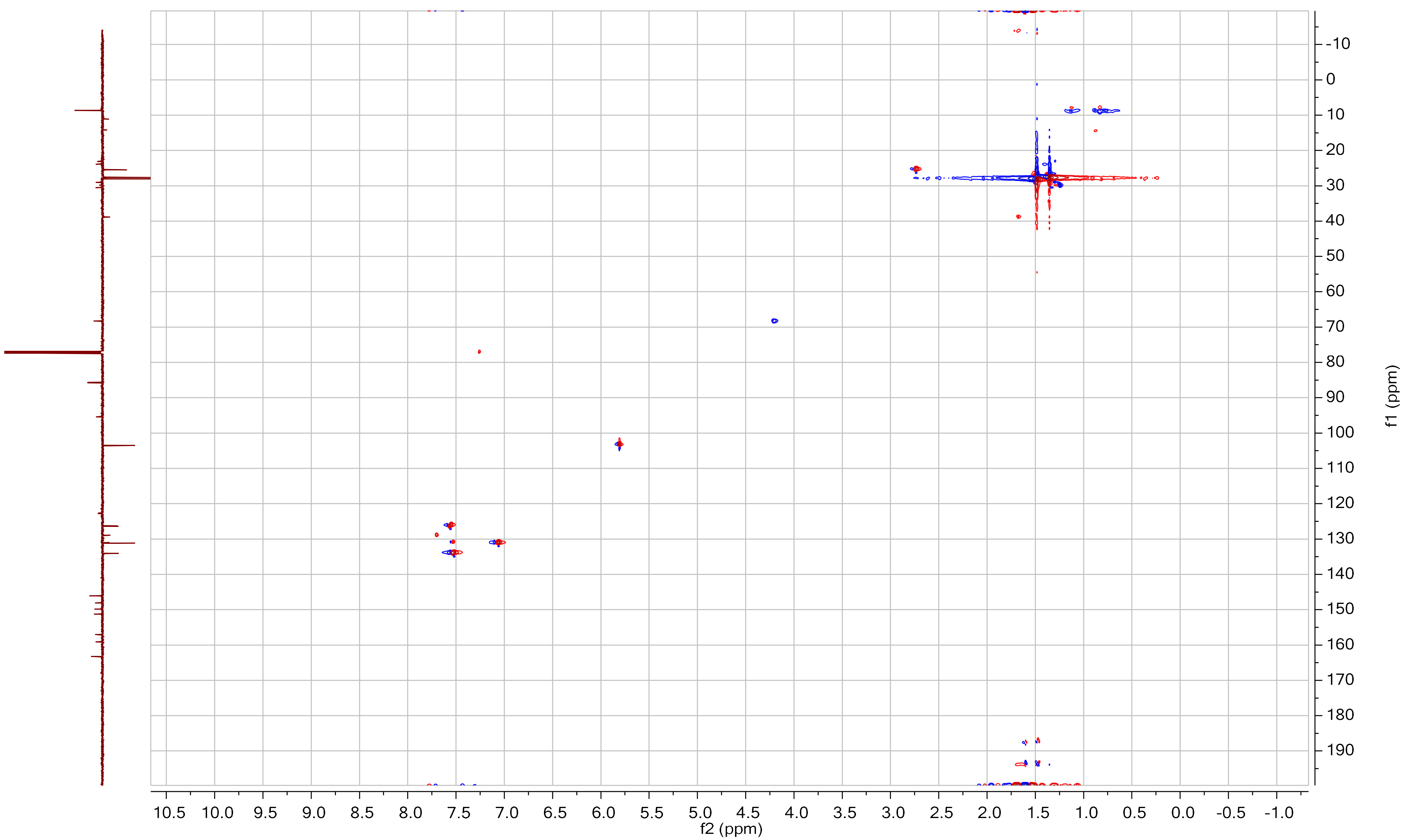

HMBC NMR ($CDCl_3$): 6-bis(*tert*-butoxycarbonyl)amino-3-cyclopropyl-1-(2-fluoro-4-iodophenyl)pyrimidine-2,4(1*H*,3*H*)-dione (SI-2)

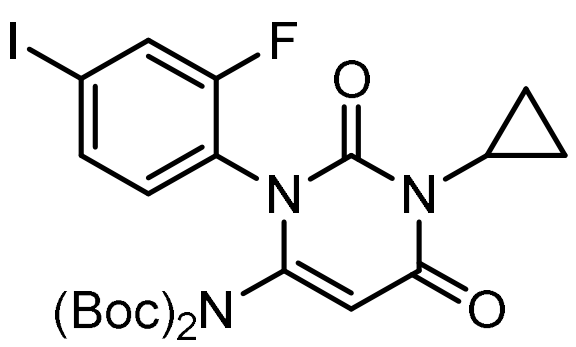

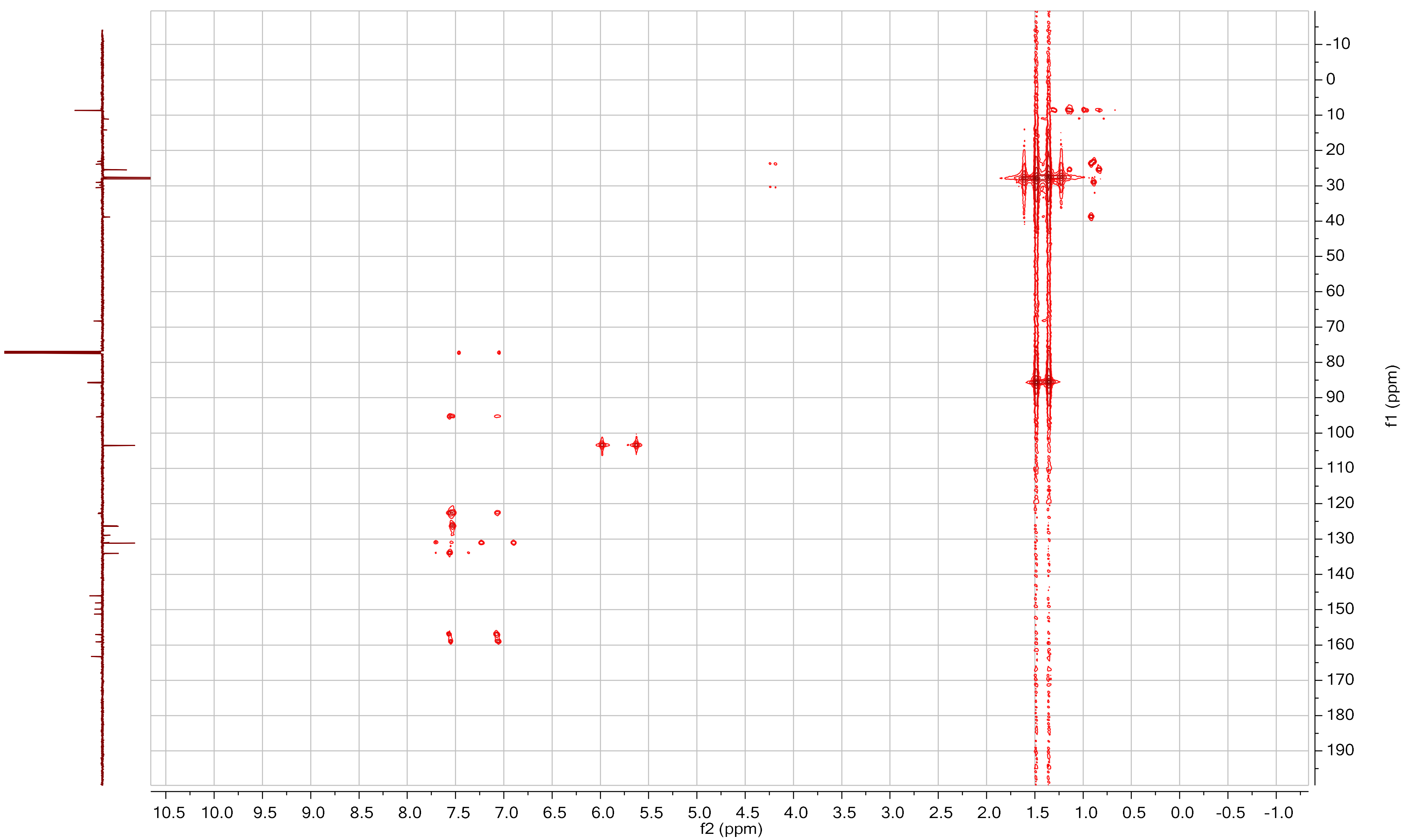

^{1}H NMR (500 MHz, $CDCl_3$): 1-phenyl-2-(*p*-tolyl)pyrrolidine (1)

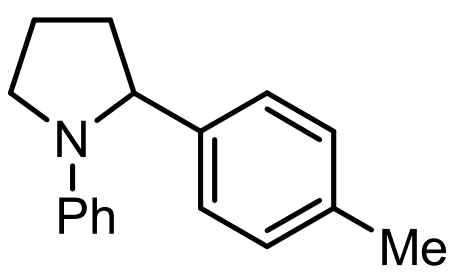

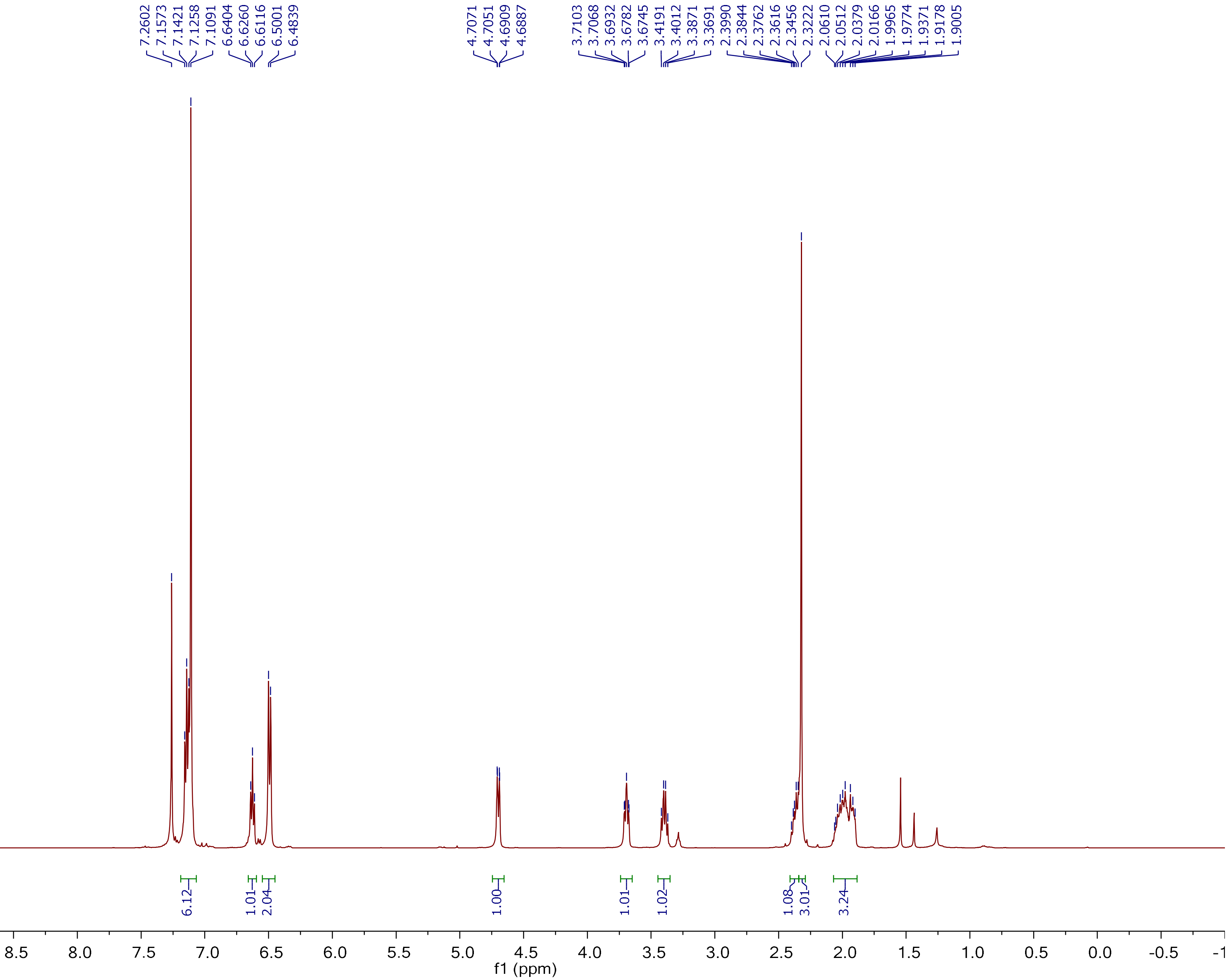

^{13}C NMR (125 MHz, $CDCl_3$): 1-phenyl-2-(*p*-tolyl)pyrrolidine (1)

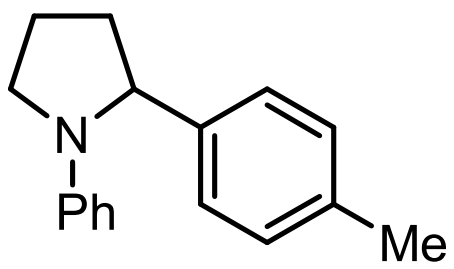

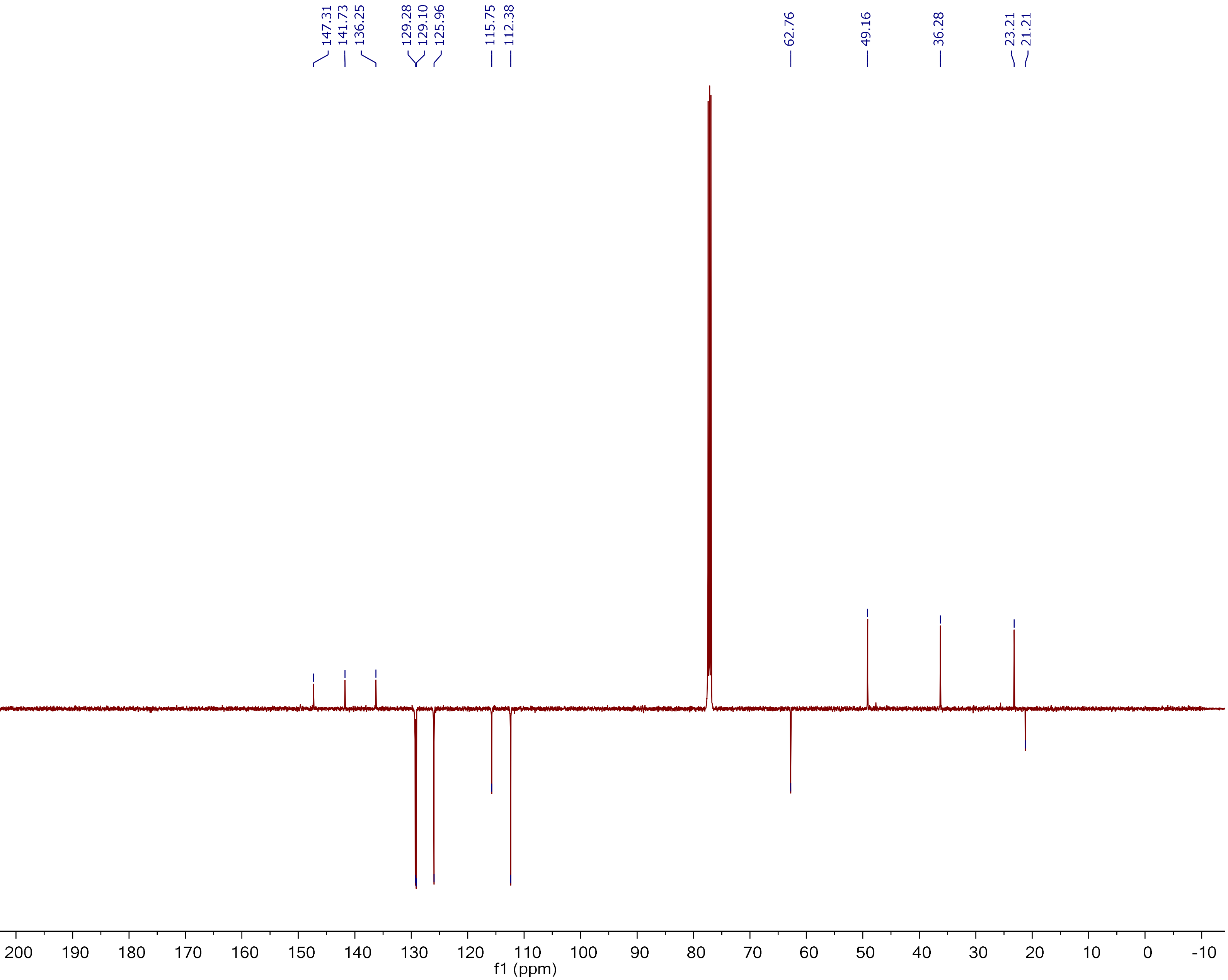

^{1}H NMR (500 MHz, $CDCl_3$): 1-phenyl-2-(*m*-tolyl)pyrrolidine (2)

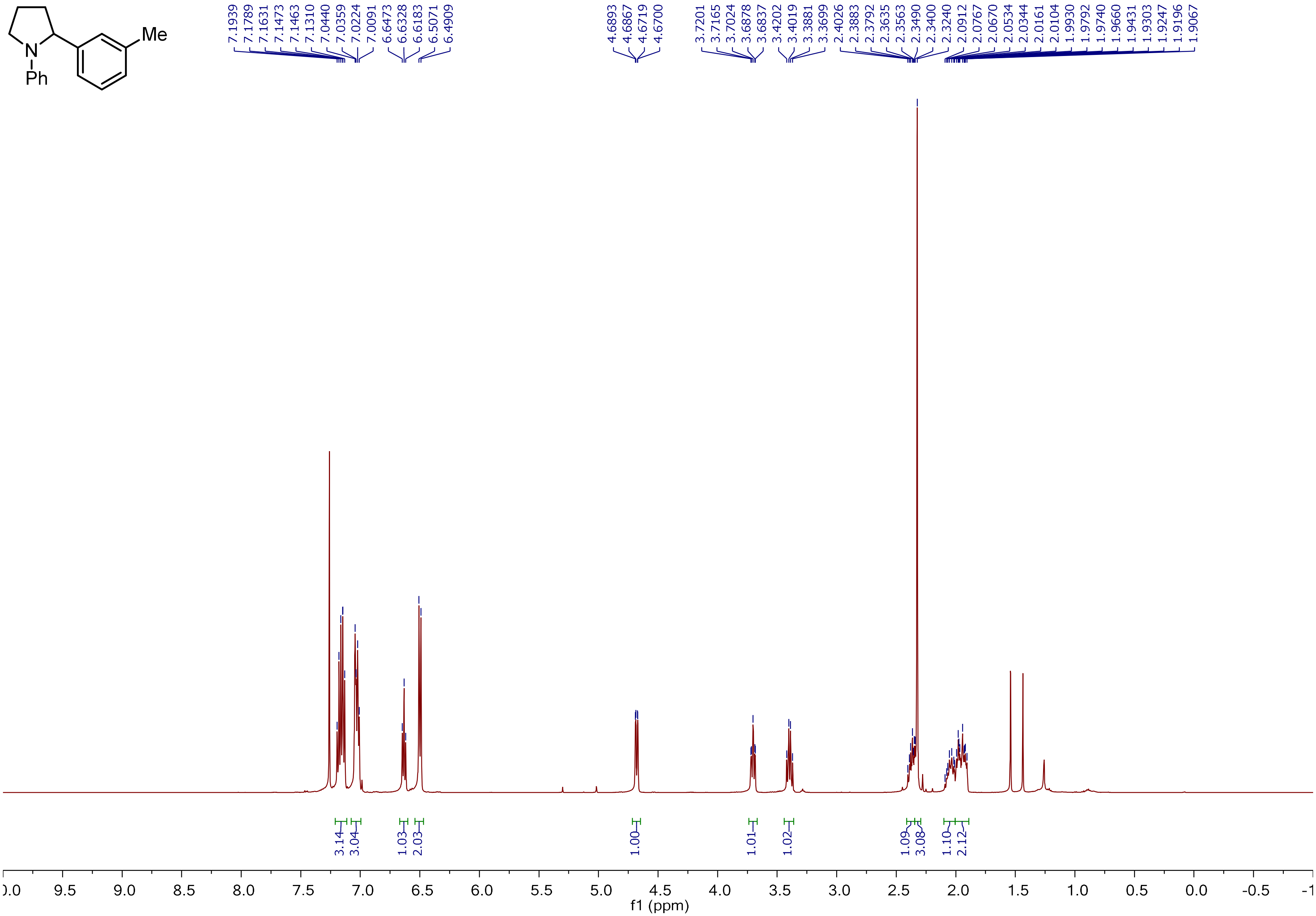

^{13}C NMR (125 MHz, $CDCl_3$): 1-phenyl-2-(*m*-tolyl)pyrrolidine (2)

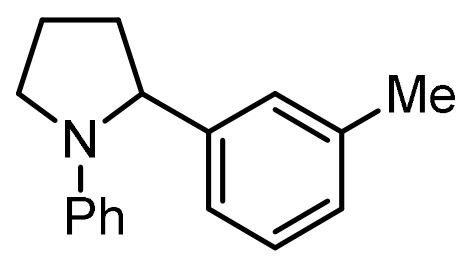

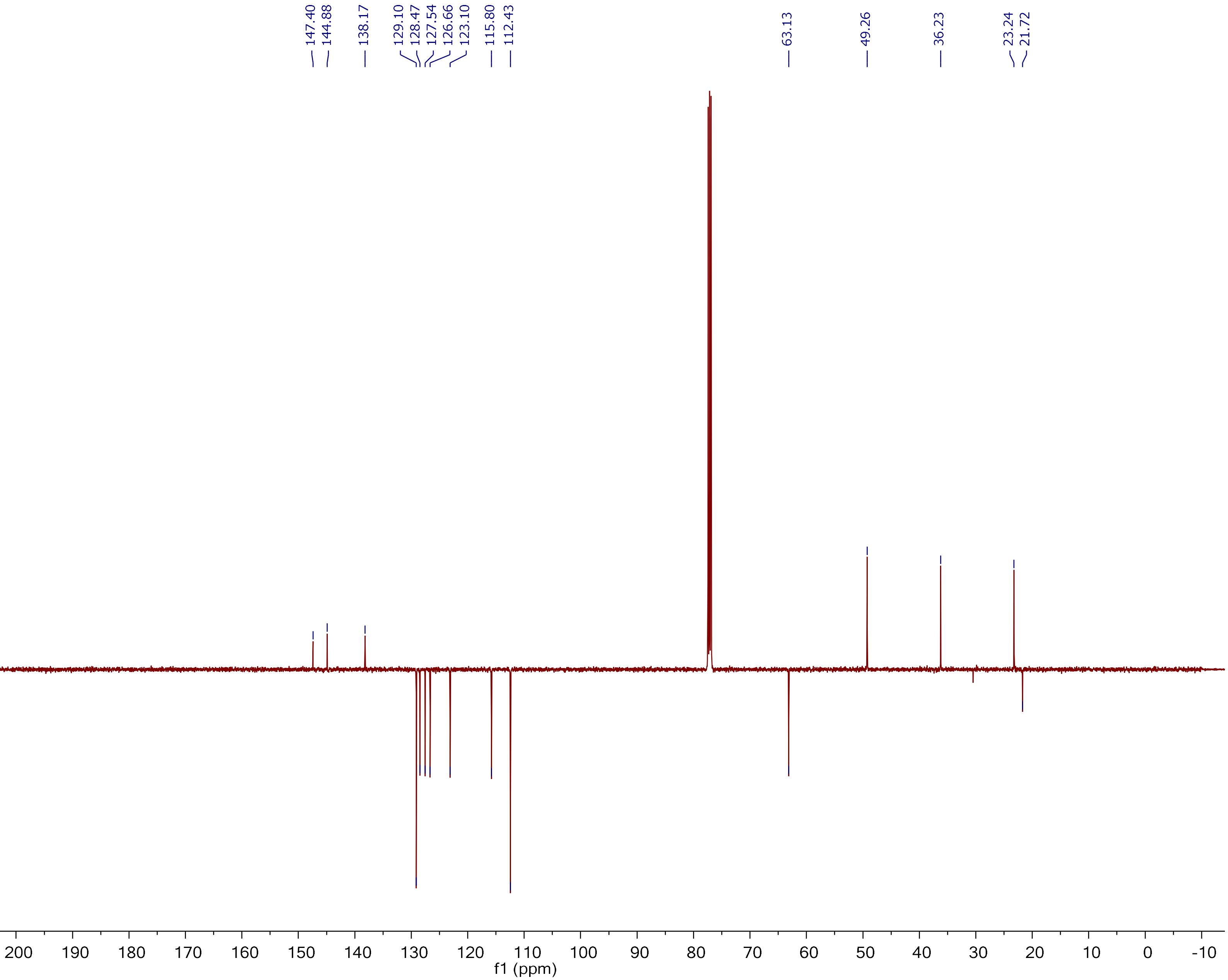

^{1}H NMR (500 MHz, $CDCl_3$): 1-phenyl-2-(*o*-tolyl)pyrrolidine (3)

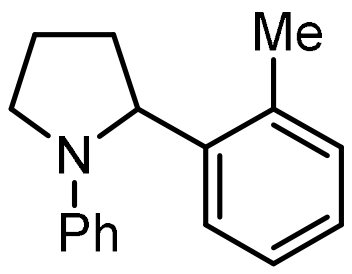

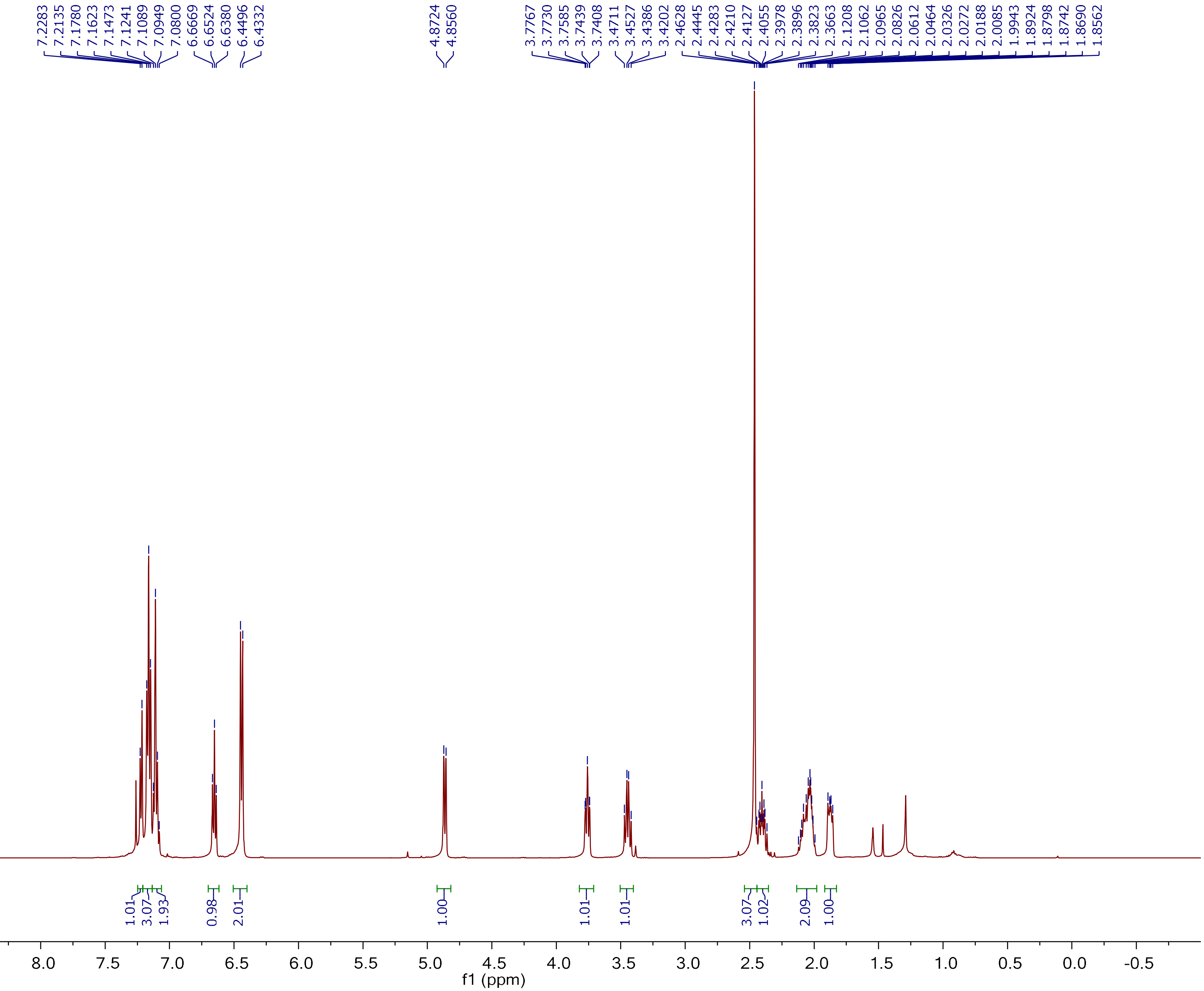

^{13}C NMR (125 MHz, $CDCl_3$): 1-phenyl-2-(*o*-tolyl)pyrrolidine (3)

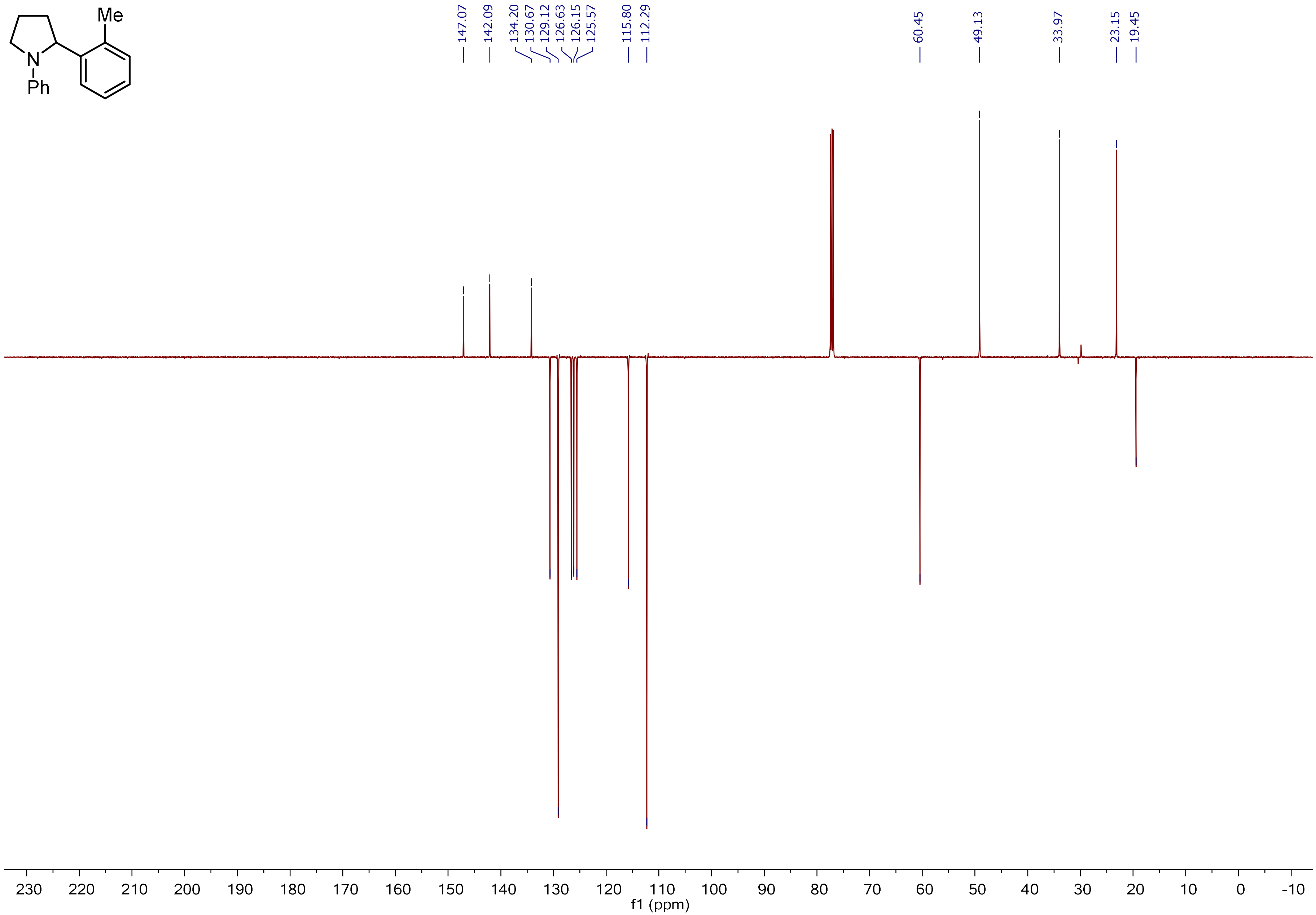

^{1}H NMR (500 MHz, $CDCl_3$): *N*,*N*-diethyl-4-(1-phenylpyrrolidin-2-yl)benzamide (4)

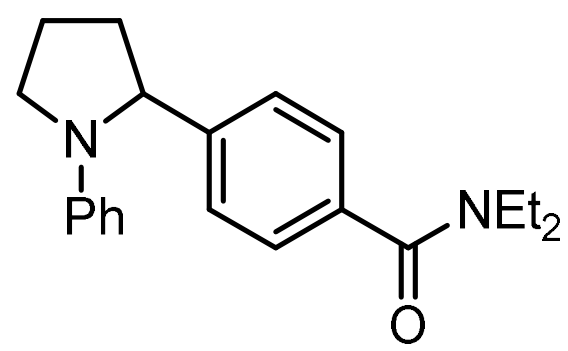

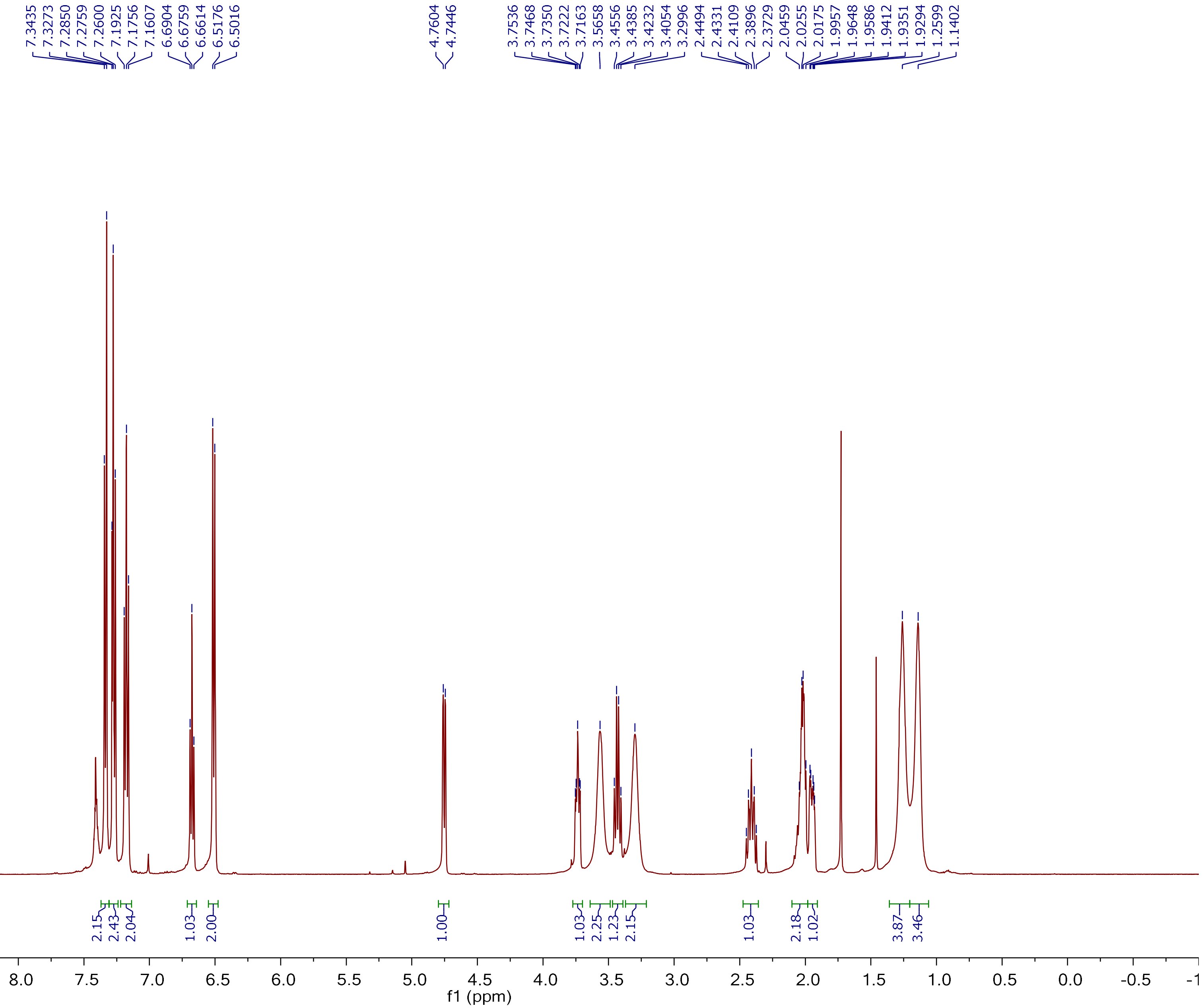

^{13}C NMR (125 MHz, $CDCl_3$): *N,N*-diethyl-4-(1-phenylpyrrolidin-2-yl)benzamide (4)

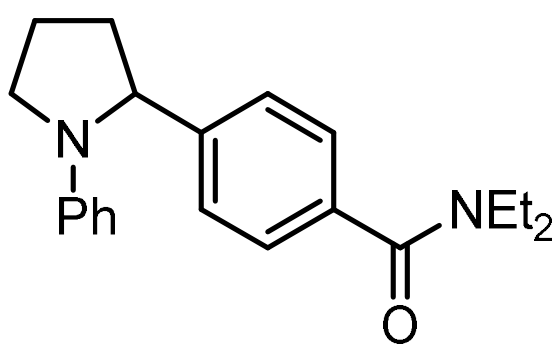

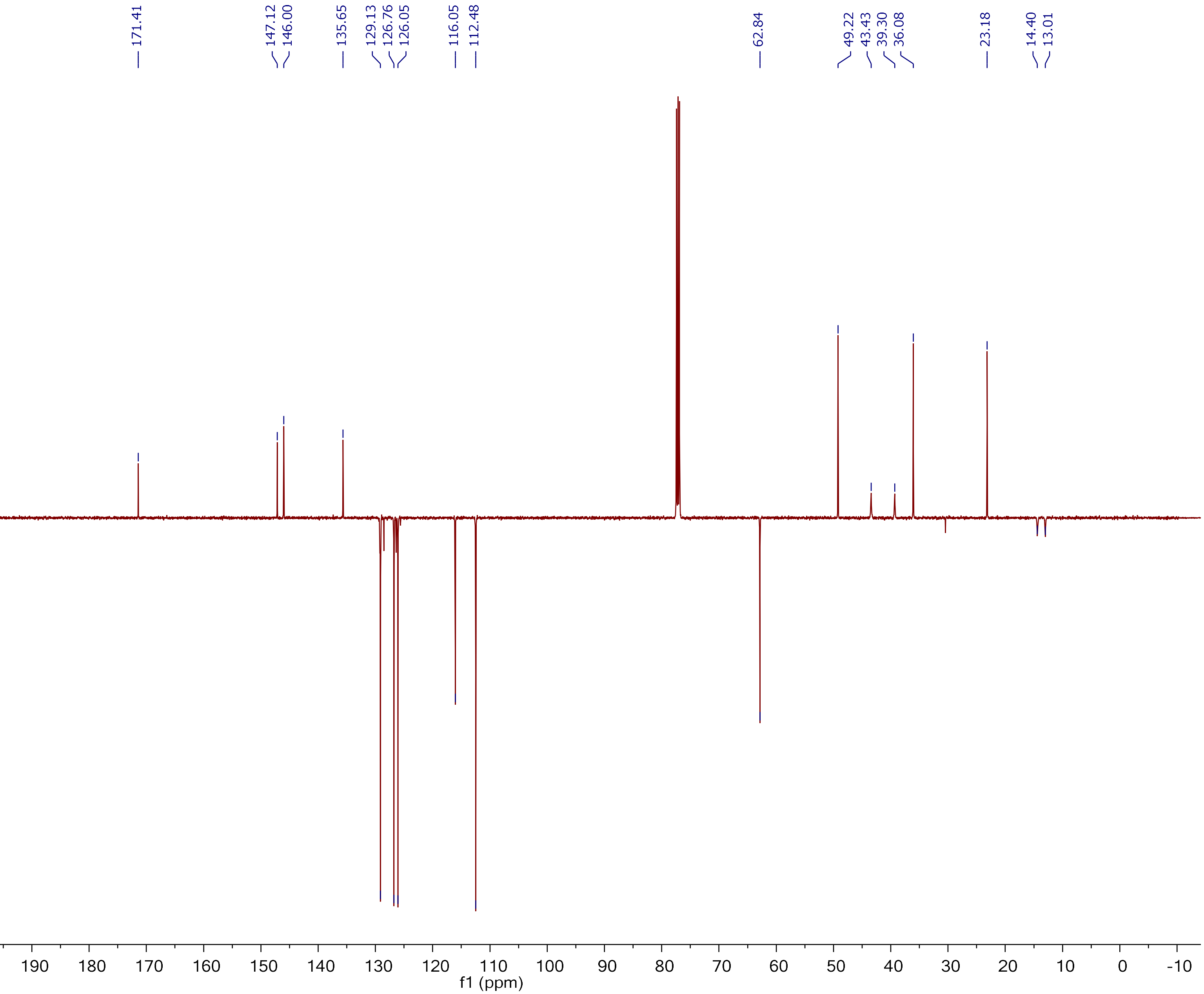

^{1}H NMR (500 MHz, $CDCl_3$): 2-(4-fluorophenyl)-1-phenylpyrrolidine (5)

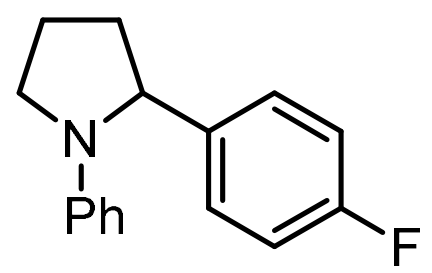

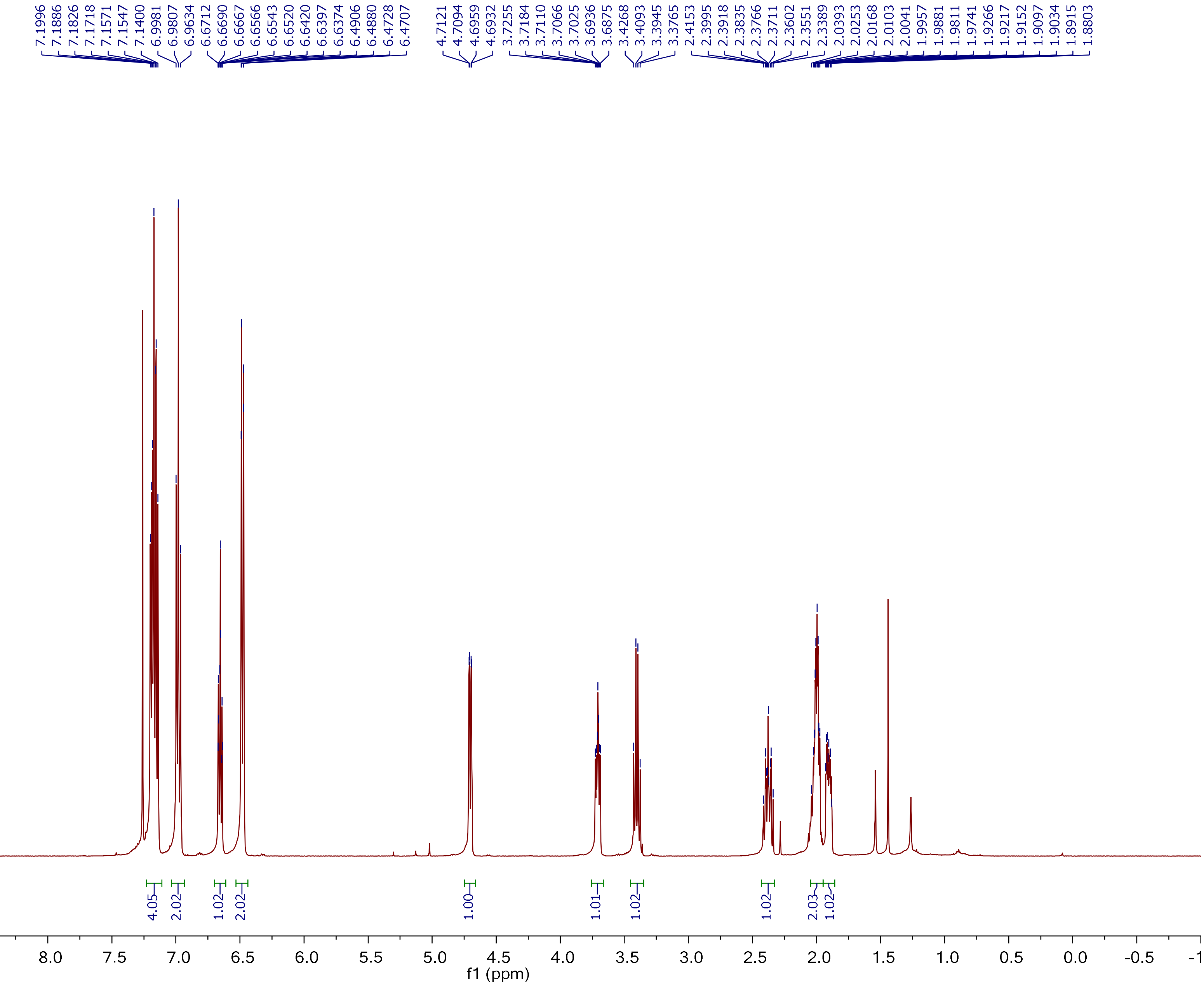

^{13}C NMR (125 MHz, $CDCl_3$): 2-(4-fluorophenyl)-1-phenylpyrrolidine (5)

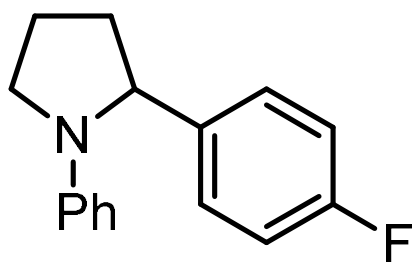

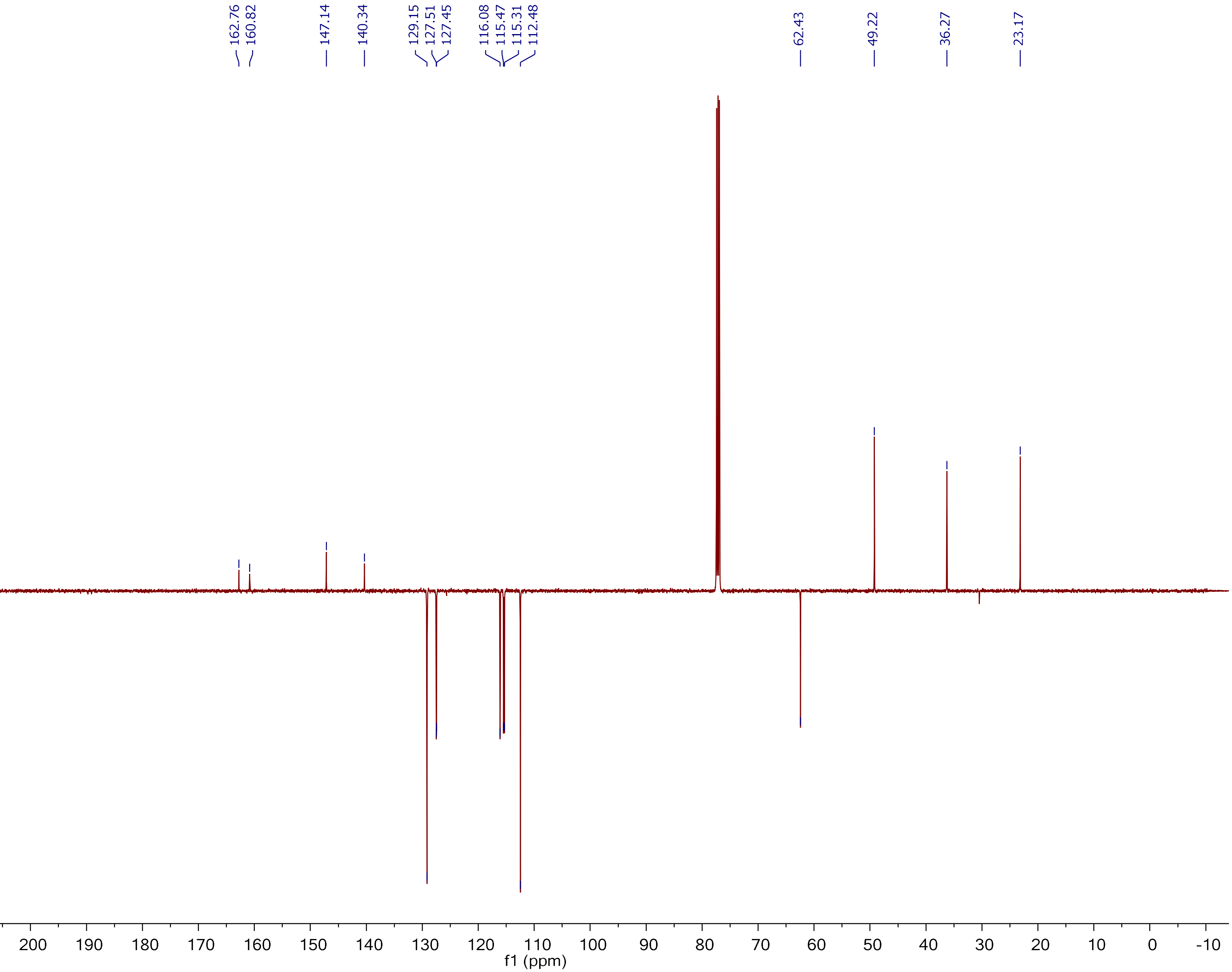

^{19}F NMR (282 MHz, $CDCl_3$): 2-(4-fluorophenyl)-1-phenylpyrrolidine (5)

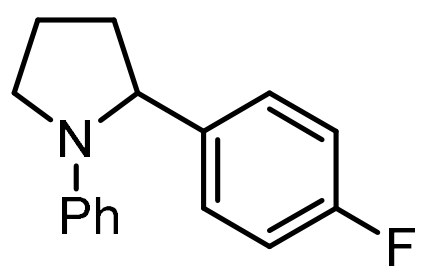

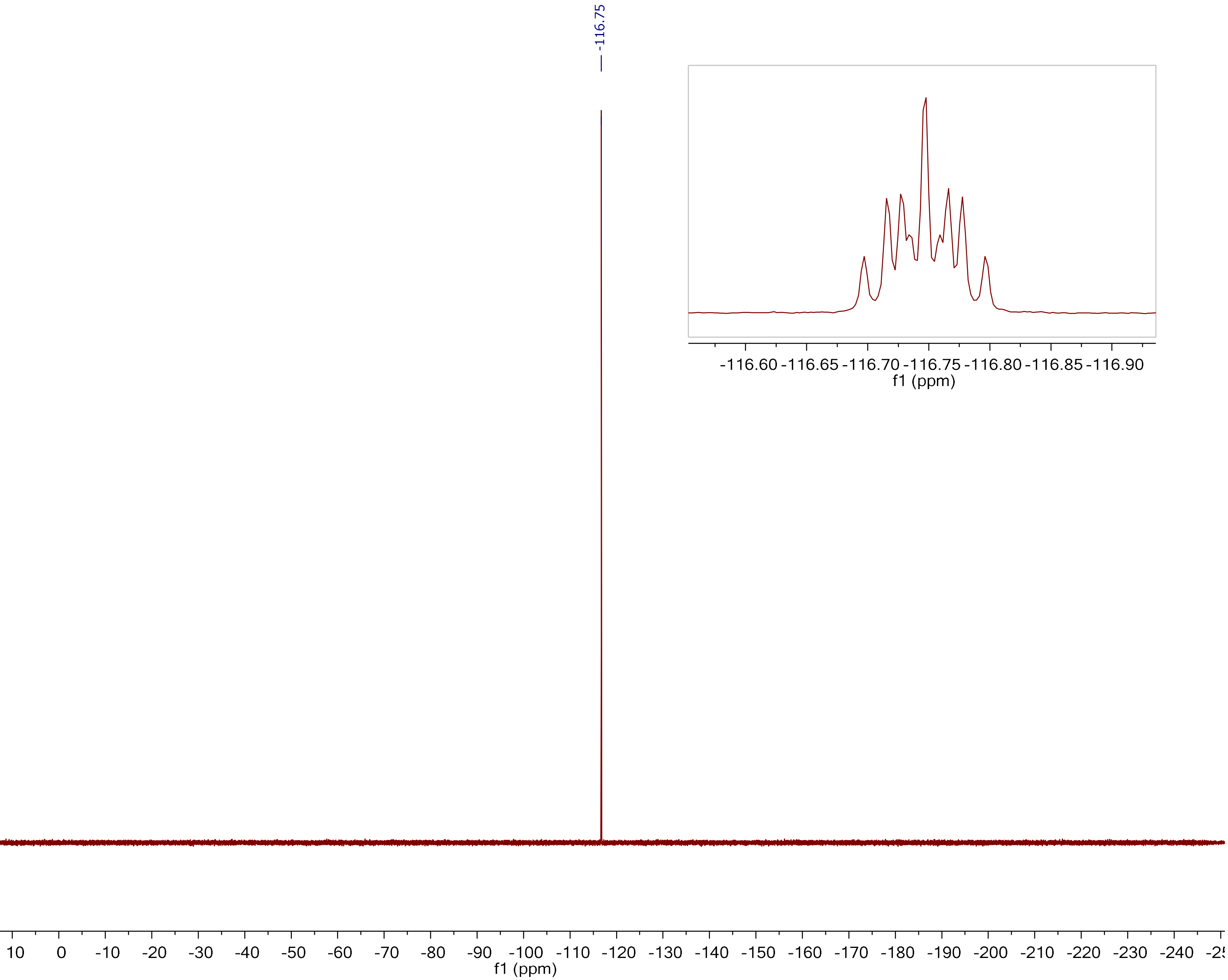

^{1}H NMR (500 MHz, $CDCl_3$): 1-phenyl-2-(4-(trifluoromethyl)phenyl)pyrrolidine (6)

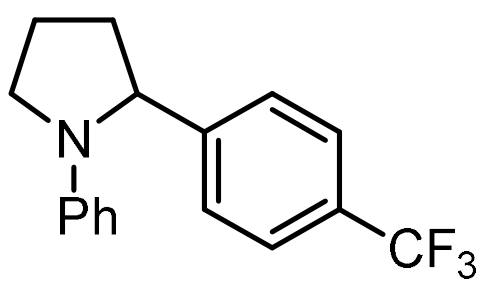

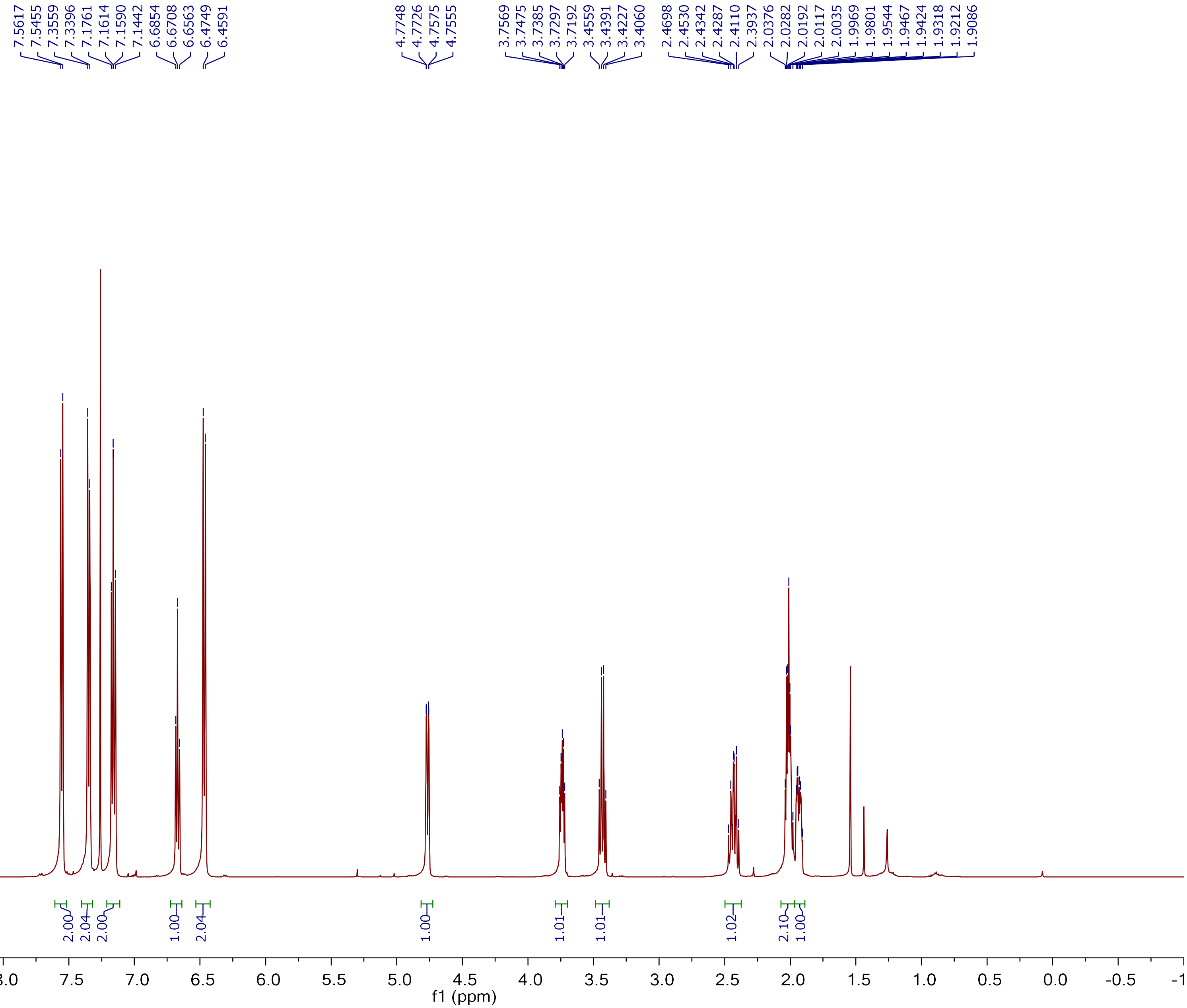

^{13}C NMR (125 MHz, $CDCl_3$): 1-phenyl-2-(4-(trifluoromethyl)phenyl)pyrrolidine (6)

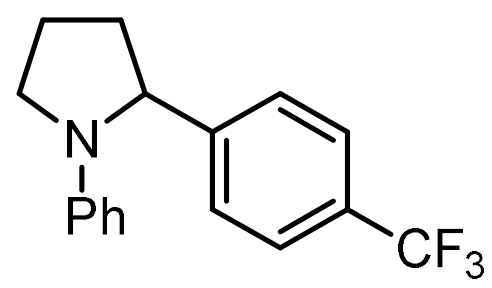

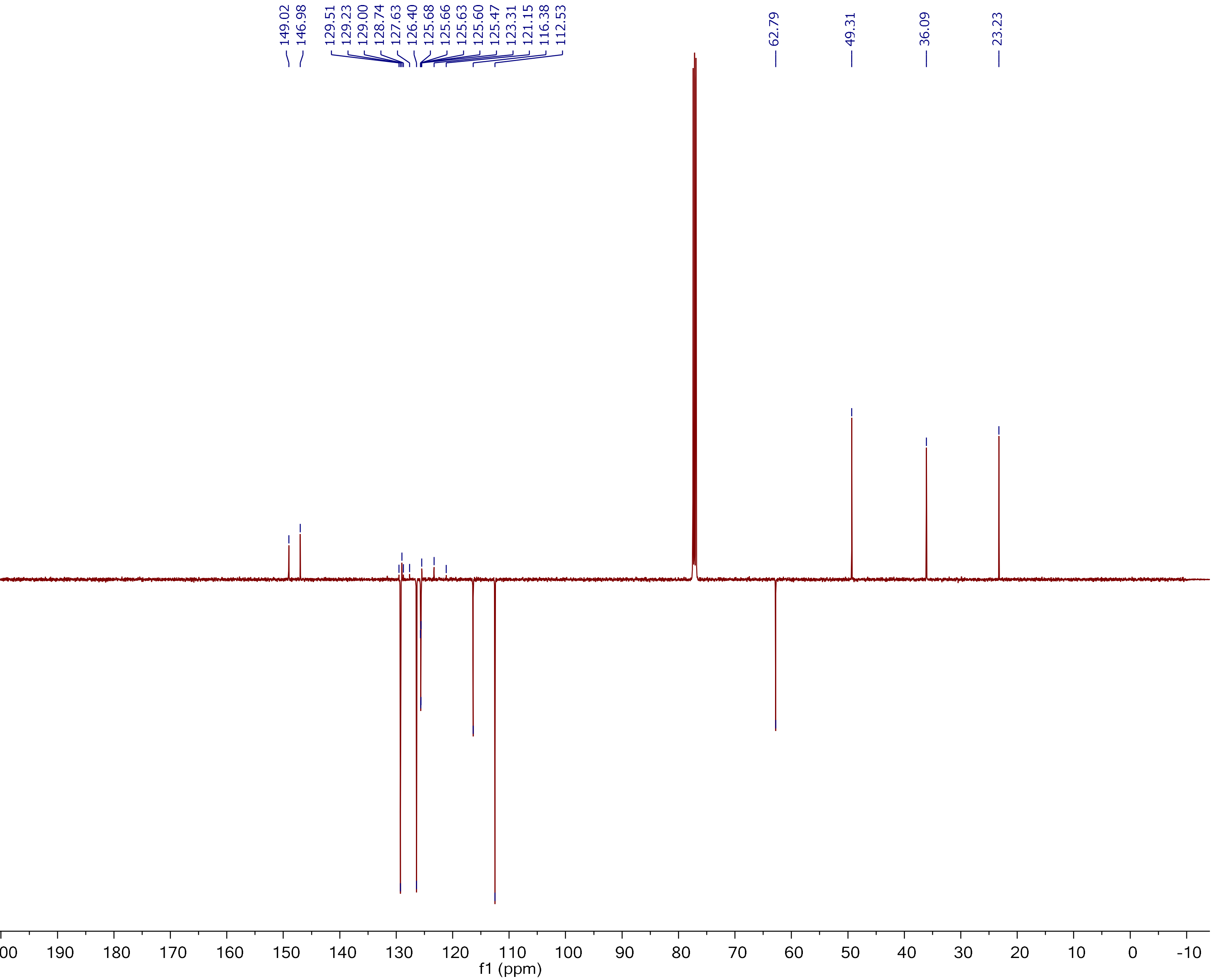

^{19}F NMR (282 MHz, $CDCl_3$): 1-phenyl-2-(4-(trifluoromethyl)phenyl)pyrrolidine (6)

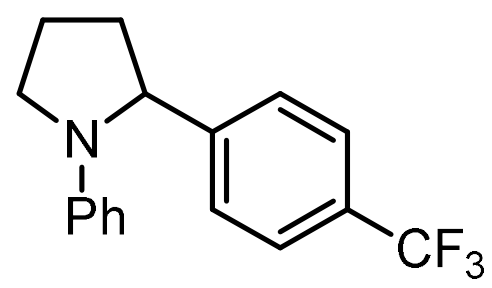

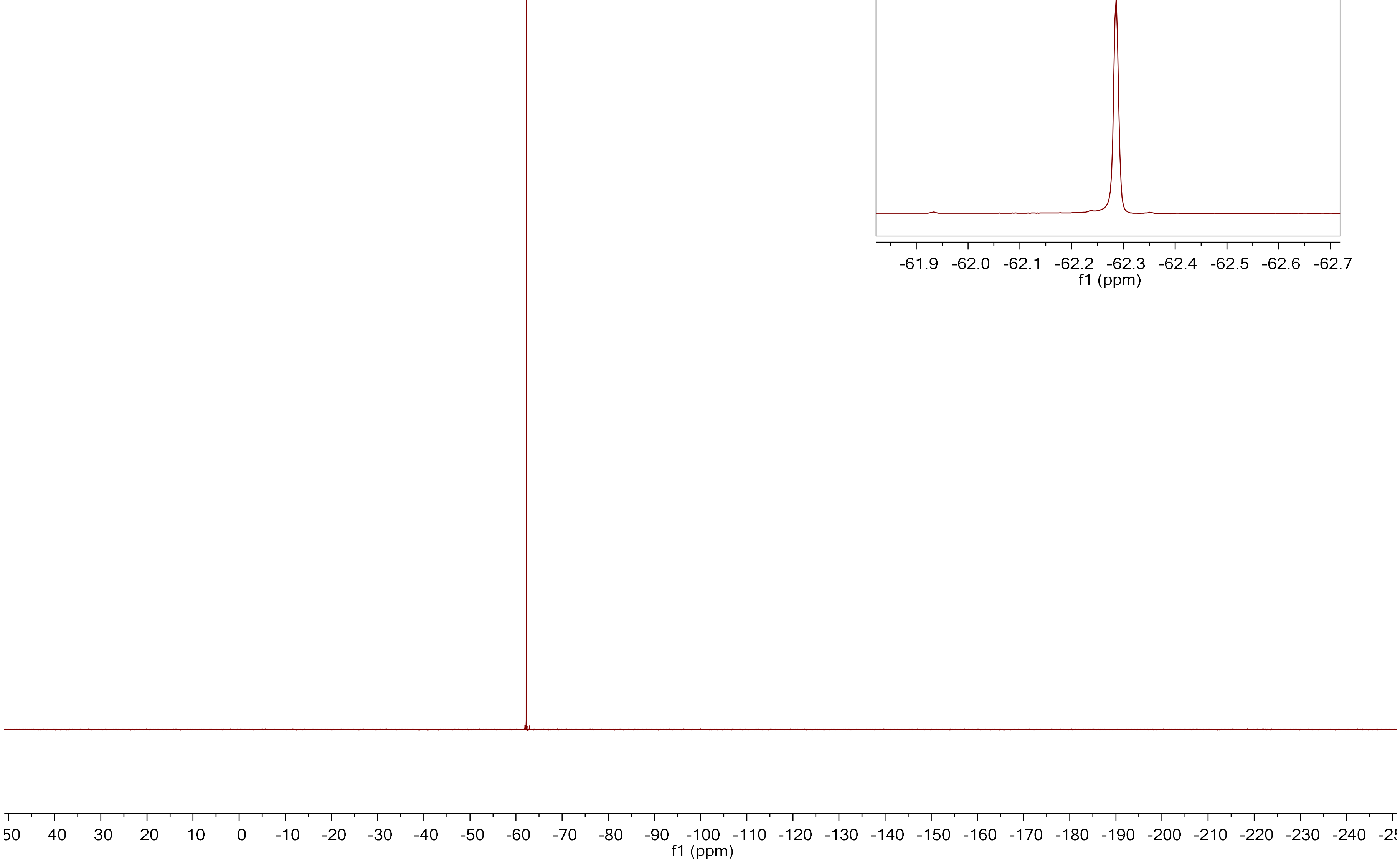

^{1}H NMR (500 MHz, $CDCl_3$): 2-(4-methoxyphenyl)-1-phenylpyrrolidine (7)

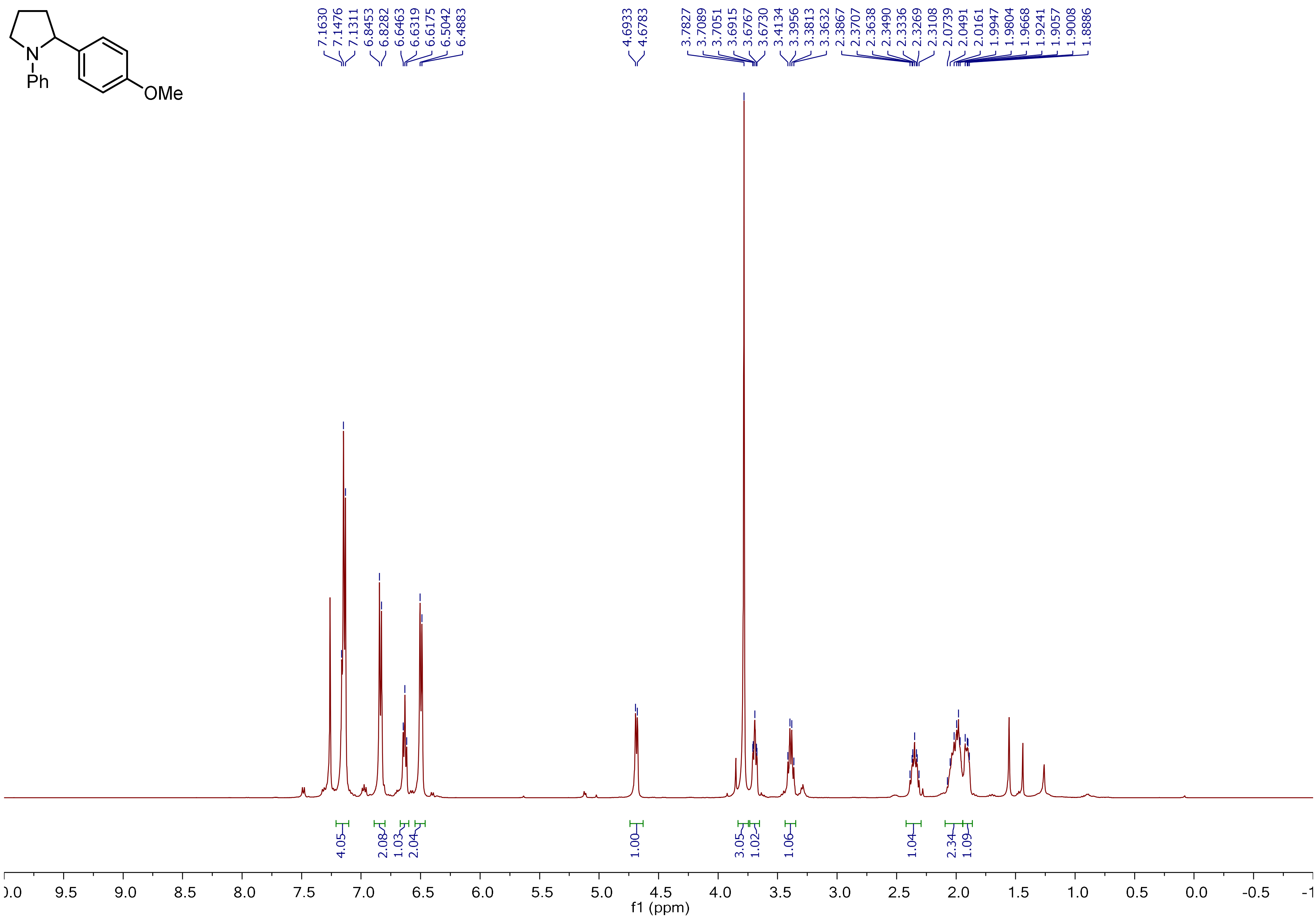

^{13}C NMR (125 MHz, $CDCl_3$): 2-(4-methoxyphenyl)-1-phenylpyrrolidine (7)

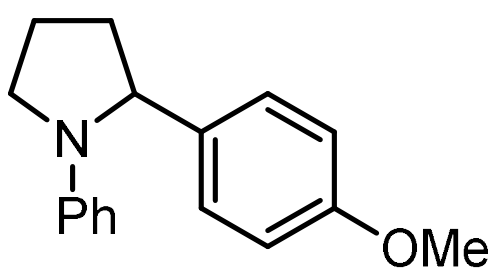

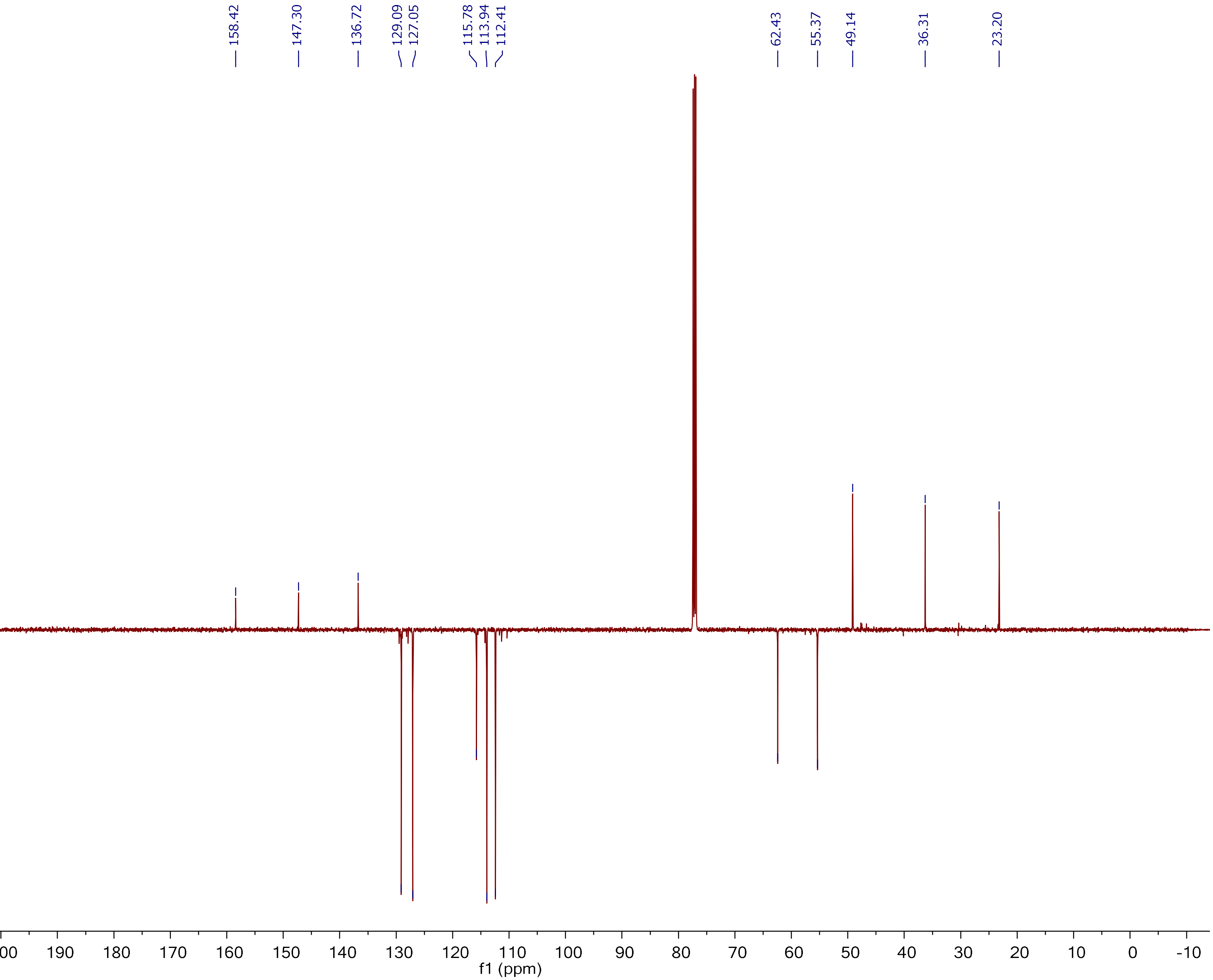

^{1}H NMR (500 MHz, $CDCl_3$): 2-(4-(methylthio)phenyl)-1-phenylpyrrolidine (8)

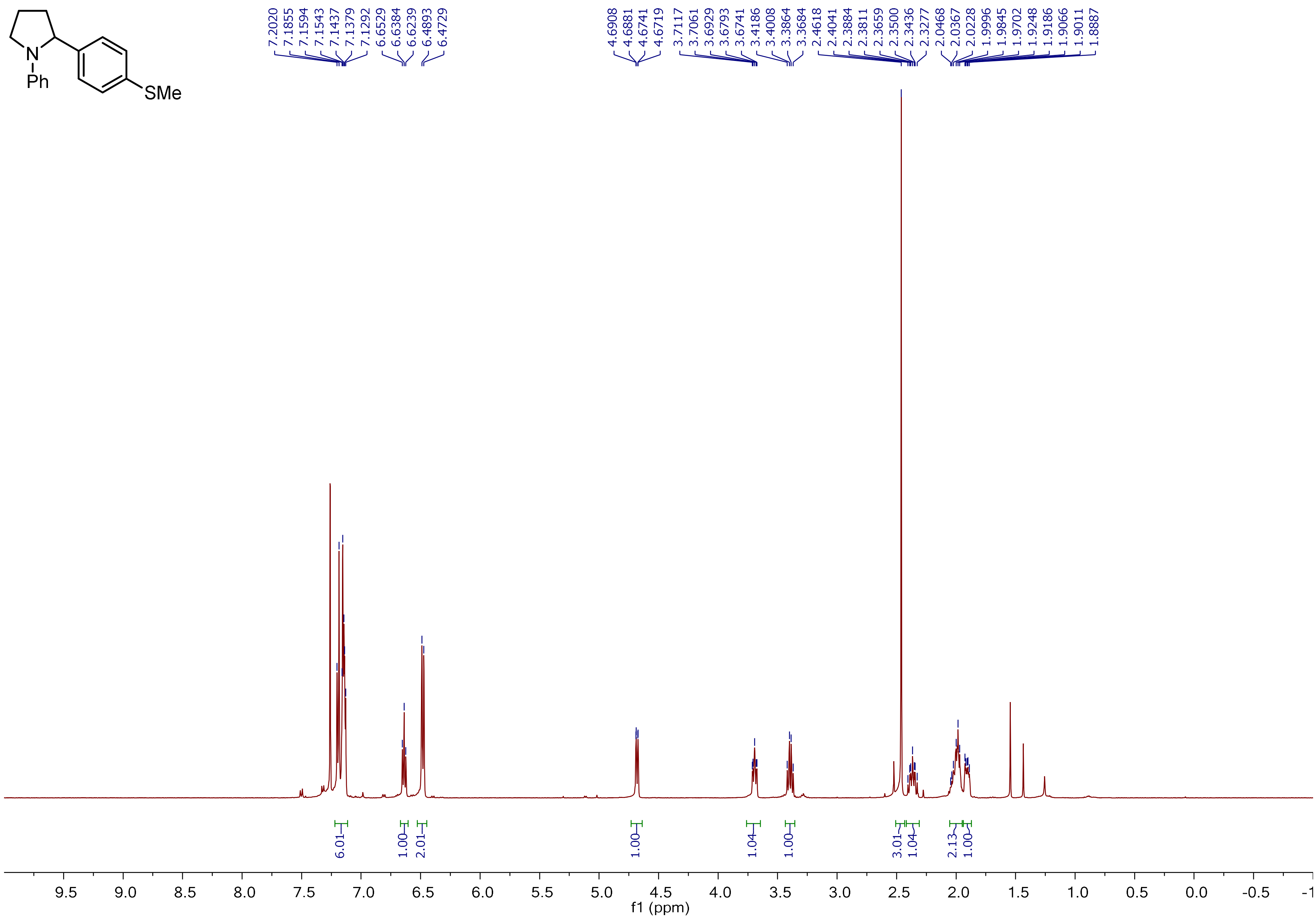

^{13}C NMR (125 MHz, $CDCl_3$): 2-(4-(methylthio)phenyl)-1-phenylpyrrolidine (8)

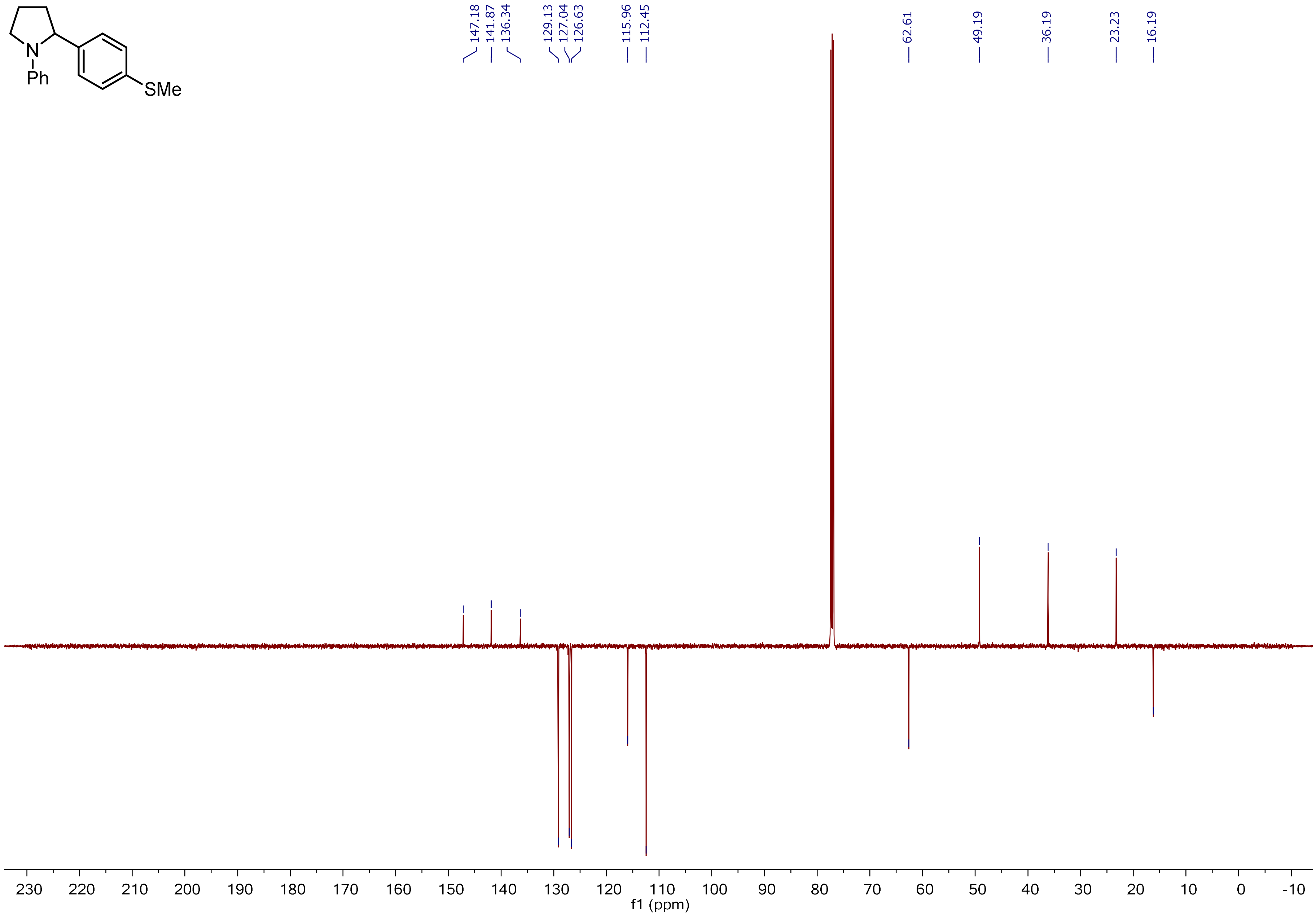

^{1}H NMR (500 MHz, $CDCl_3$): 2-(benzo[*d*][1,3]dioxol-5-yl)-1-phenylpyrrolidine (9)

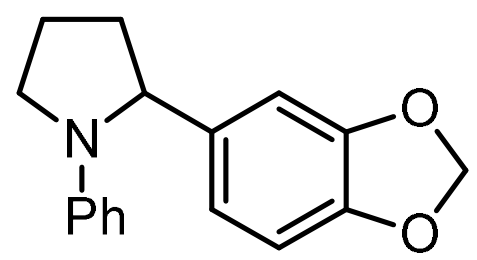

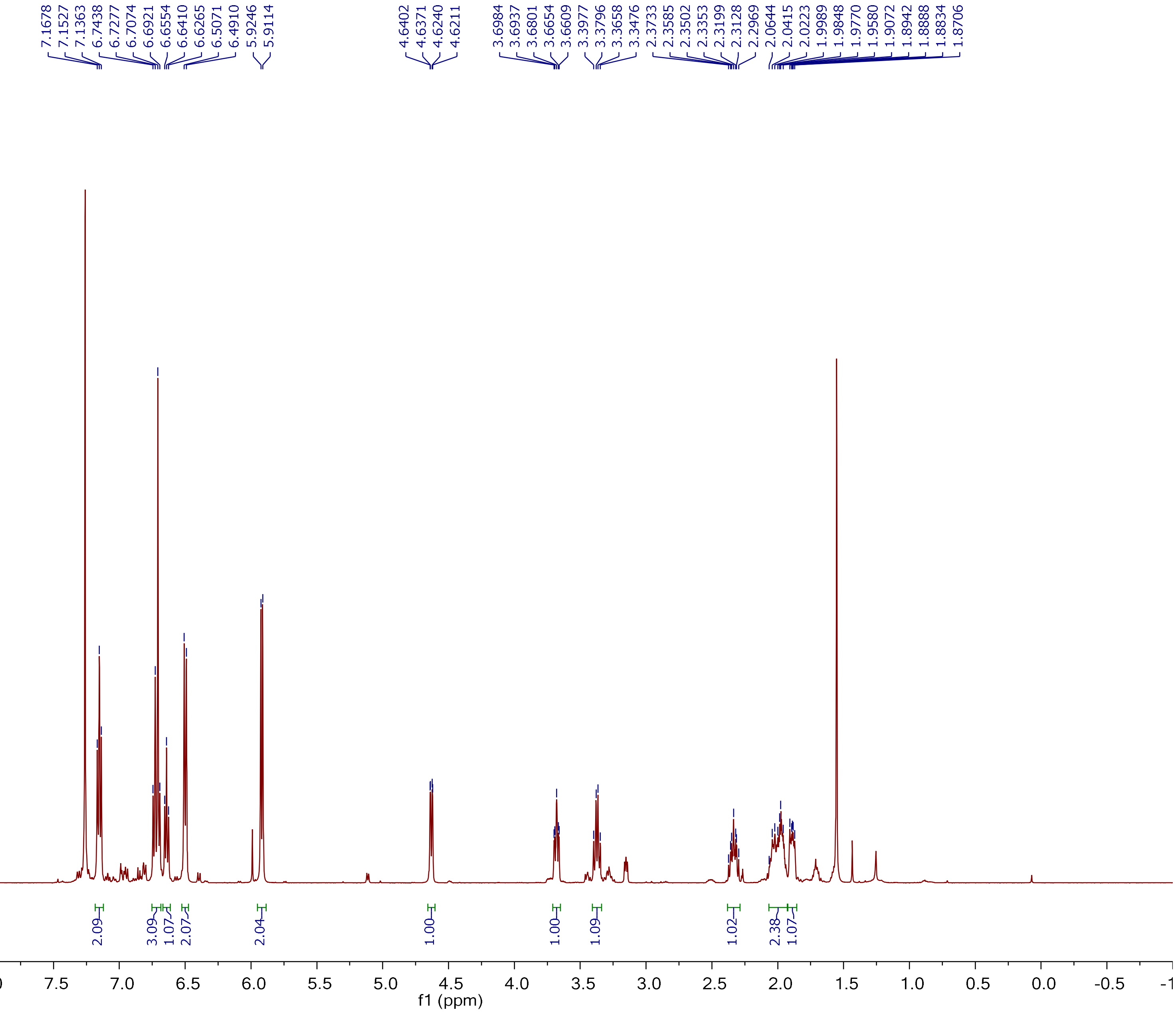

^{13}C NMR (125 MHz, $CDCl_3$): 2-(benzo[*d*][1,3]dioxol-5-yl)-1-phenylpyrrolidine (9)

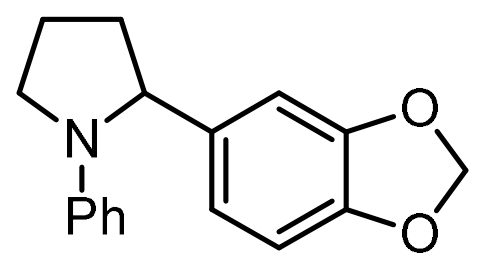

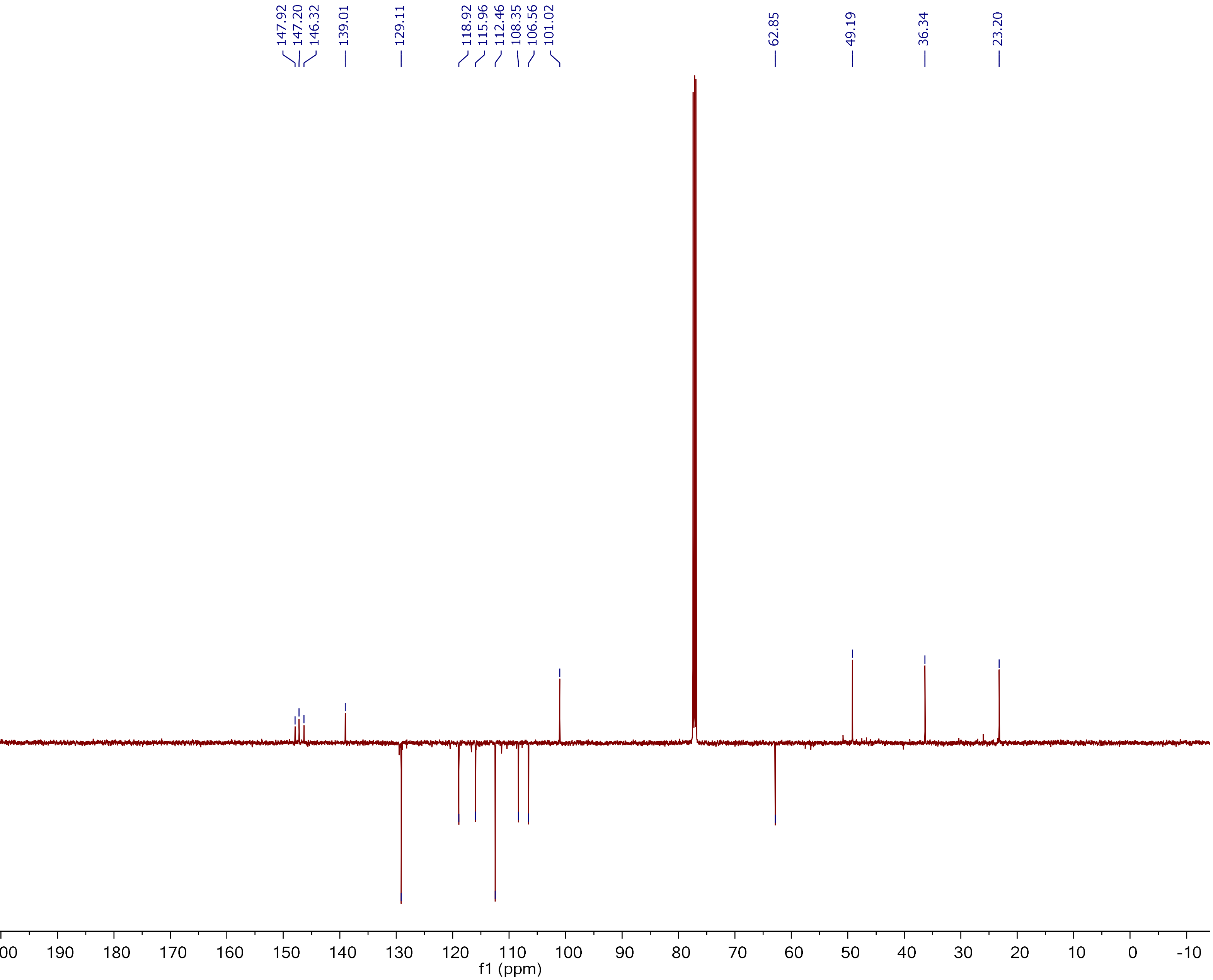

^{1}H NMR (500 MHz, $CDCl_3$): 2-(4-chlorophenyl)-1-phenylpyrrolidine (10)

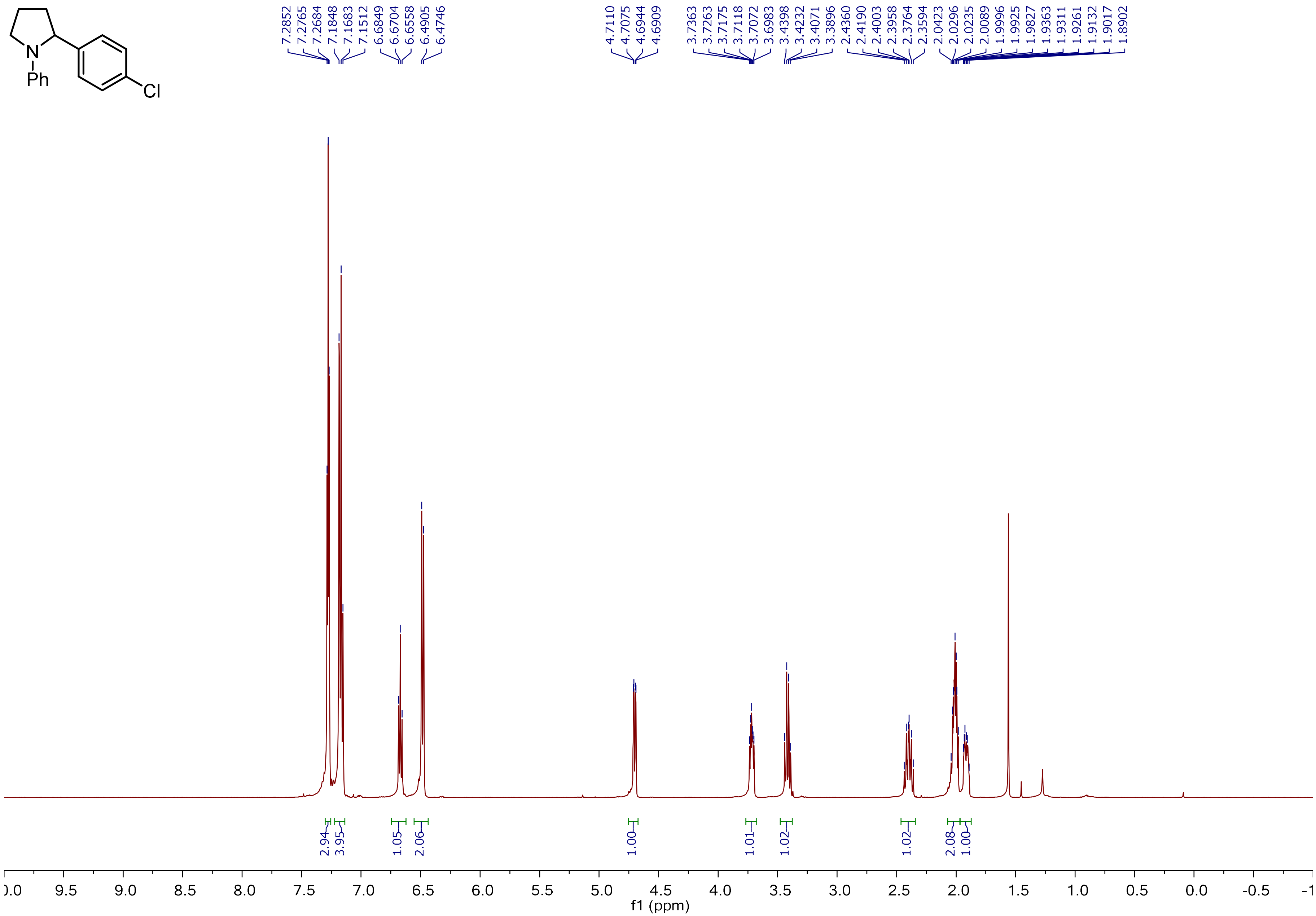

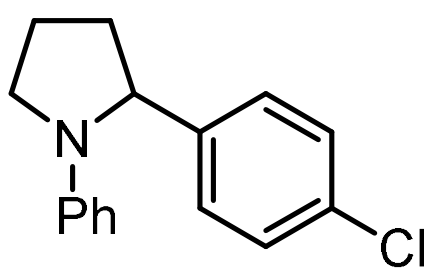
N
Ph
Cl

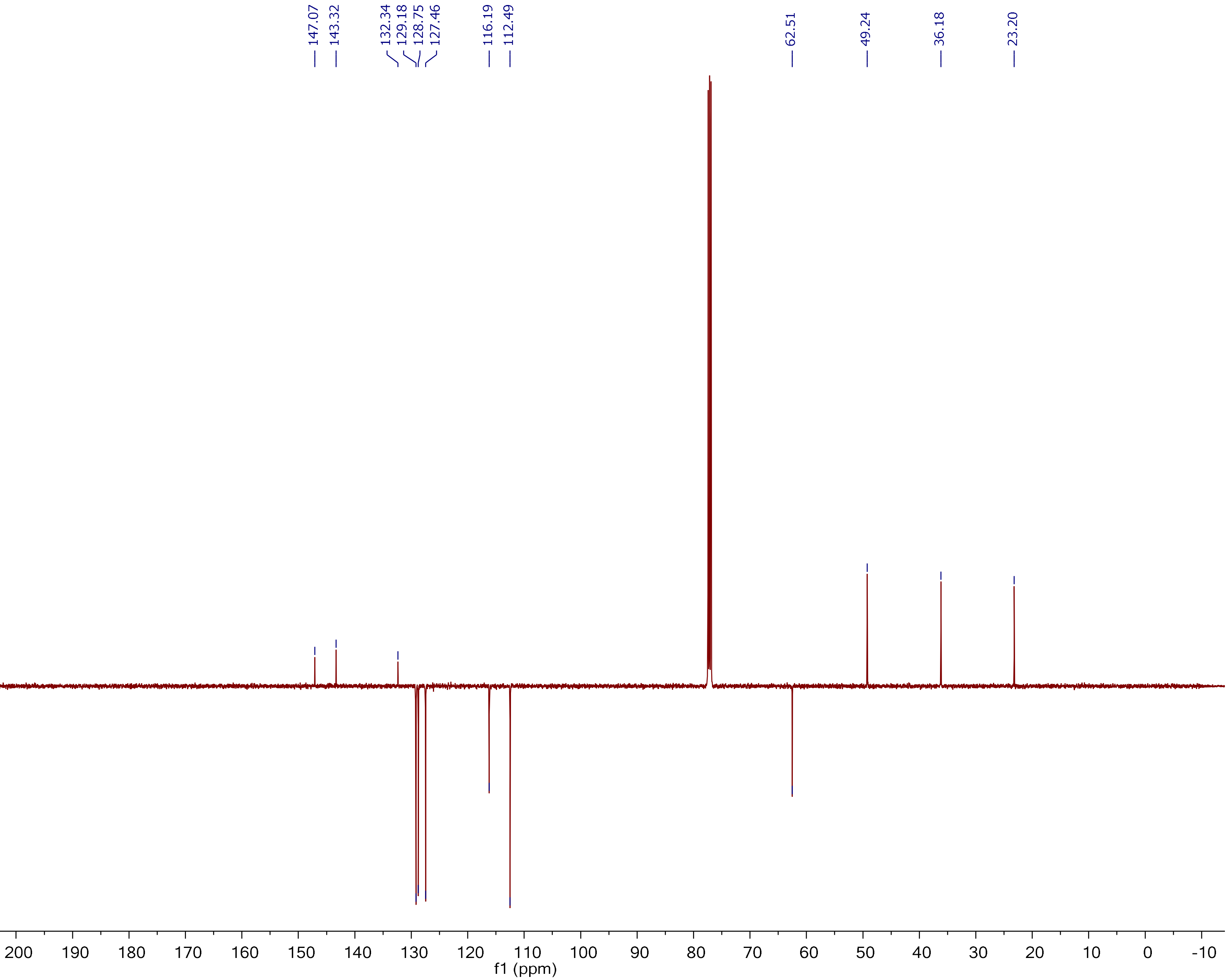
147.07
143.32
132.34
129.18
128.75
127.46
116.19
112.49
62.51
49.24
36.18
23.20
230
220
210
200
190
180
170
160
150
140
130
120
110
100
90
80
70
60
50
40
30
20
10
0
-10
f1 (ppm)

^{1}H NMR (500 MHz, $CDCl_3$): *tert*-butyl 5-(1-phenylpyrrolidin-2-yl)-1*H*-indole-1-carboxylate (11)

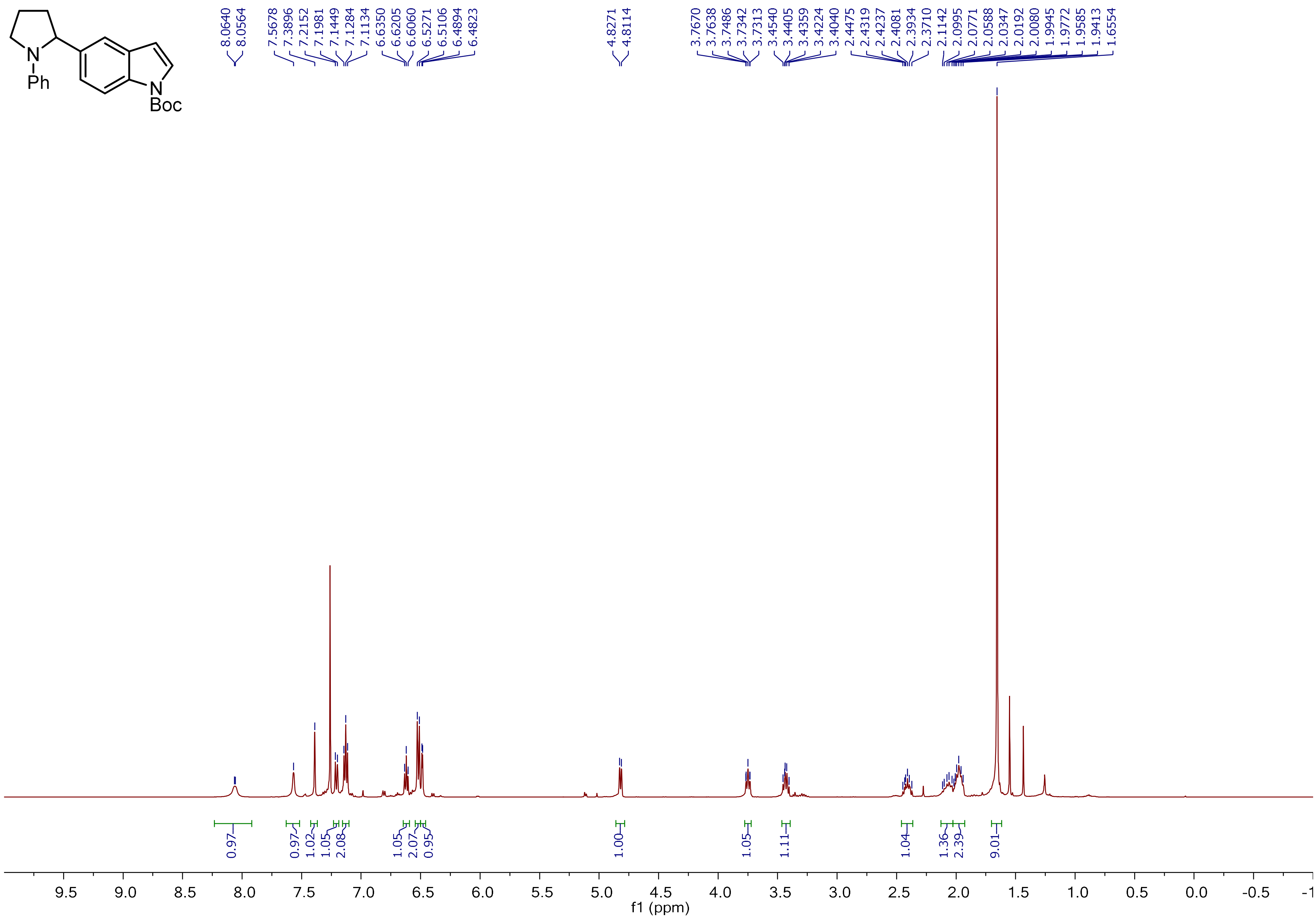

^{13}C NMR (125 MHz, CDCl$_3$): *tert*-butyl 5-(1-phenylpyrrolidin-2-yl)-1*H*-indole-1-carboxylate (11)

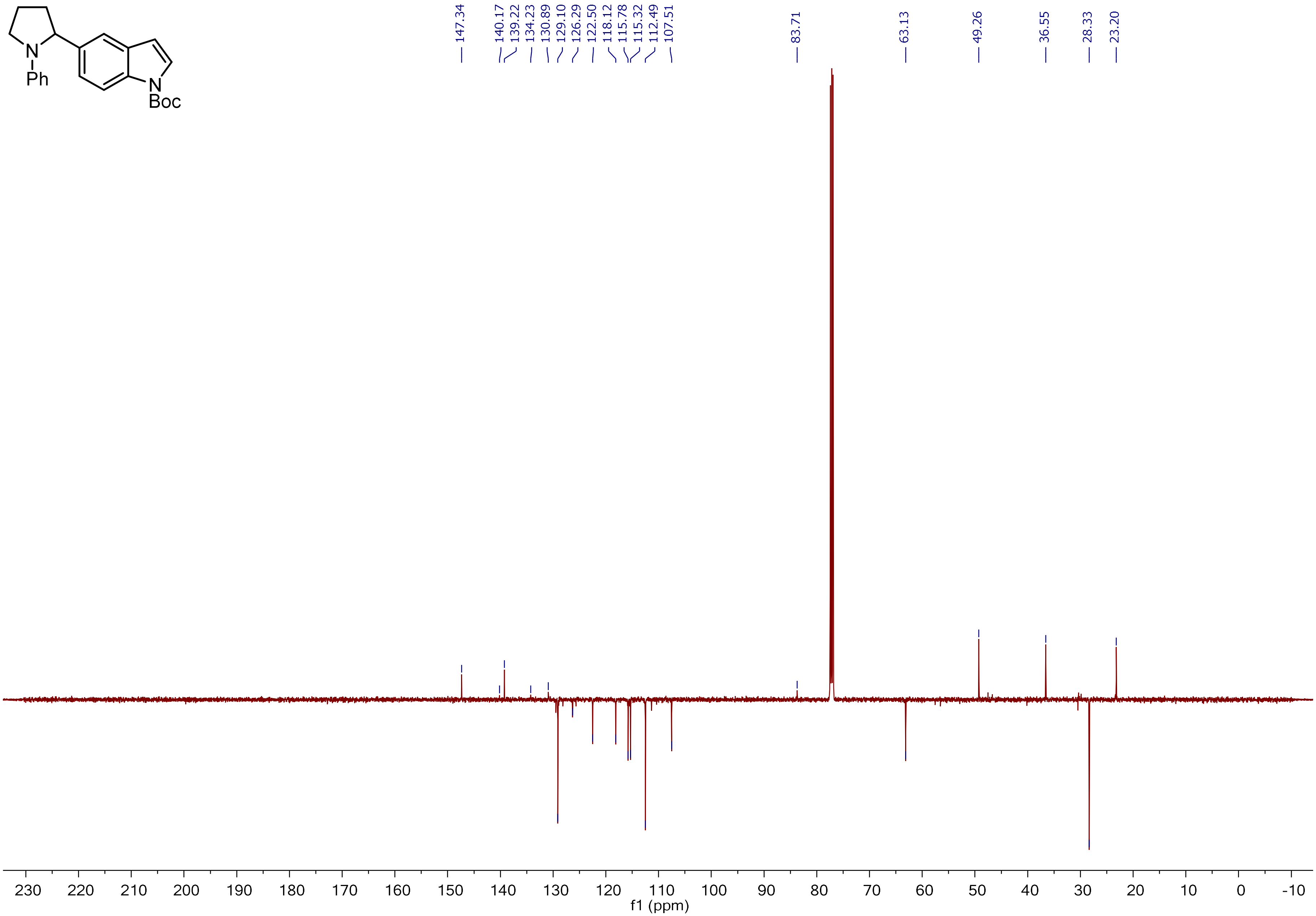

^{1}H NMR (500 MHz, $CDCl_3$): 6-(1-phenylpyrrolidin-2-yl)quinoline (12)

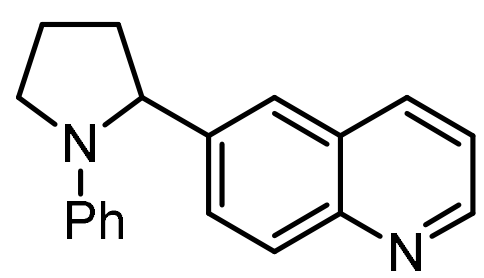

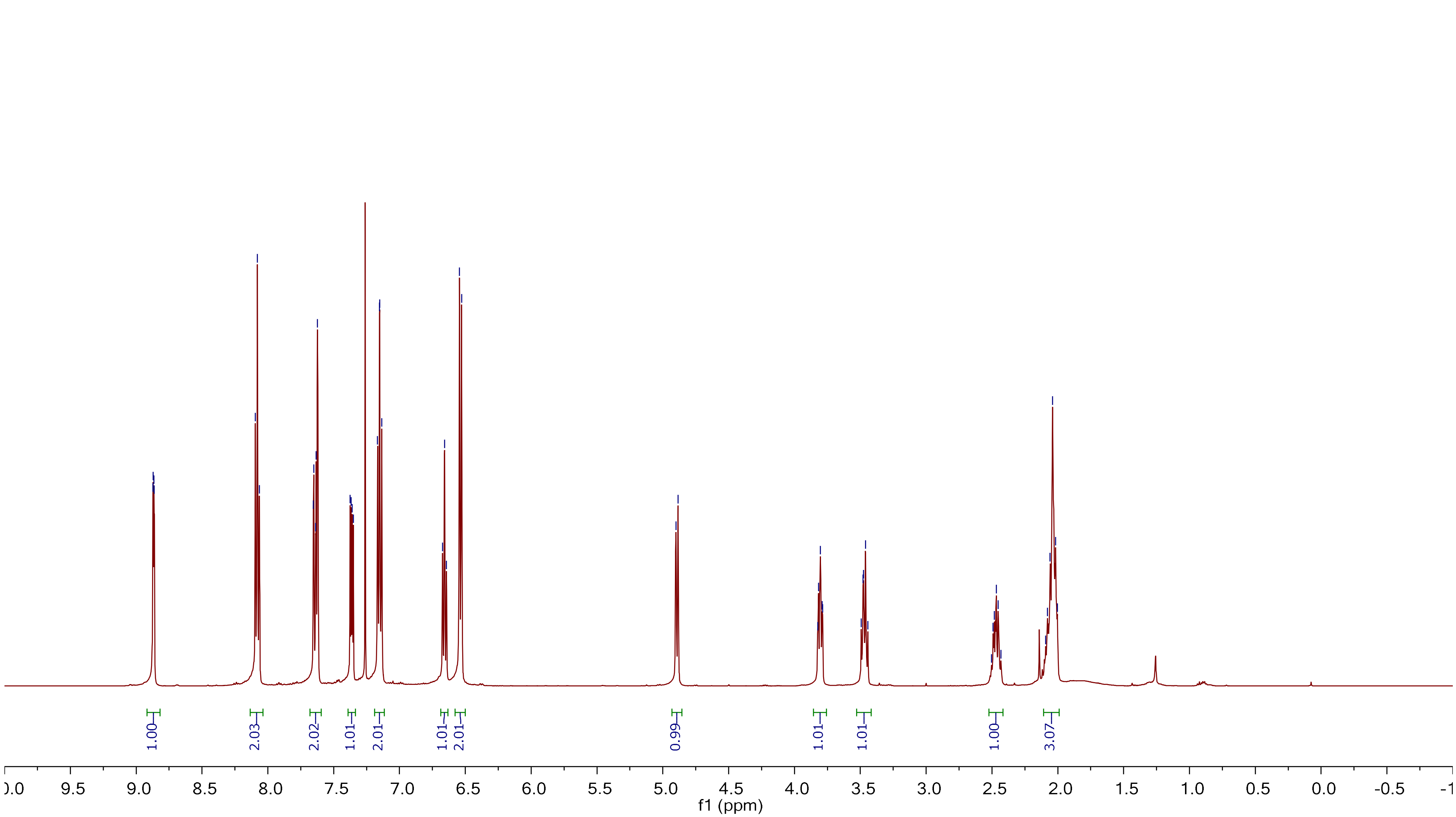

^{13}C NMR (125 MHz, $CDCl_3$): 6-(1-phenylpyrrolidin-2-yl)quinoline (12)

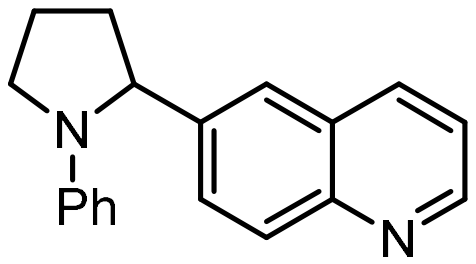

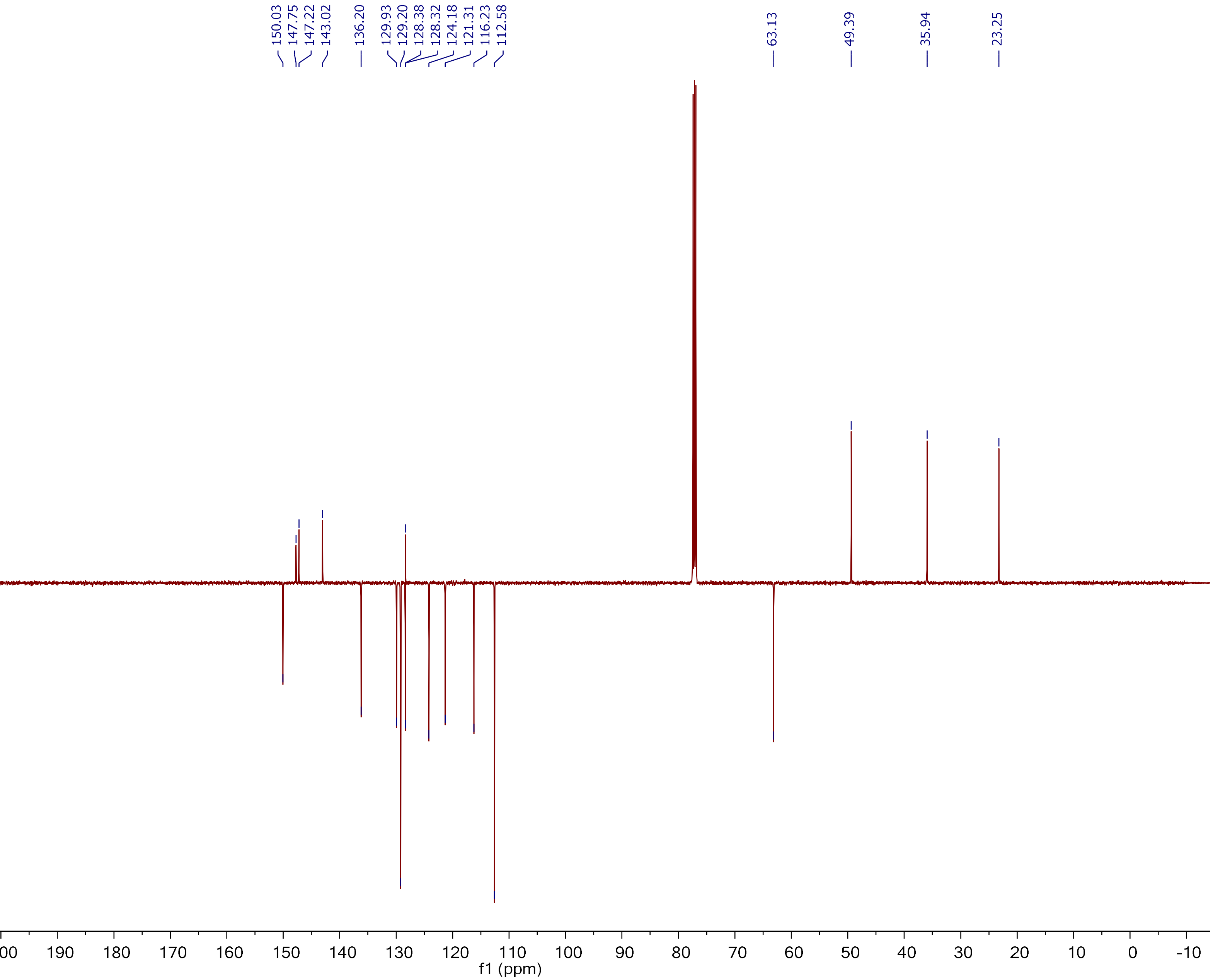

^{1}H NMR (500 MHz, $CDCl_3$): 5-(1-phenylpyrrolidin-2-yl)-2-(trifluoromethyl)pyridine (13)

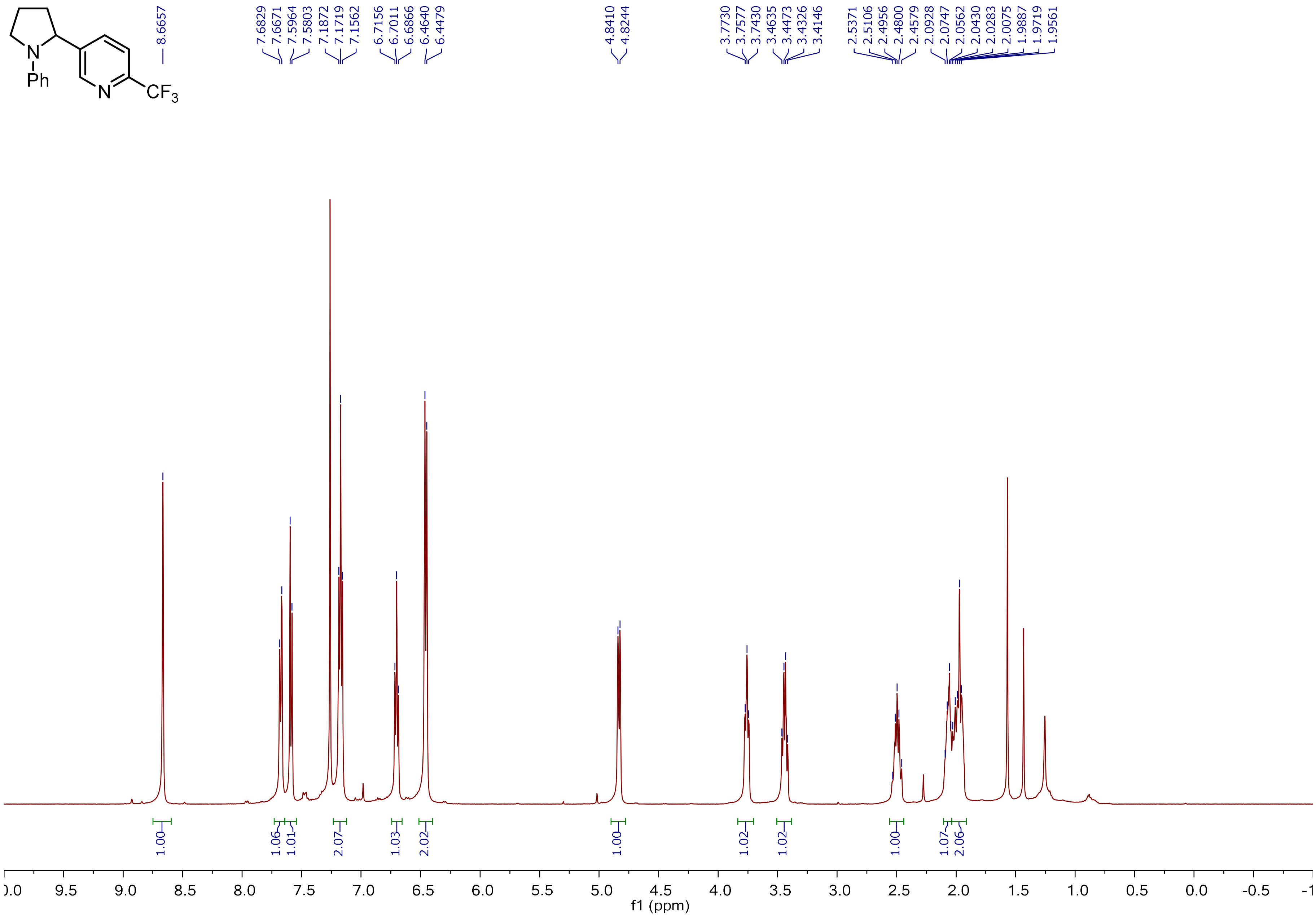

^{13}C NMR (125 MHz, $CDCl_3$): 5-(1-phenylpyrrolidin-2-yl)-2-(trifluoromethyl)pyridine (13)

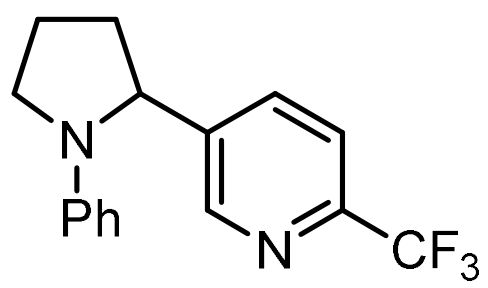

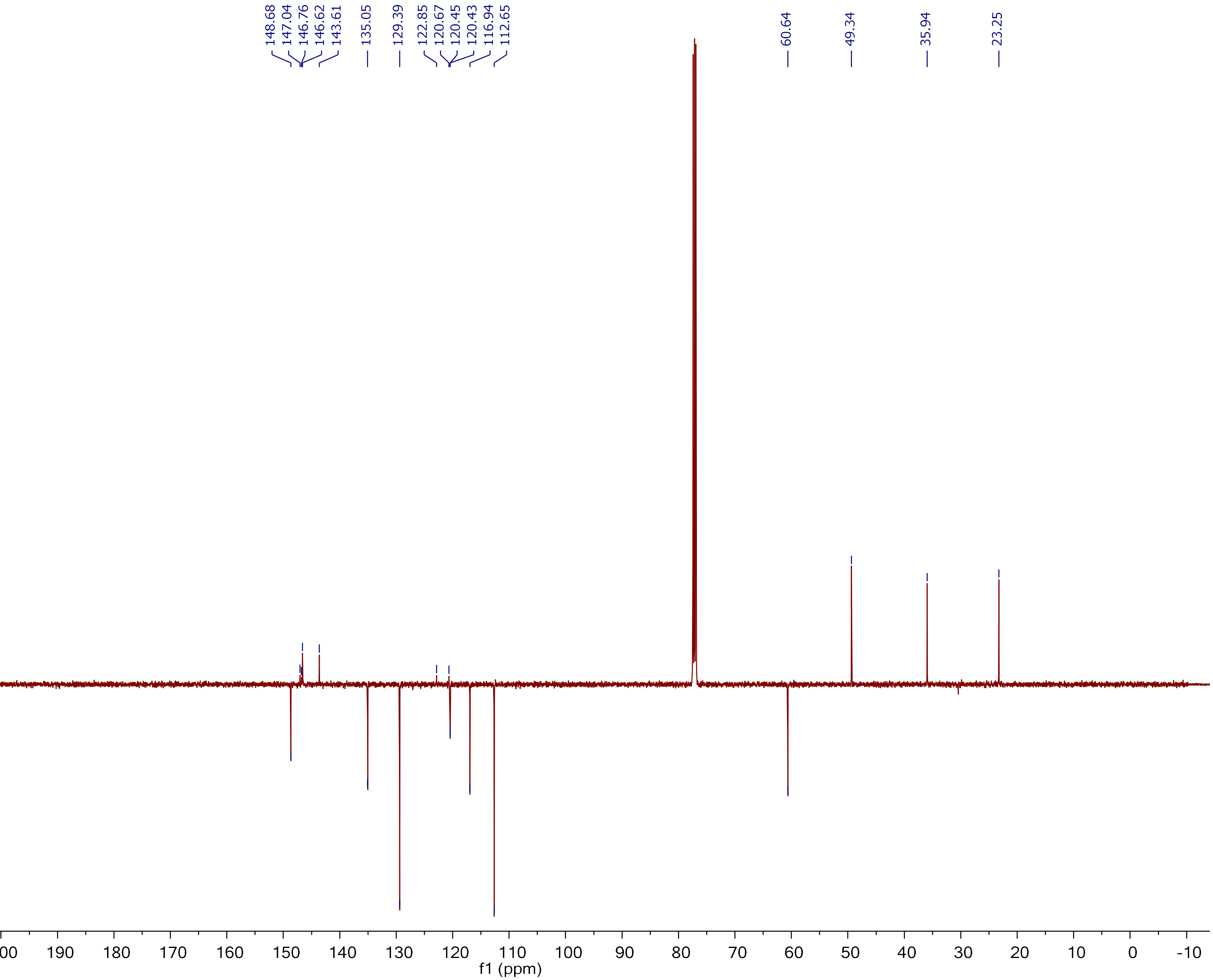

^{19}F NMR (282 MHz, $CDCl_3$): 5-(1-phenylpyrrolidin-2-yl)-2-(trifluoromethyl)pyridine (13)

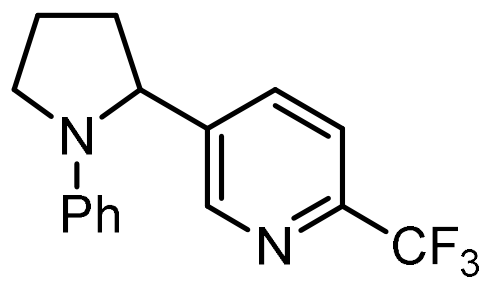

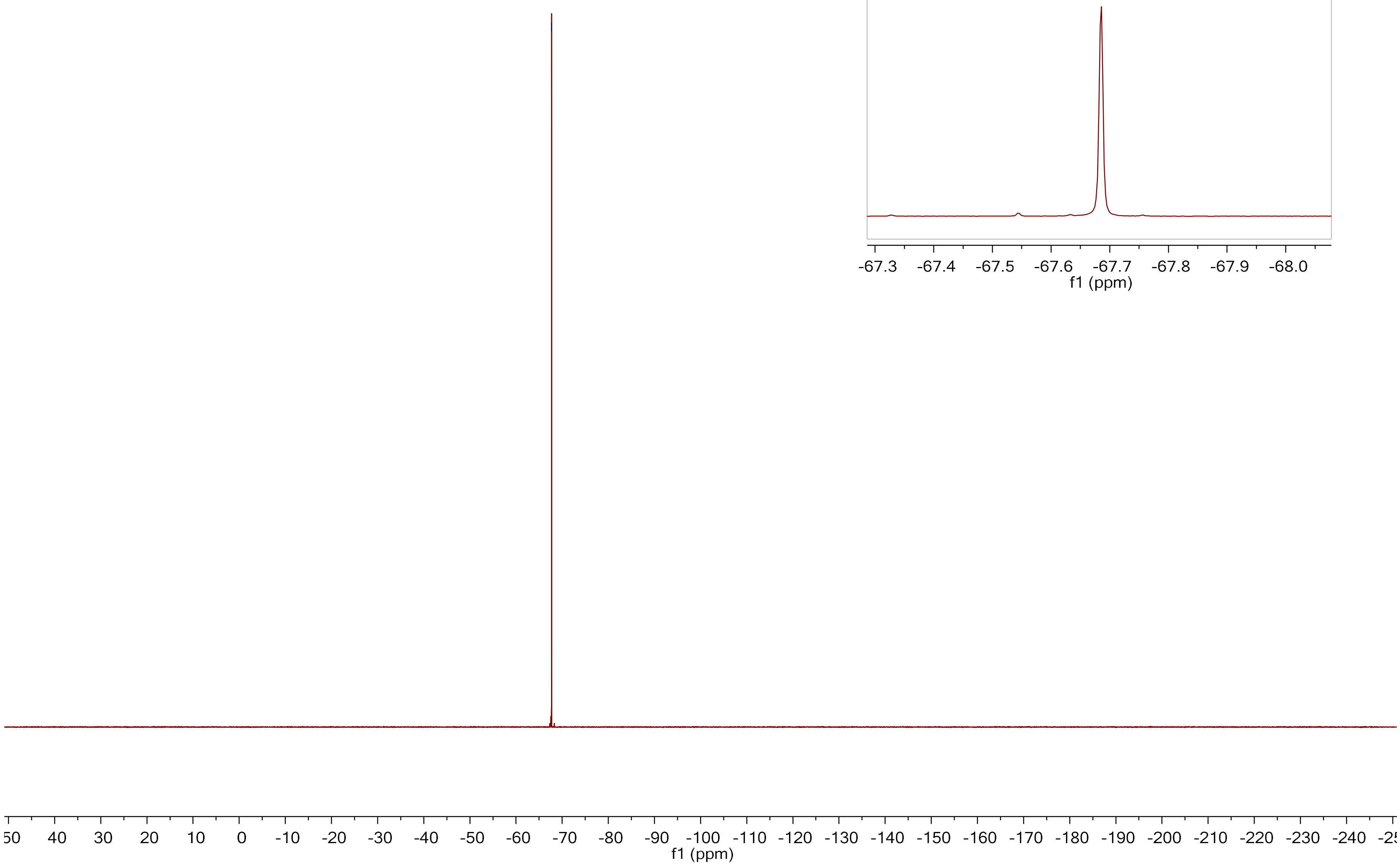

^{1}H NMR (500 MHz, $CDCl_3$): 2-methoxy-5-(1-phenylpyrrolidin-2-yl)pyridine (14)

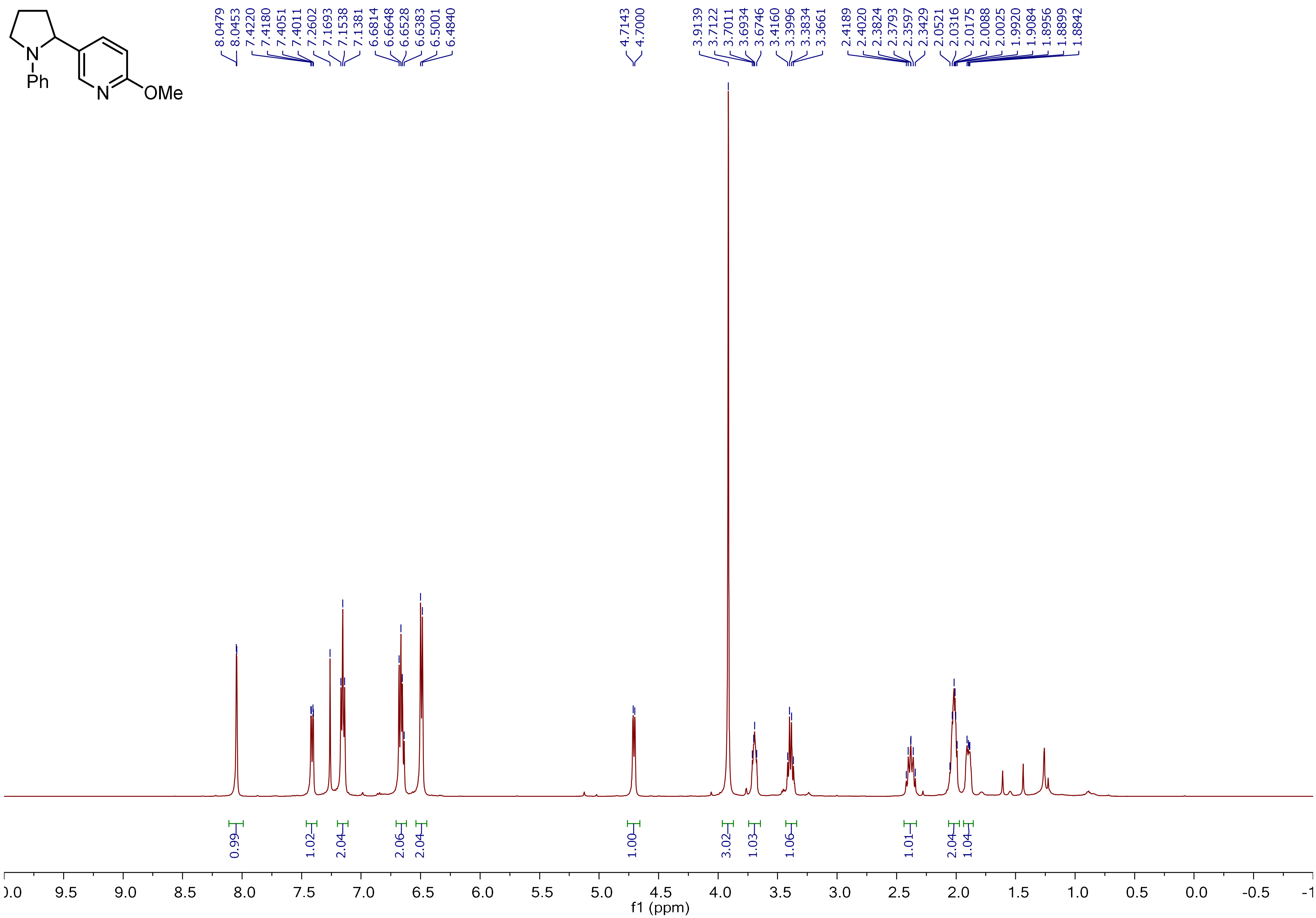

^{13}C NMR (125 MHz, $CDCl_3$): 2-methoxy-5-(1-phenylpyrrolidin-2-yl)pyridine (14)

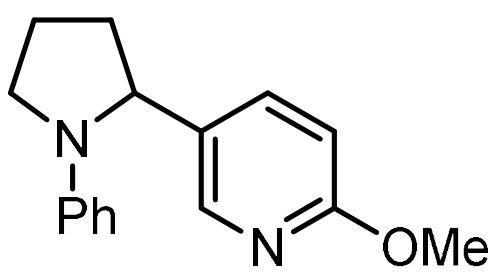

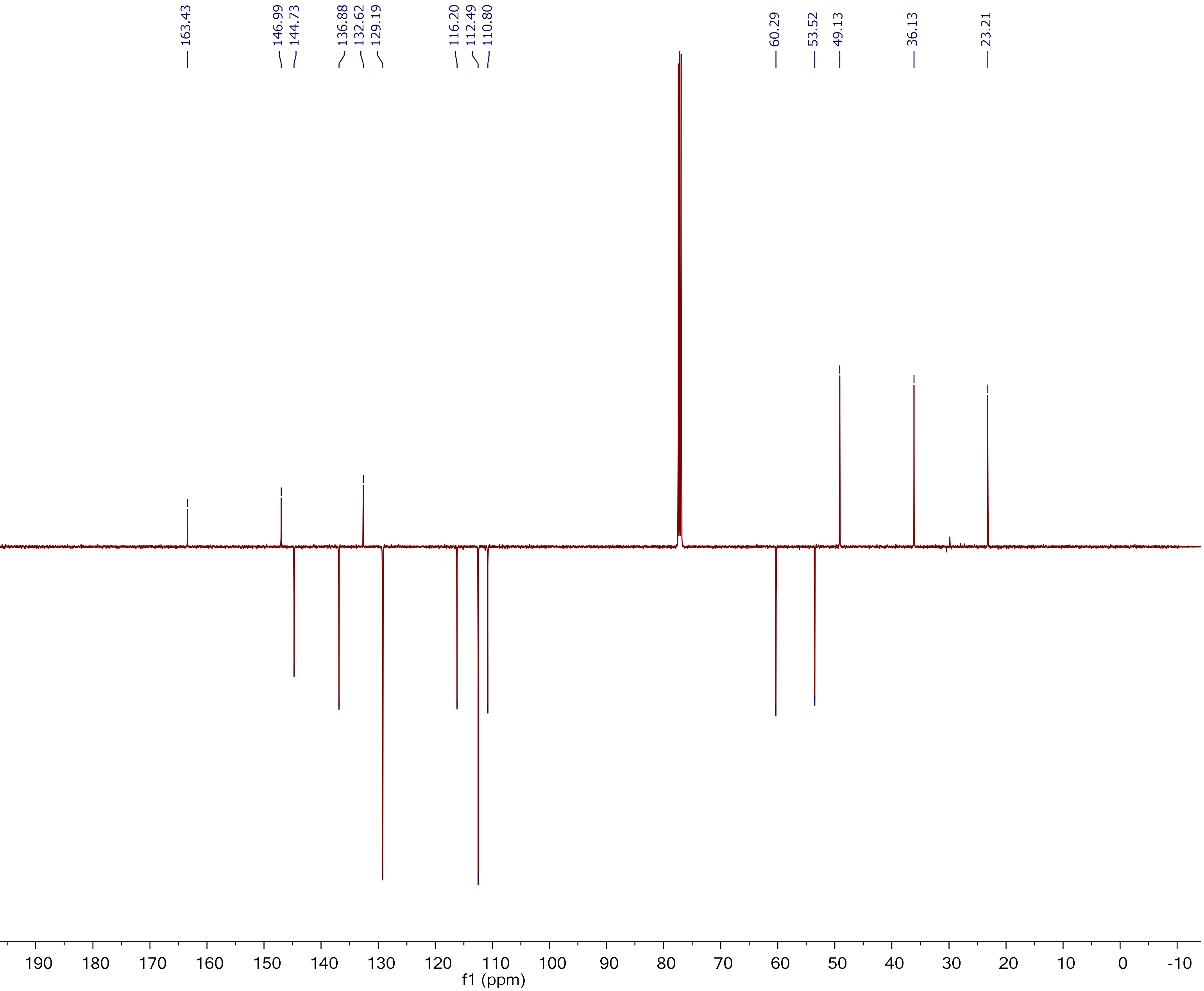

^{1}H NMR (500 MHz, $CDCl_3$): 2-(cyclohex-1-en-1-yl)-1-phenylpyrrolidine (15)

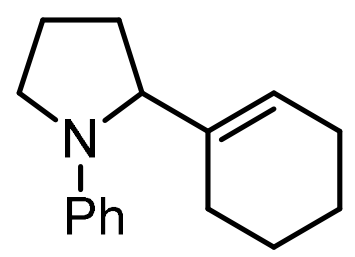

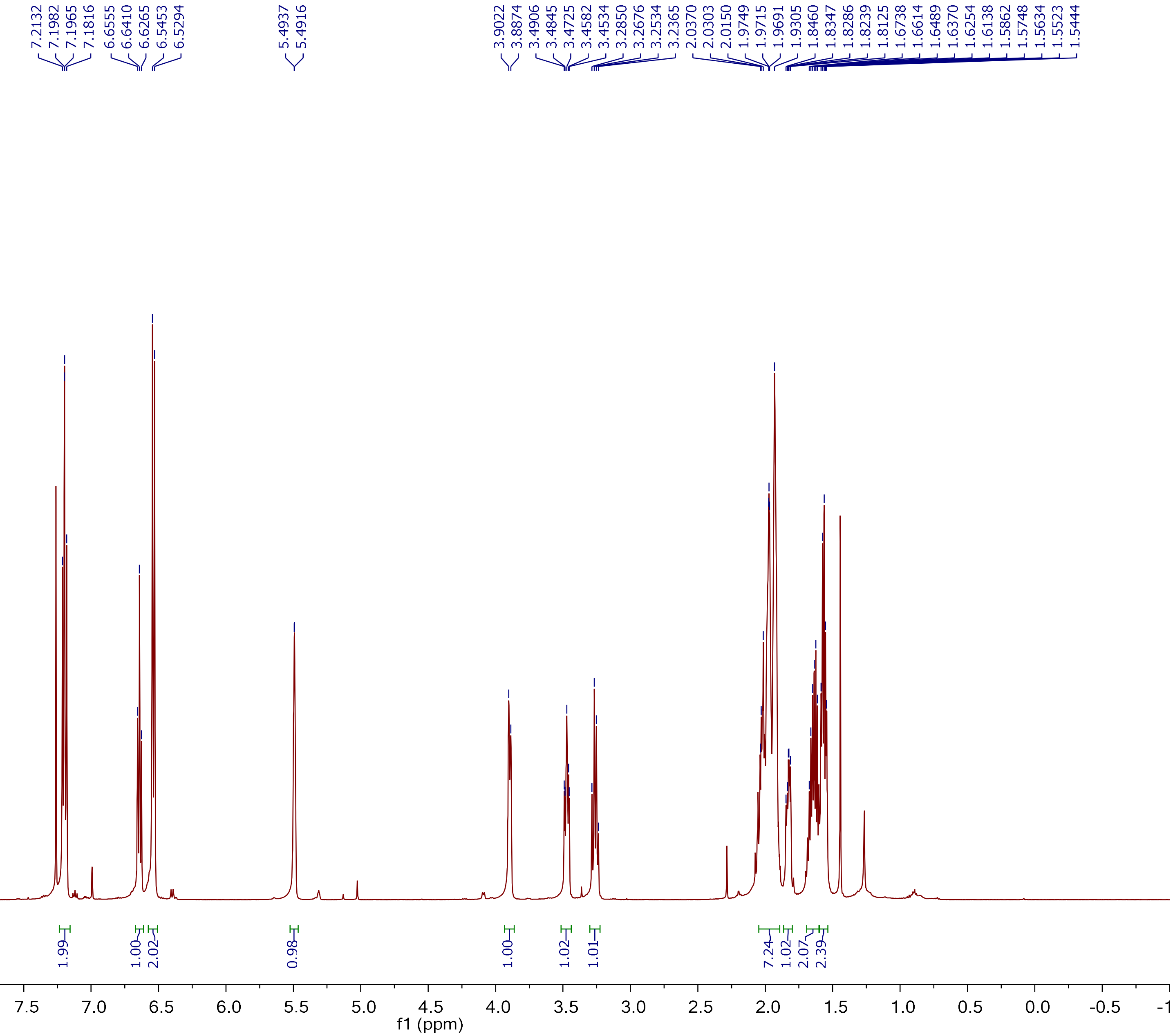

^{13}C NMR (125 MHz, $CDCl_3$): 2-(cyclohex-1-en-1-yl)-1-phenylpyrrolidine (15)

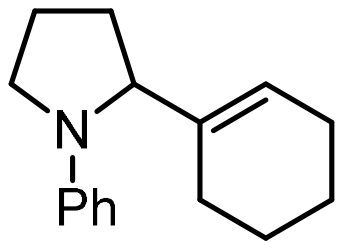

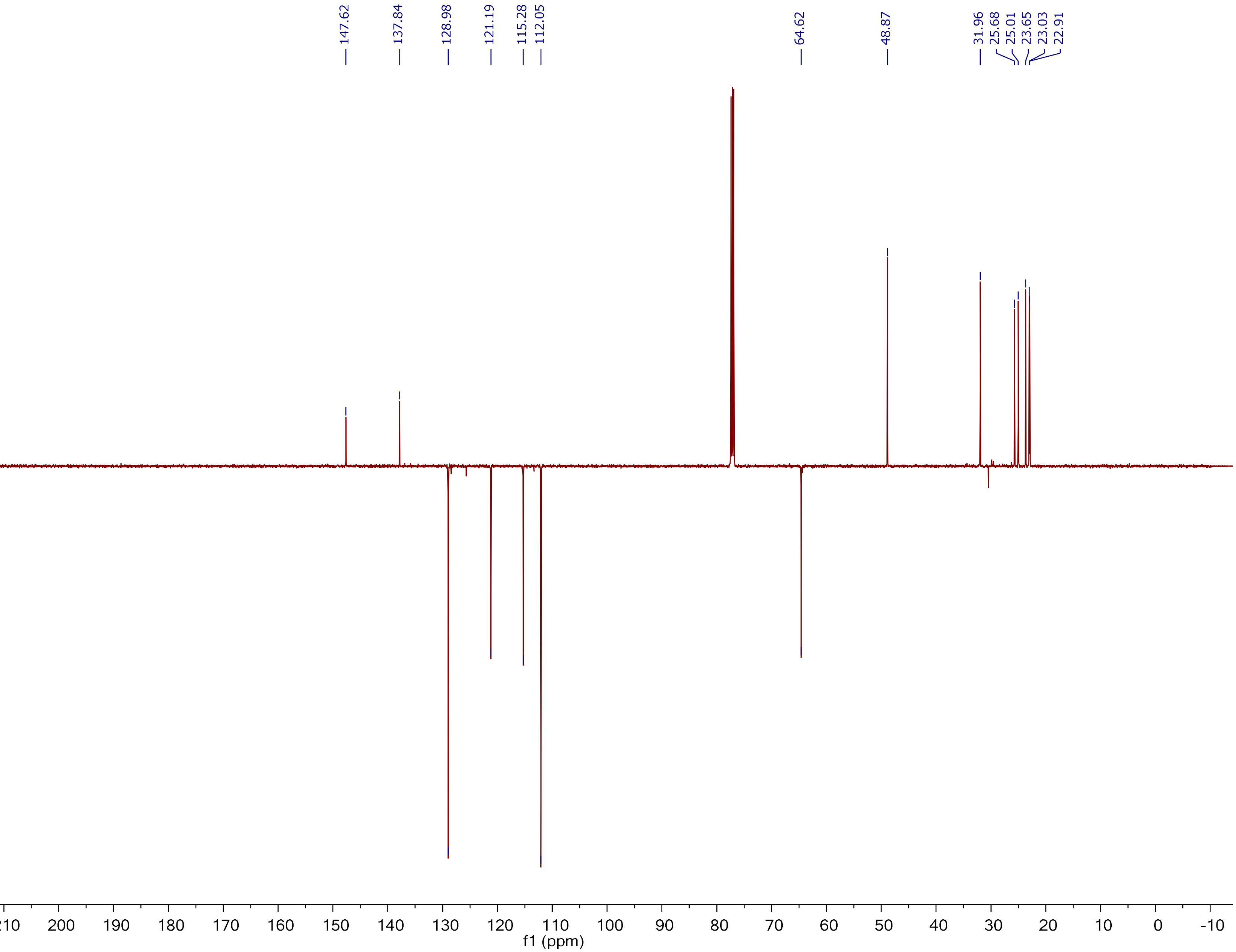

^{1}H NMR (500 MHz, CDCl$_3$): 2-(2-(*p*-tolyl)pyrrolidin-1-yl)pyridine (16)

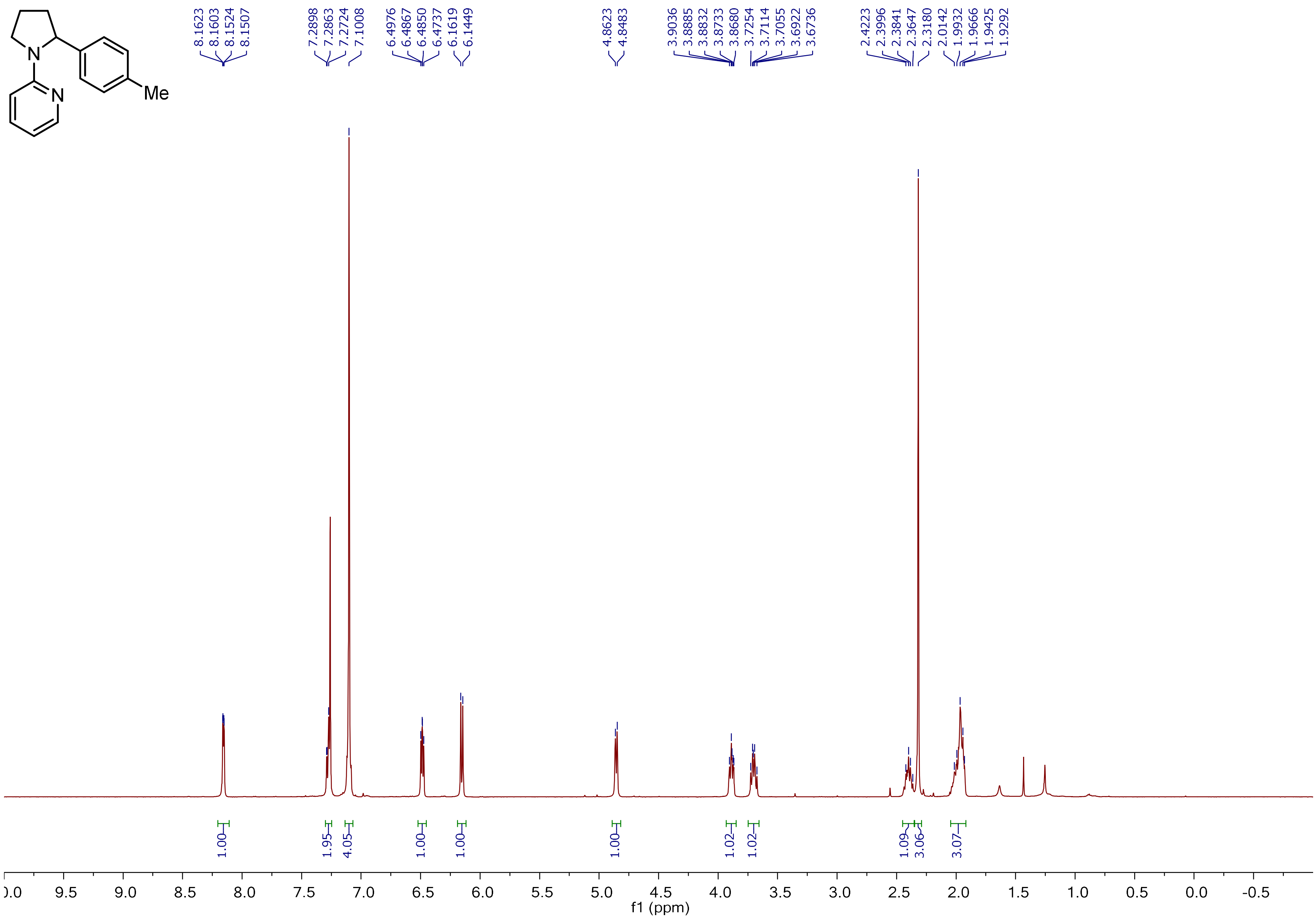

^{13}C NMR (125 MHz, $CDCl_3$): 2-(2-(*p*-tolyl)pyrrolidin-1-yl)pyridine (16)

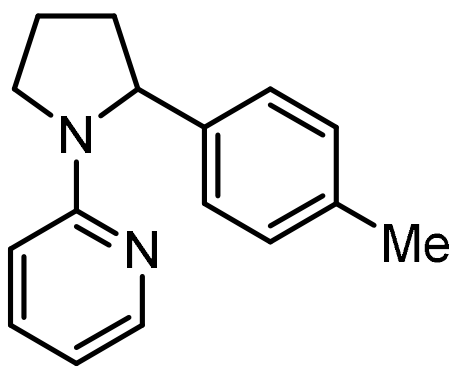

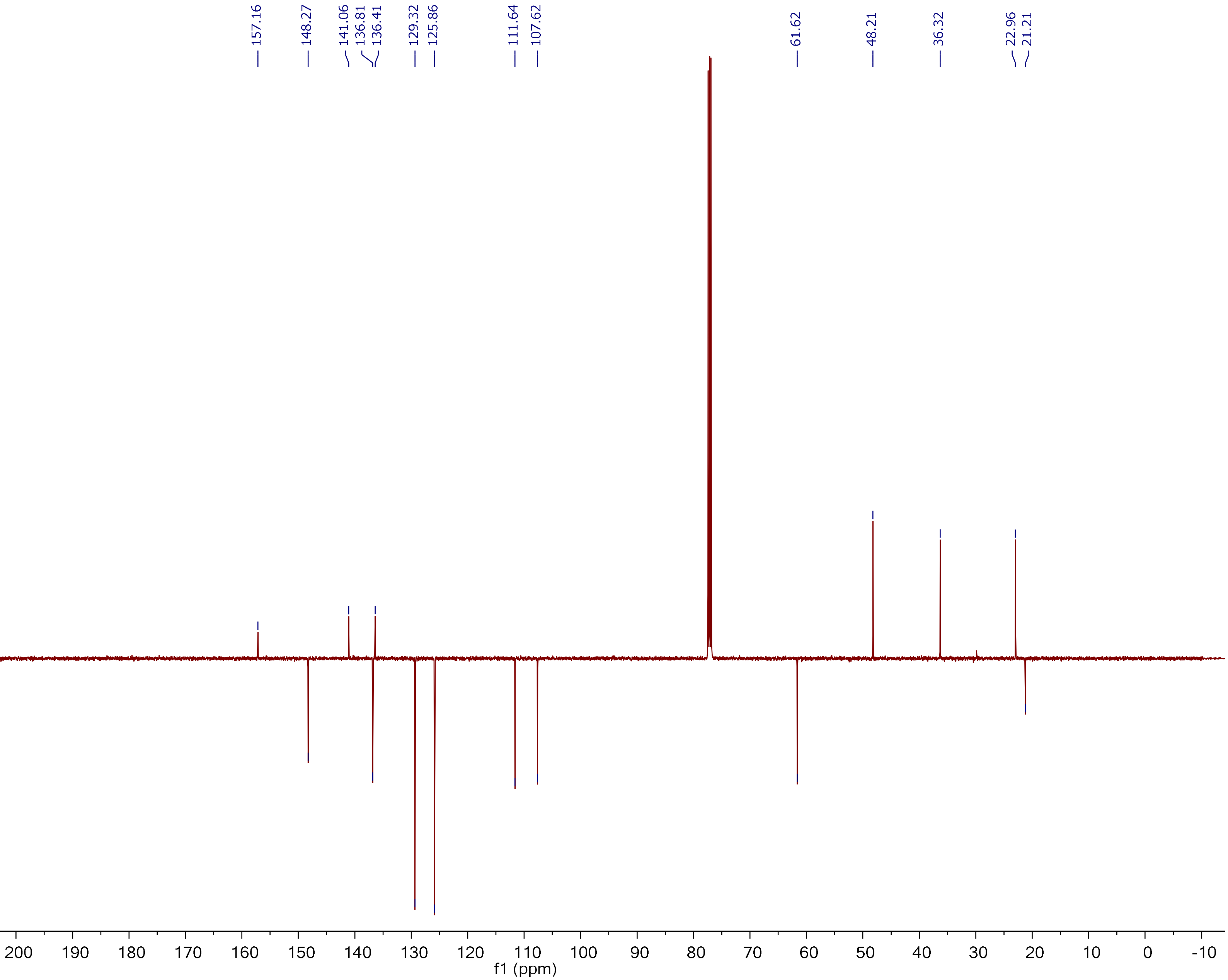

^{1}H NMR (500 MHz, $CDCl_3$): *N*-methyl-*N*-(4-methylbenzyl)aniline (17)

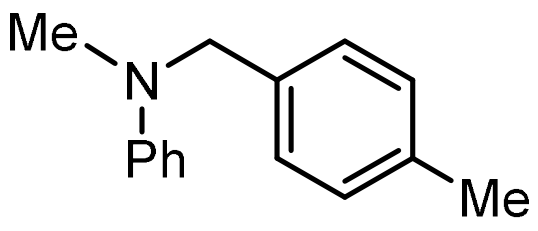

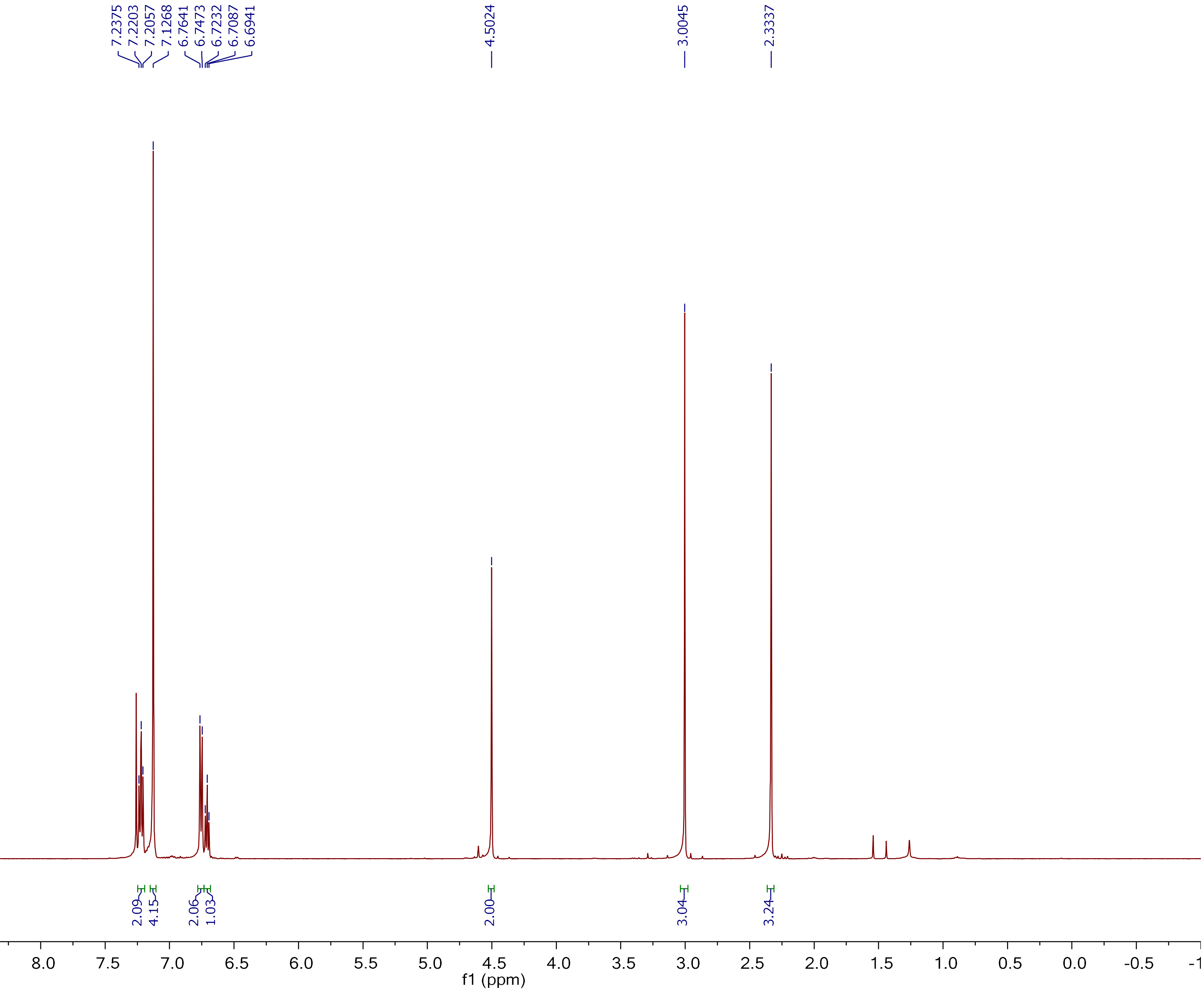

^{13}C NMR (125 MHz, $CDCl_3$): *N*-methyl-*N*-(4-methylbenzyl)aniline (17)

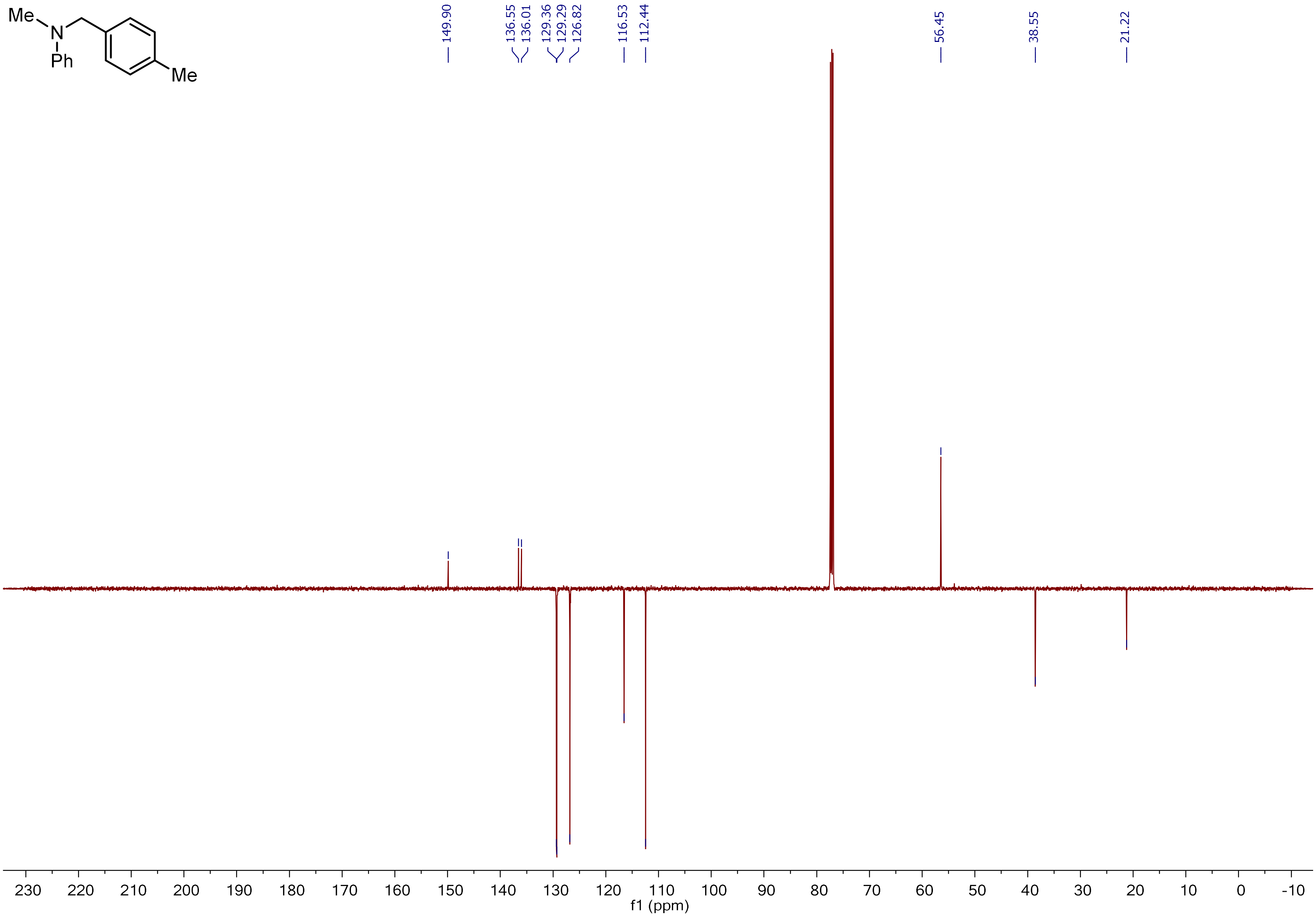

^{1}H NMR (500 MHz, $CDCl_3$): *N*-ethyl-*N*-(4-methylbenzyl)aniline (18)

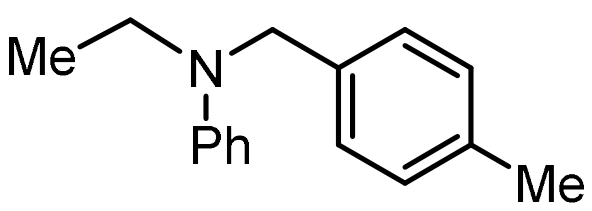

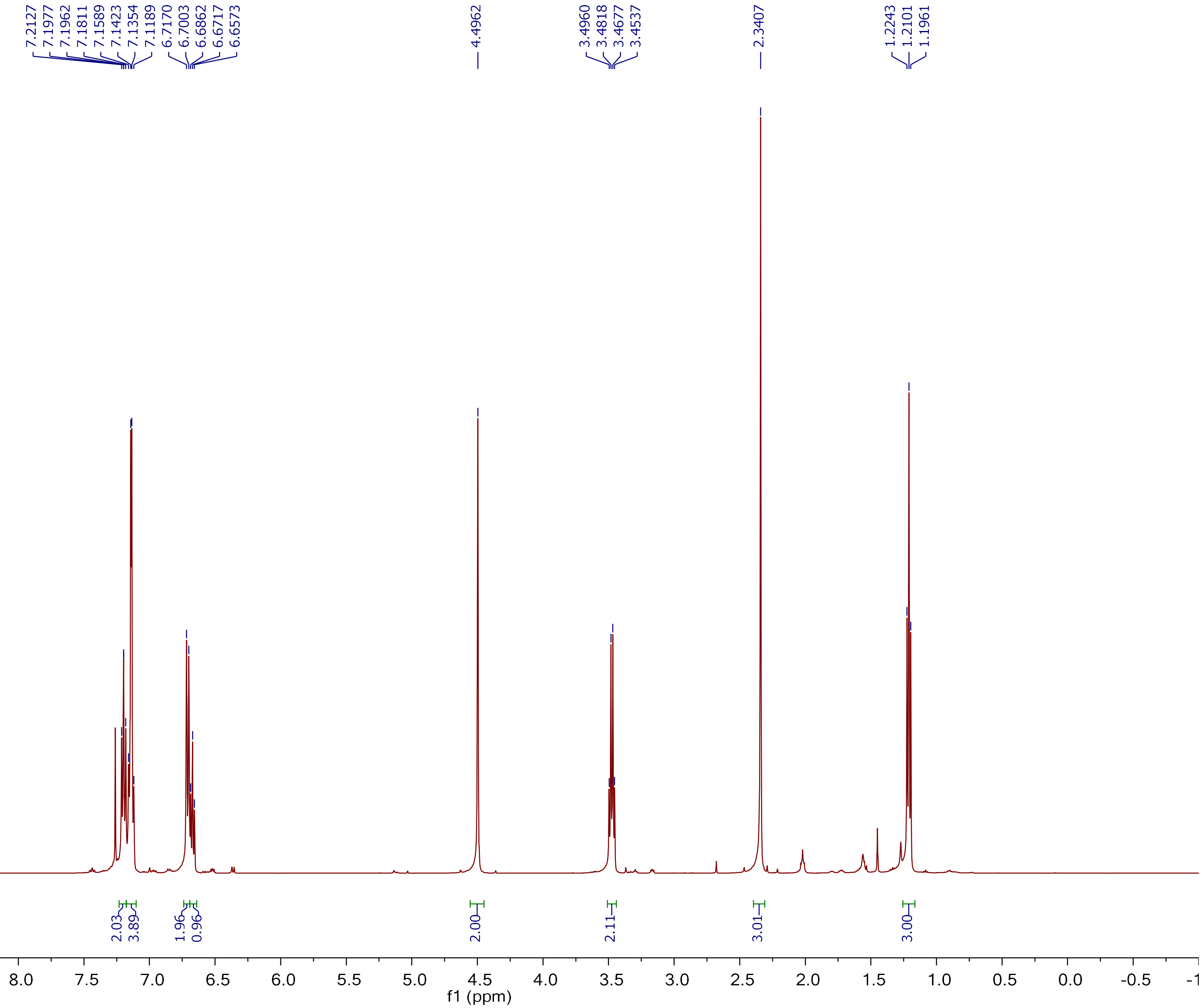

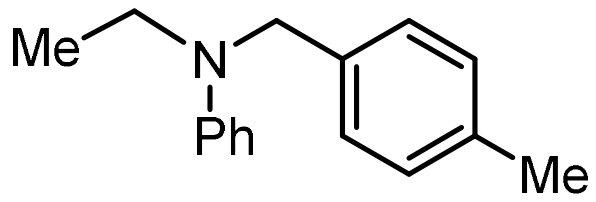
Me
N
Ph
Me

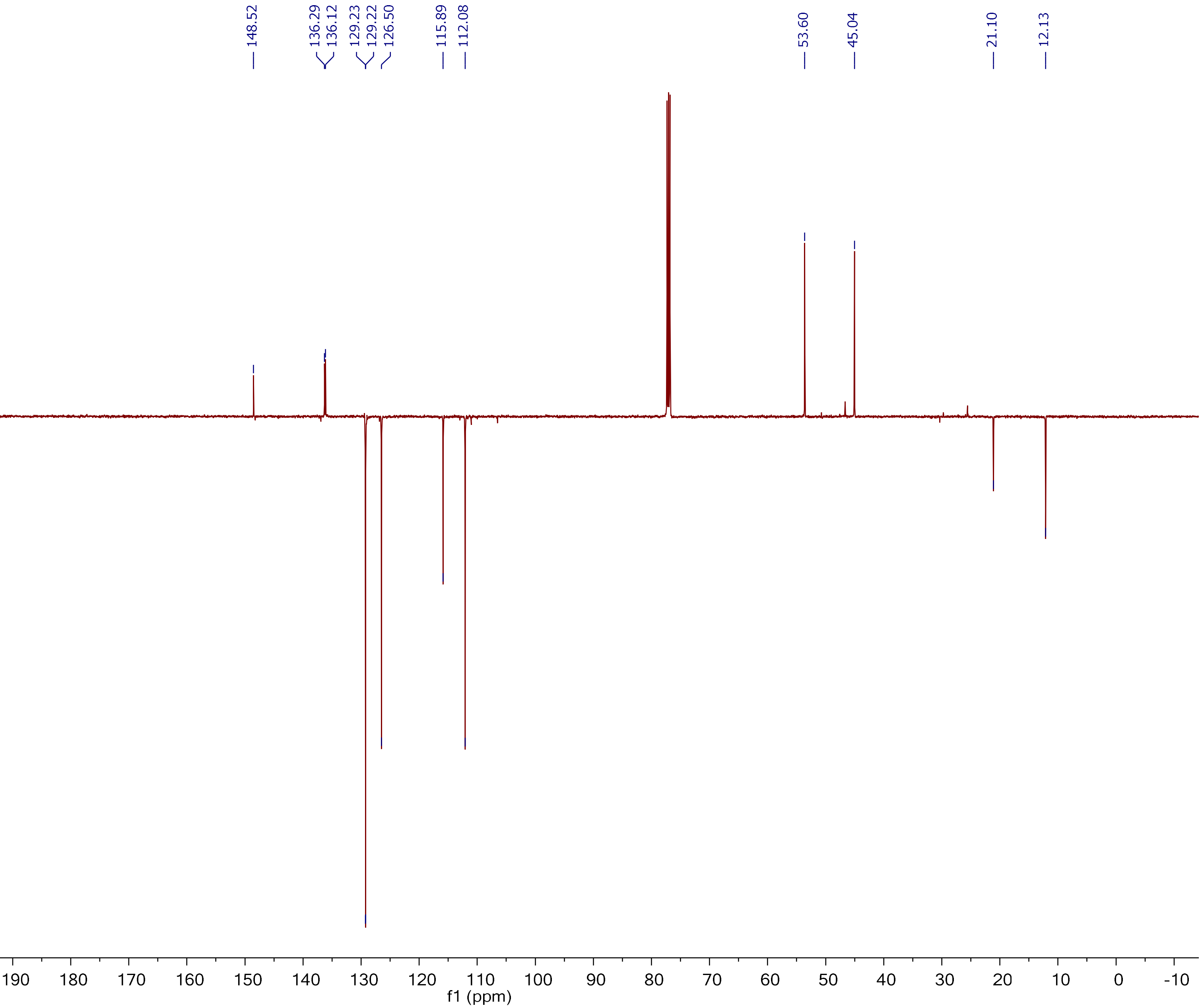
148.52
136.29
136.12
129.23
129.22
126.50
115.89
112.08
53.60
45.04
21.10
12.13
230
220
210
200
190
180
170
160
150
140
130
120
110
100
90
80
70
60
50
40
30
20
10
0
-10
f1 (ppm)

^{1}H NMR (500 MHz, $CDCl_3$): 1-phenyl-2-(*p*-tolyl)indoline (19)

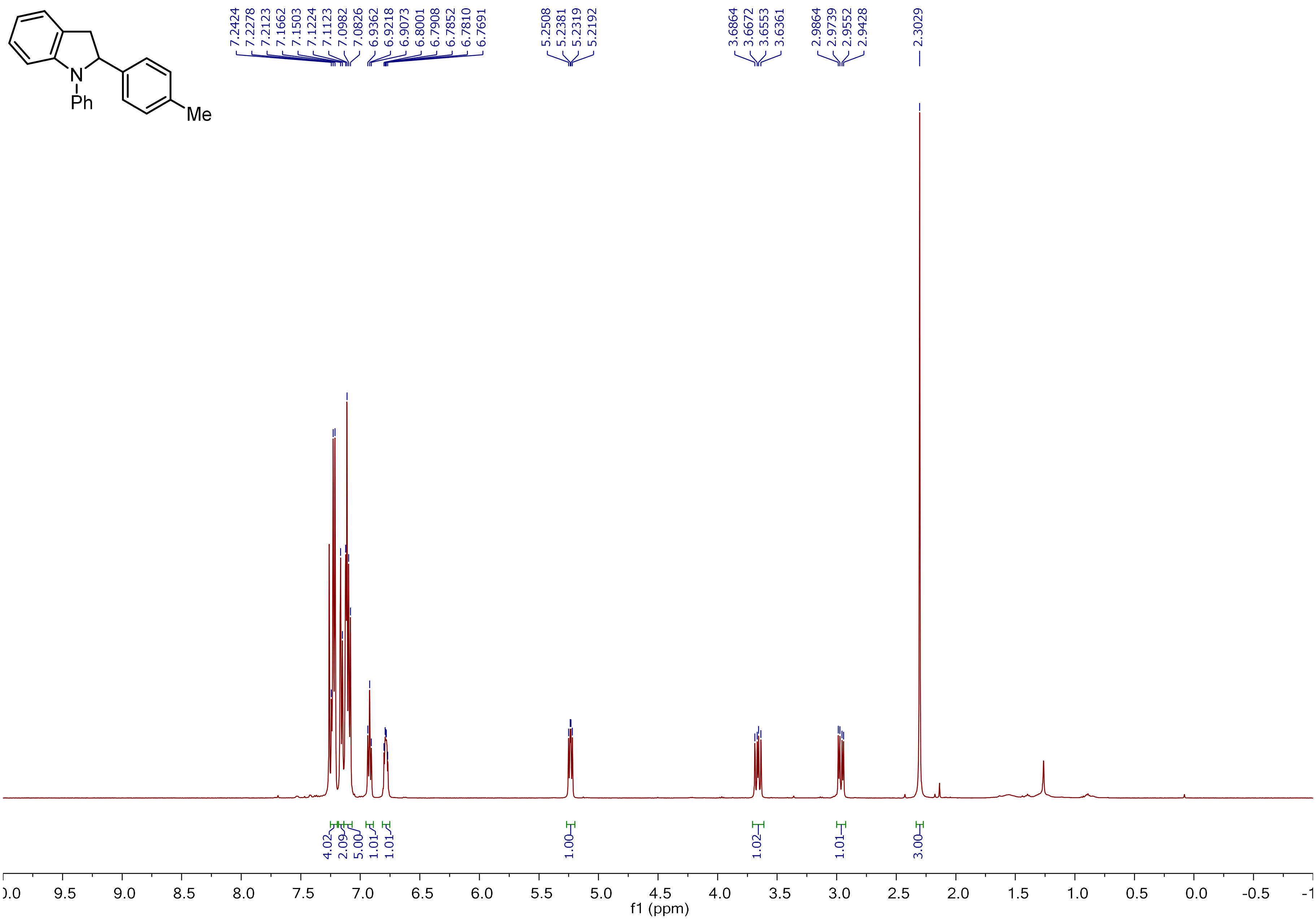

^{13}C NMR (125 MHz, $CDCl_3$): 1-phenyl-2-(*p*-tolyl)indoline (19)

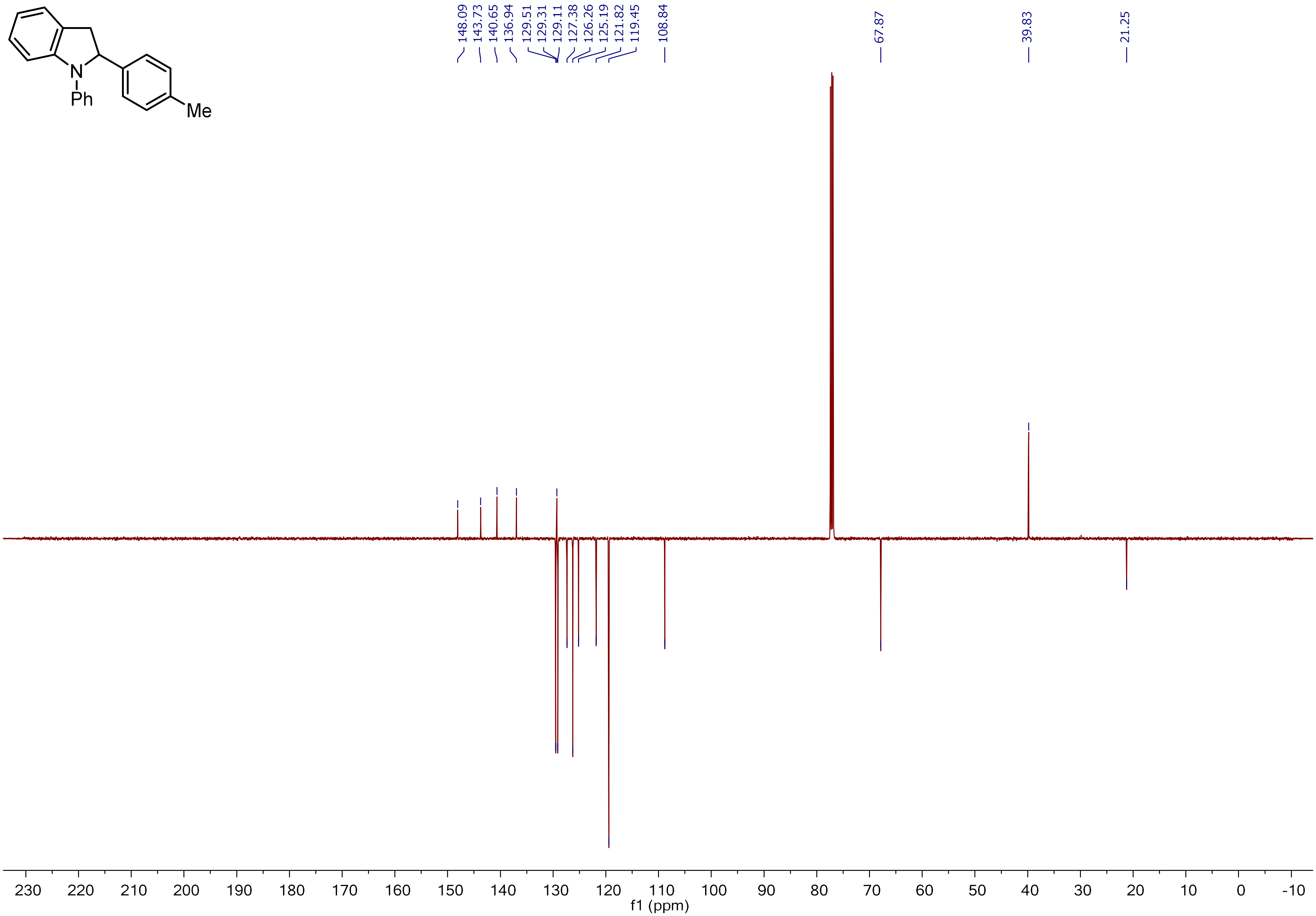

^{1}H NMR (500 MHz, $CDCl_3$): 1-phenyl-2-(*p*-tolyl)-1,2,3,4-tetrahydroquinoline (20)

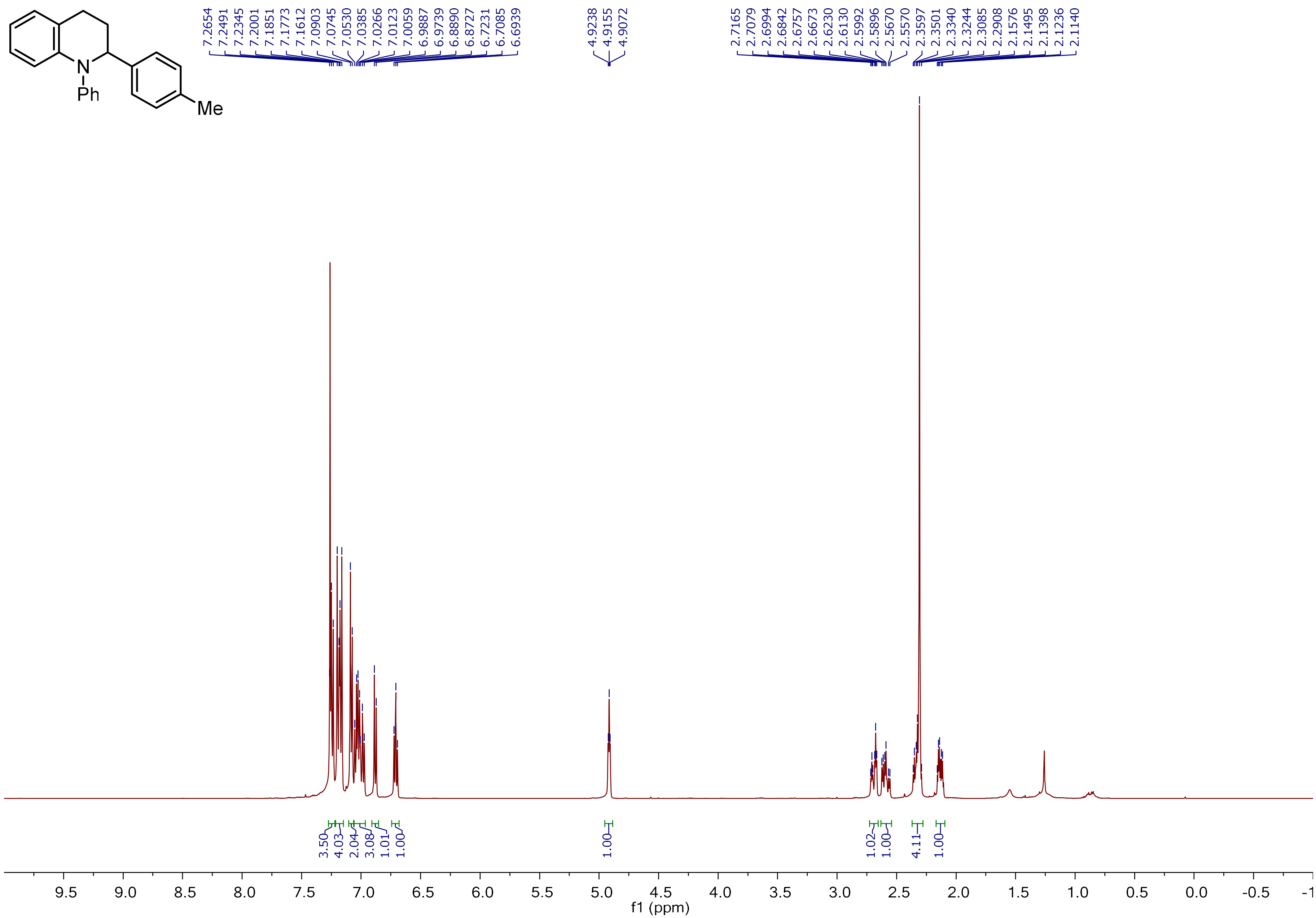

^{13}C NMR (125 MHz, $CDCl_3$): 1-phenyl-2-(*p*-tolyl)-1,2,3,4-tetrahydroquinoline (20)

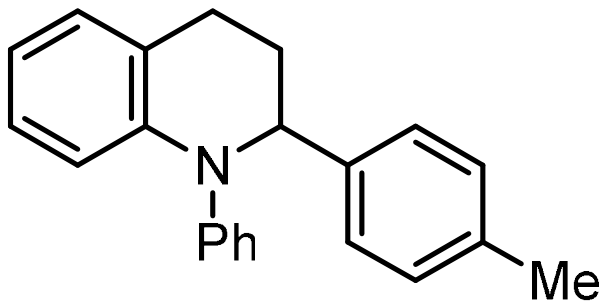

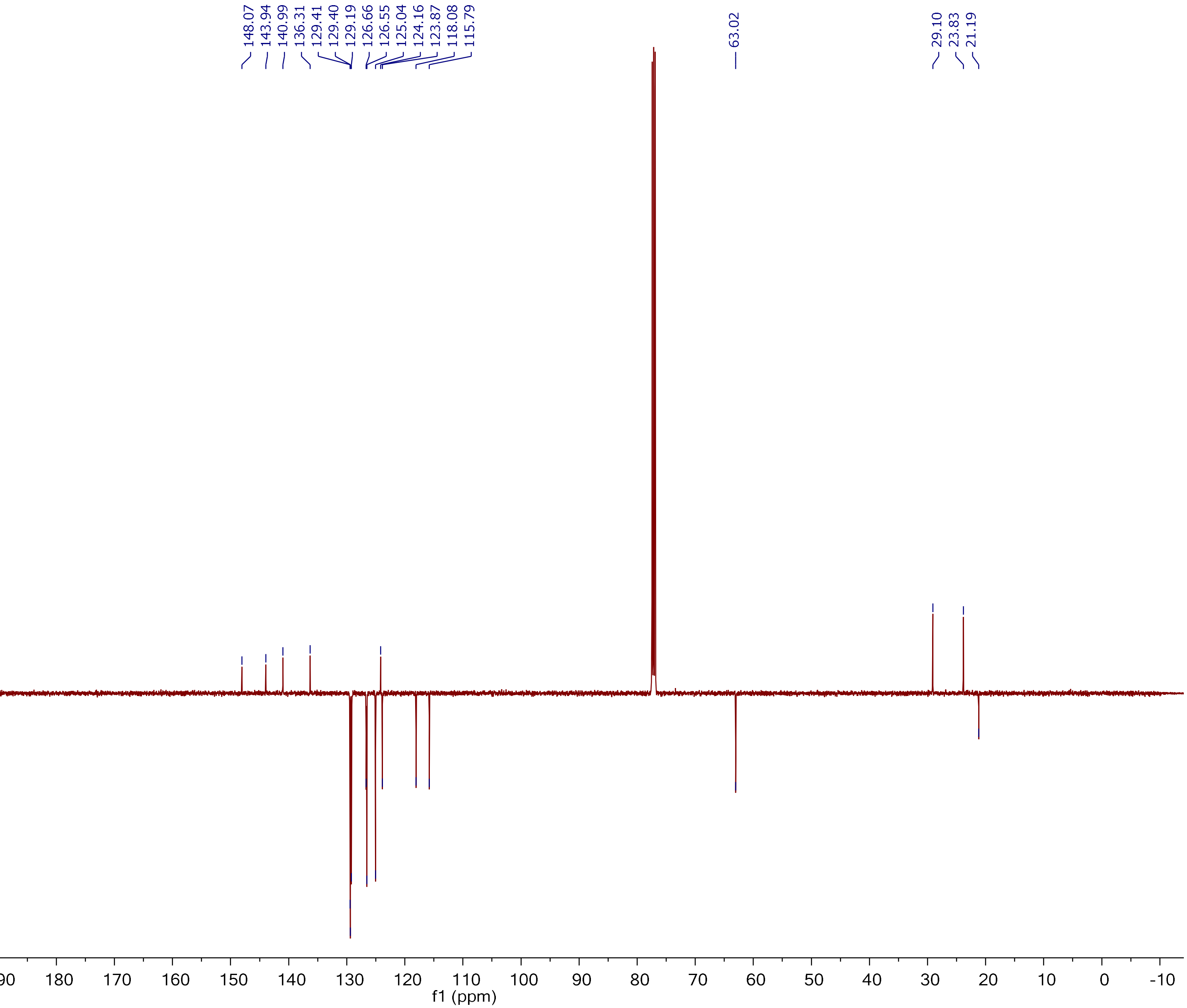

^{1}H NMR (500 MHz, $CDCl_3$): 1-phenyl-2-(*p*-tolyl)piperidine (21)

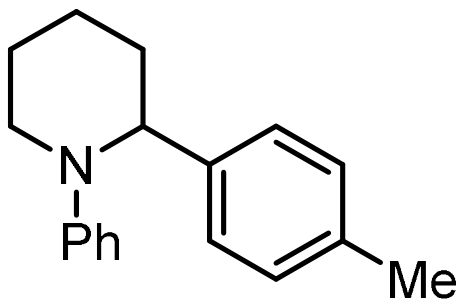

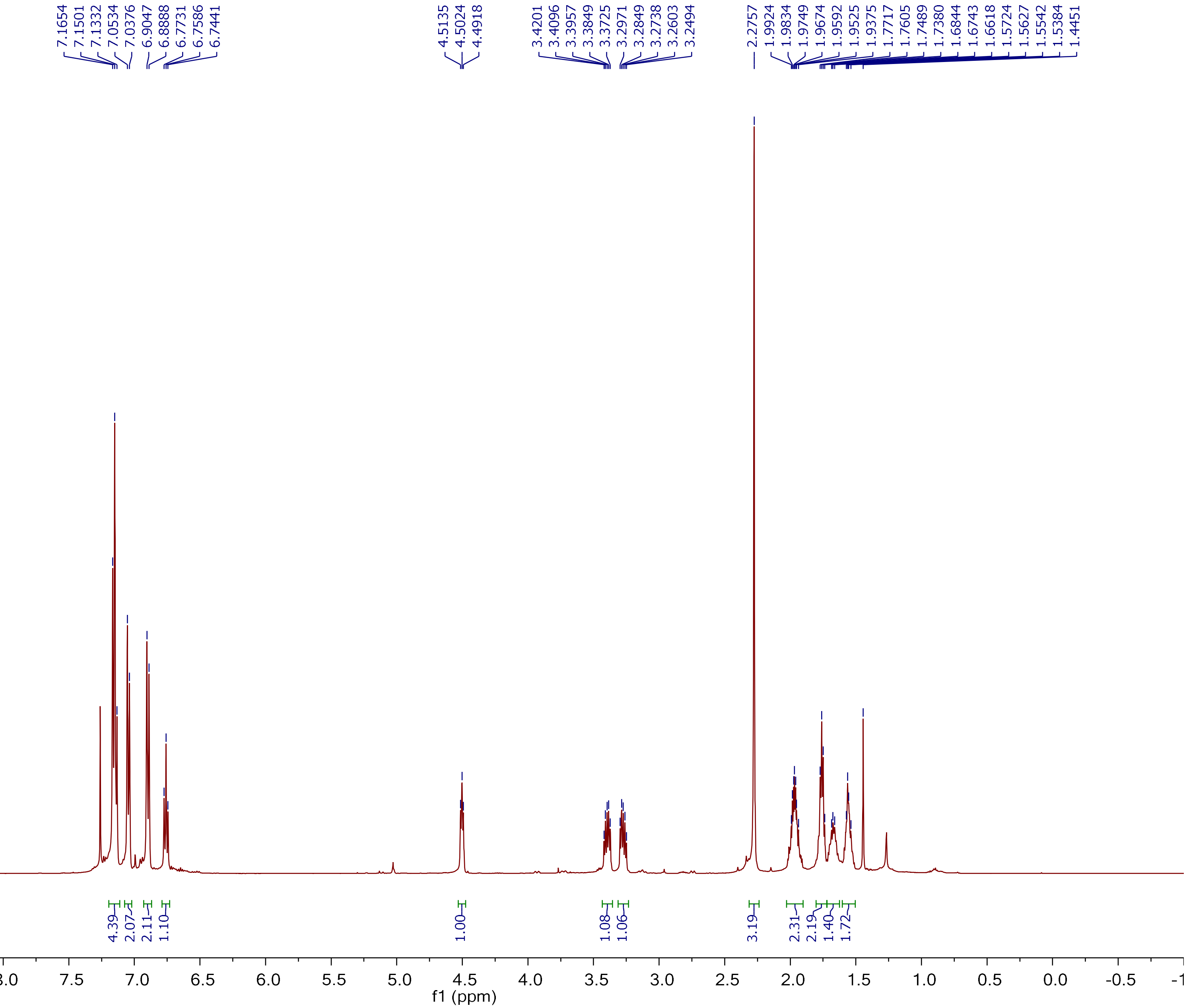

^{13}C NMR (125 MHz, $CDCl_3$): 1-phenyl-2-(*p*-tolyl)piperidine (21)

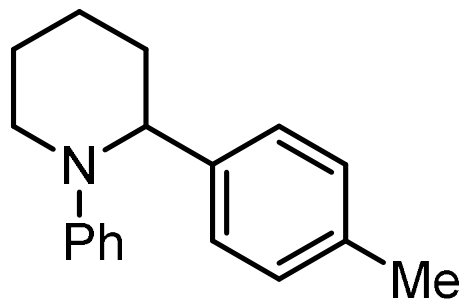

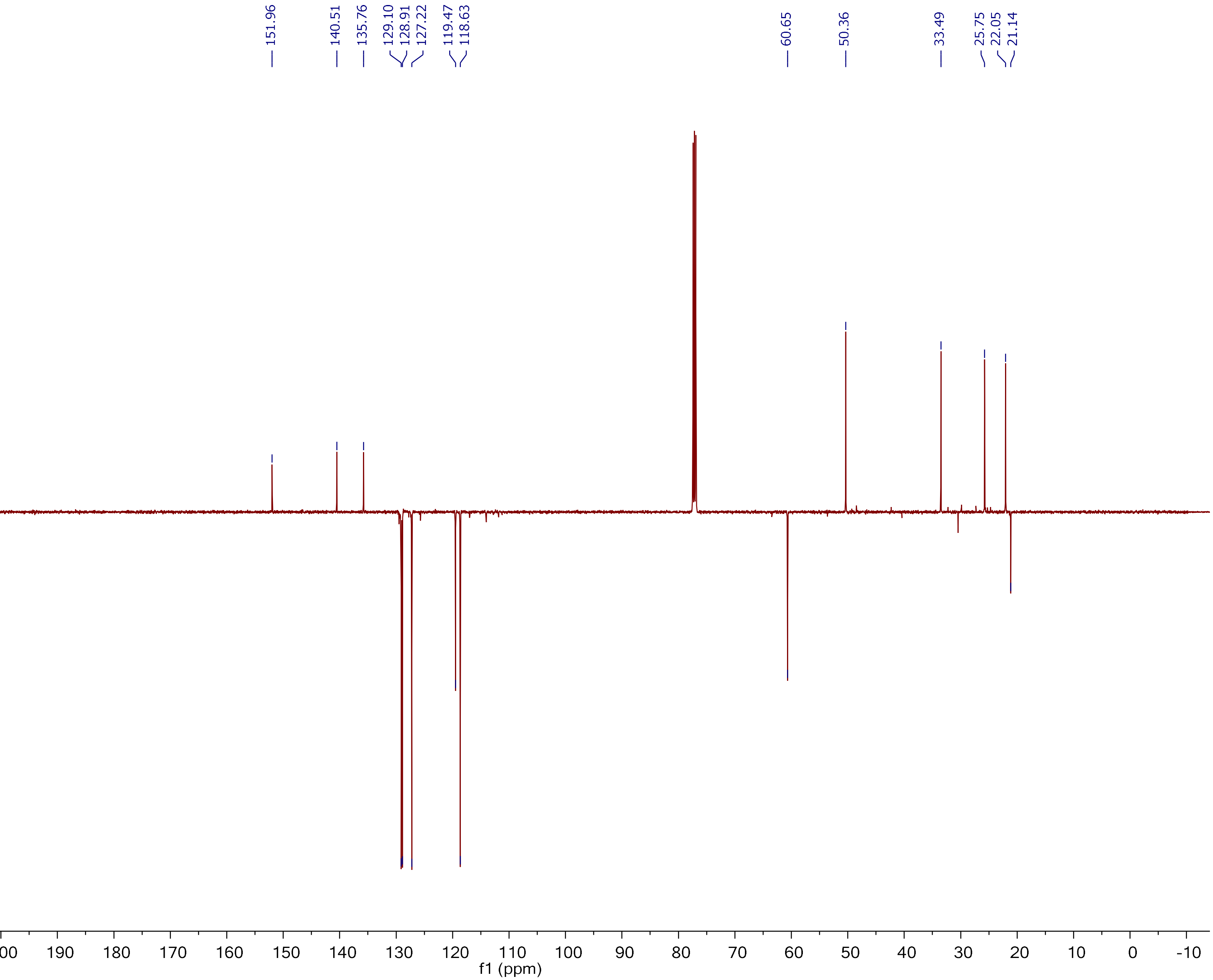

^{1}H NMR (500 MHz, $CDCl_3$): 4-phenyl-3-(*p*-tolyl)morpholine (22)

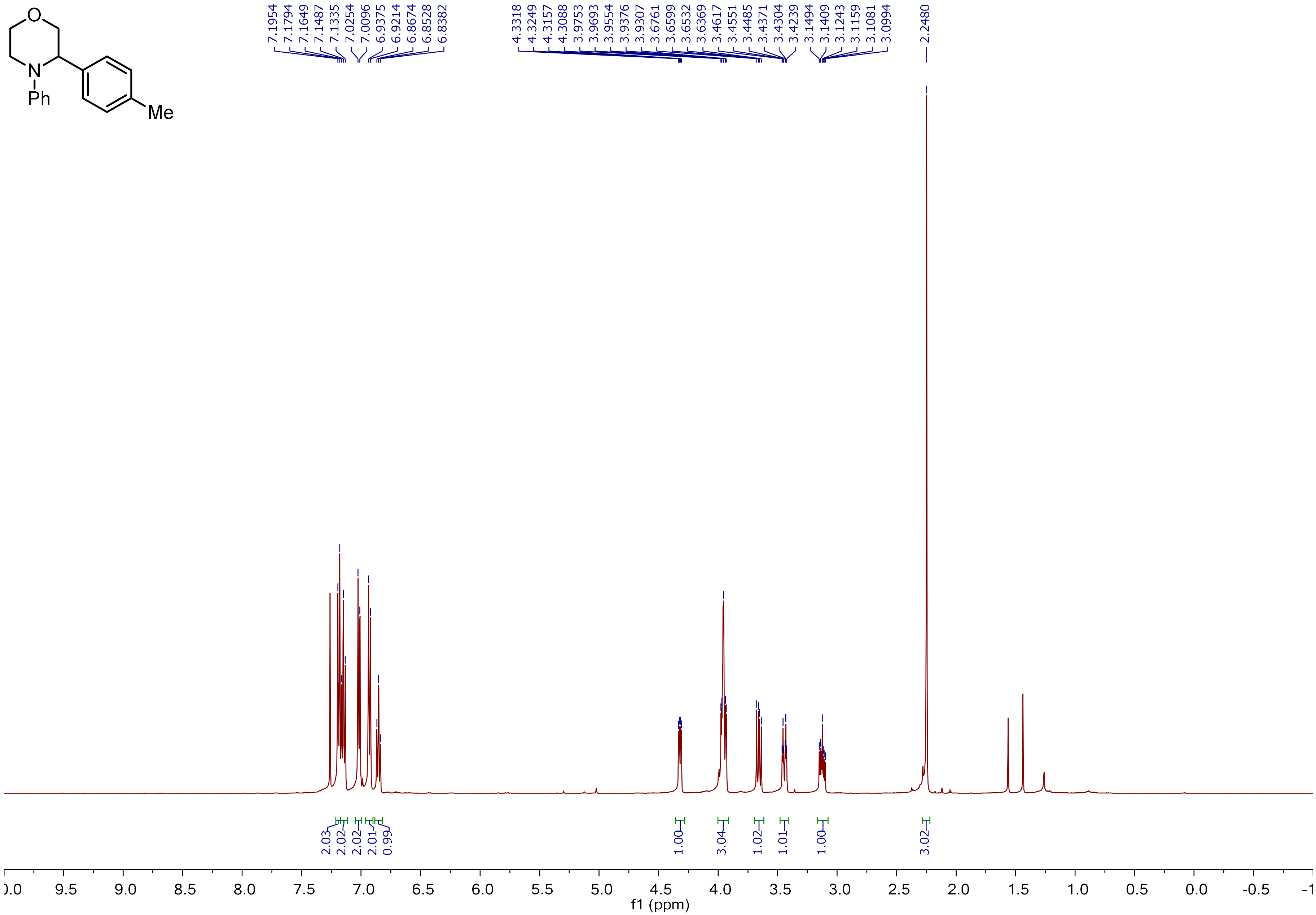

^{13}C NMR (125 MHz, $CDCl_3$): 4-phenyl-3-(*p*-tolyl)morpholine (22)

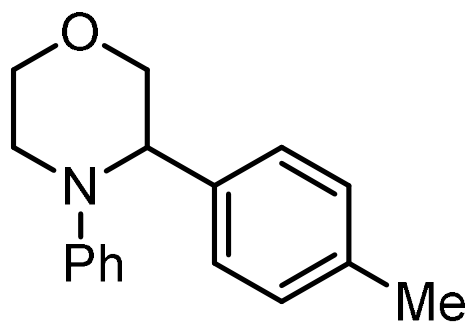

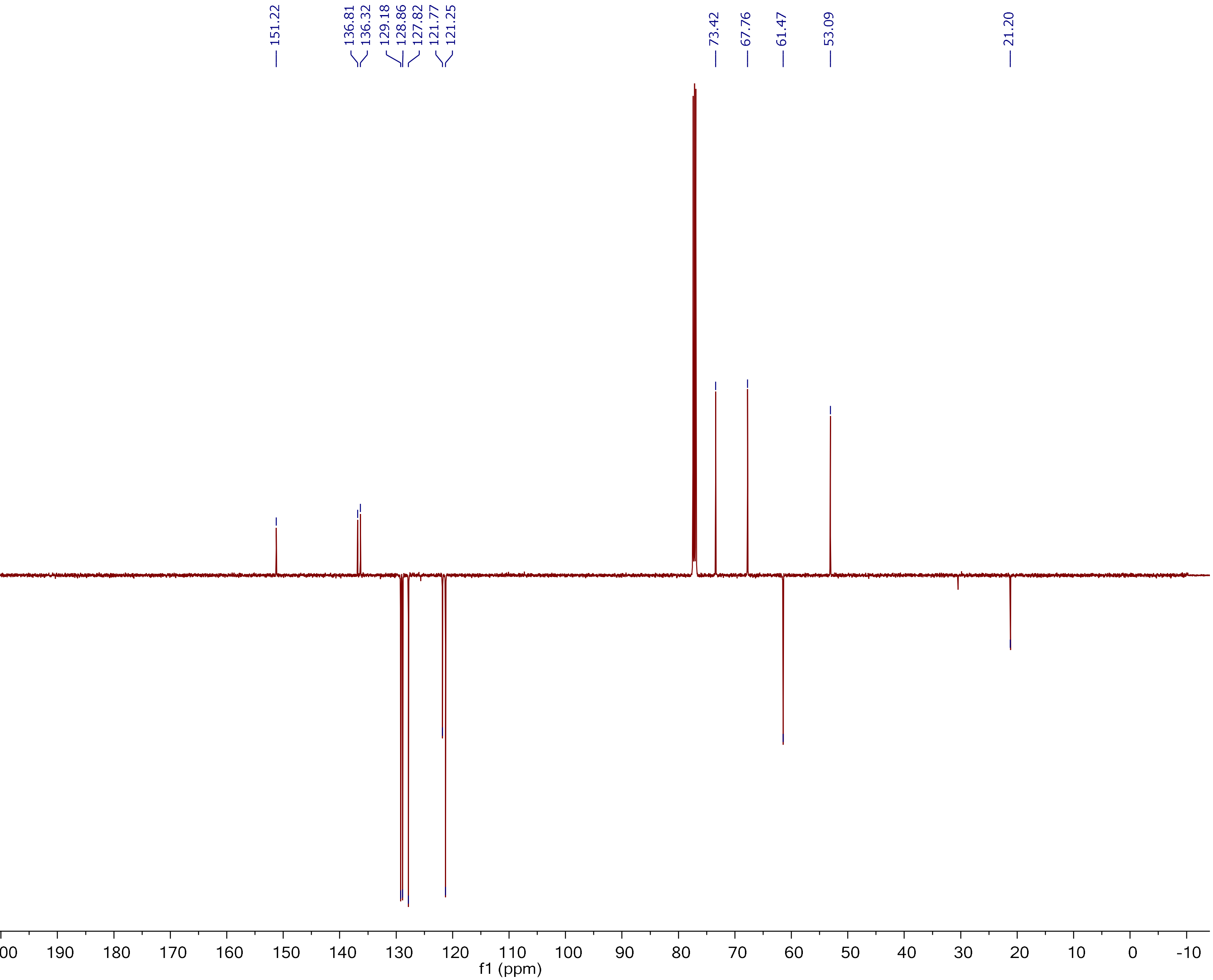

^{1}H NMR (500 MHz, $CDCl_3$): 1-phenyl-2-(*p*-tolyl)azepane (23)

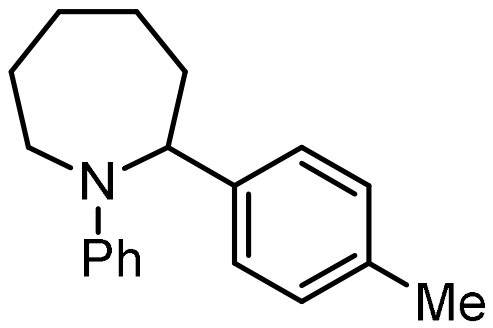

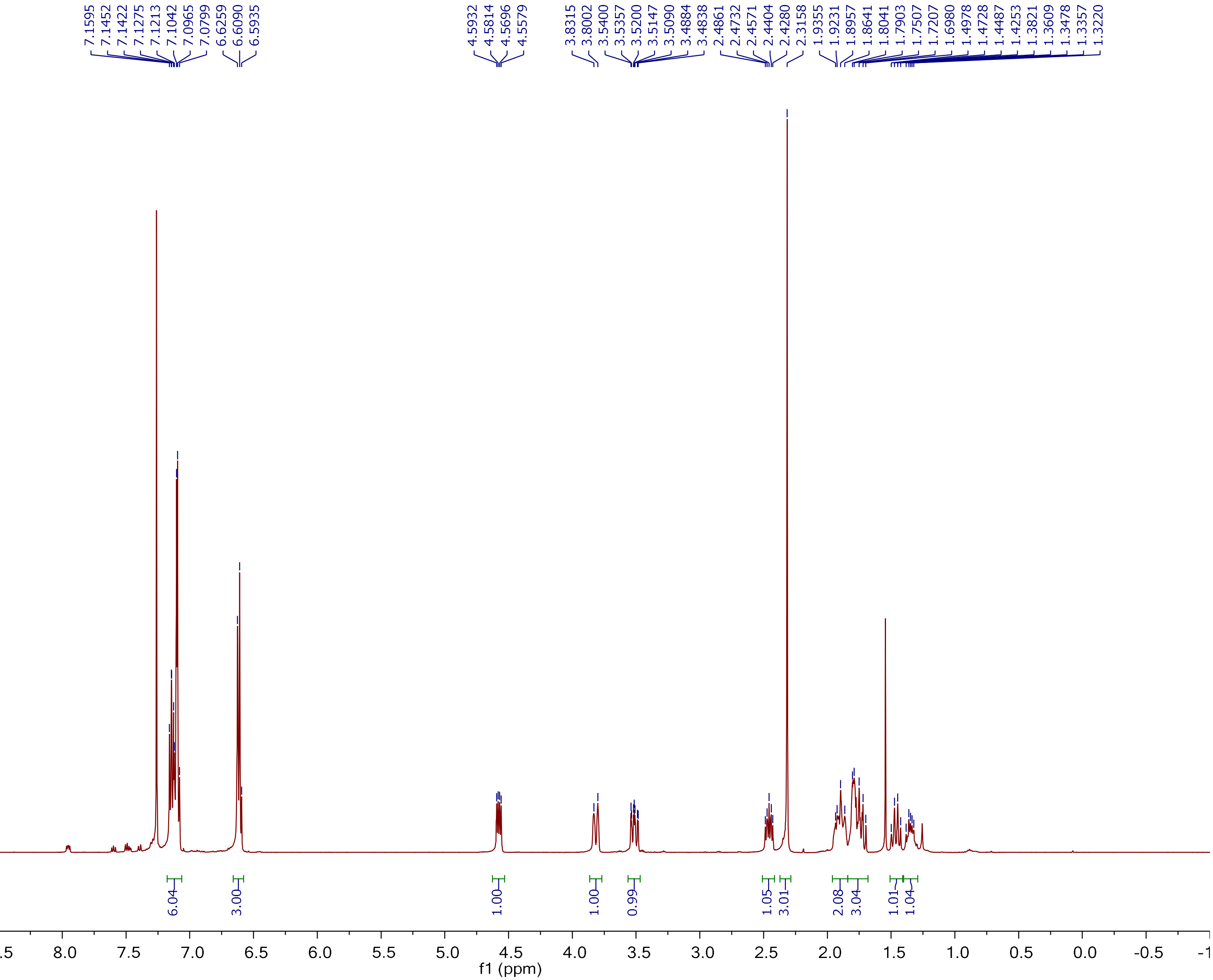

^{13}C NMR (125 MHz, $CDCl_3$): 1-phenyl-2-(*p*-tolyl)azepane (23)

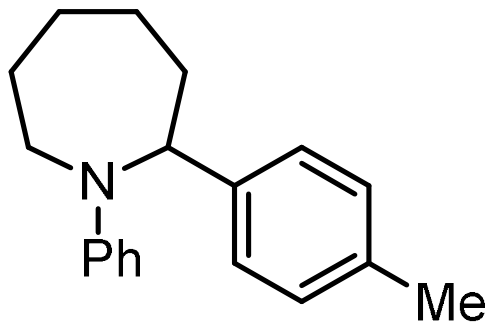

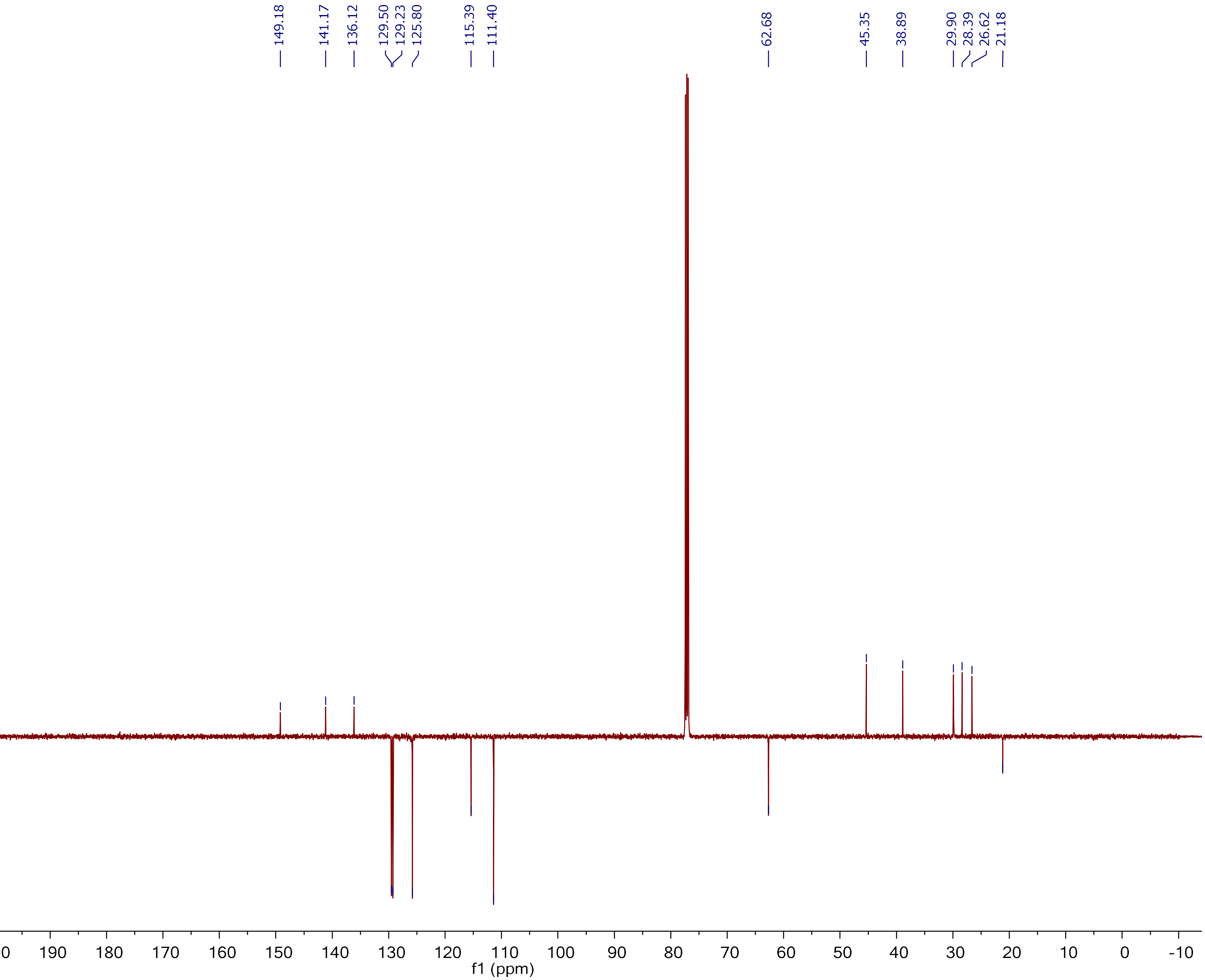

^{1}H NMR (500 MHz, $CDCl_3$): (4*R*,5*R*)-4-((benzyloxy)methyl)-2-phenyl-5-(4-(1-phenylpyrrolidin-2-yl)phenyl)-4,5-dihydrooxazole (24)

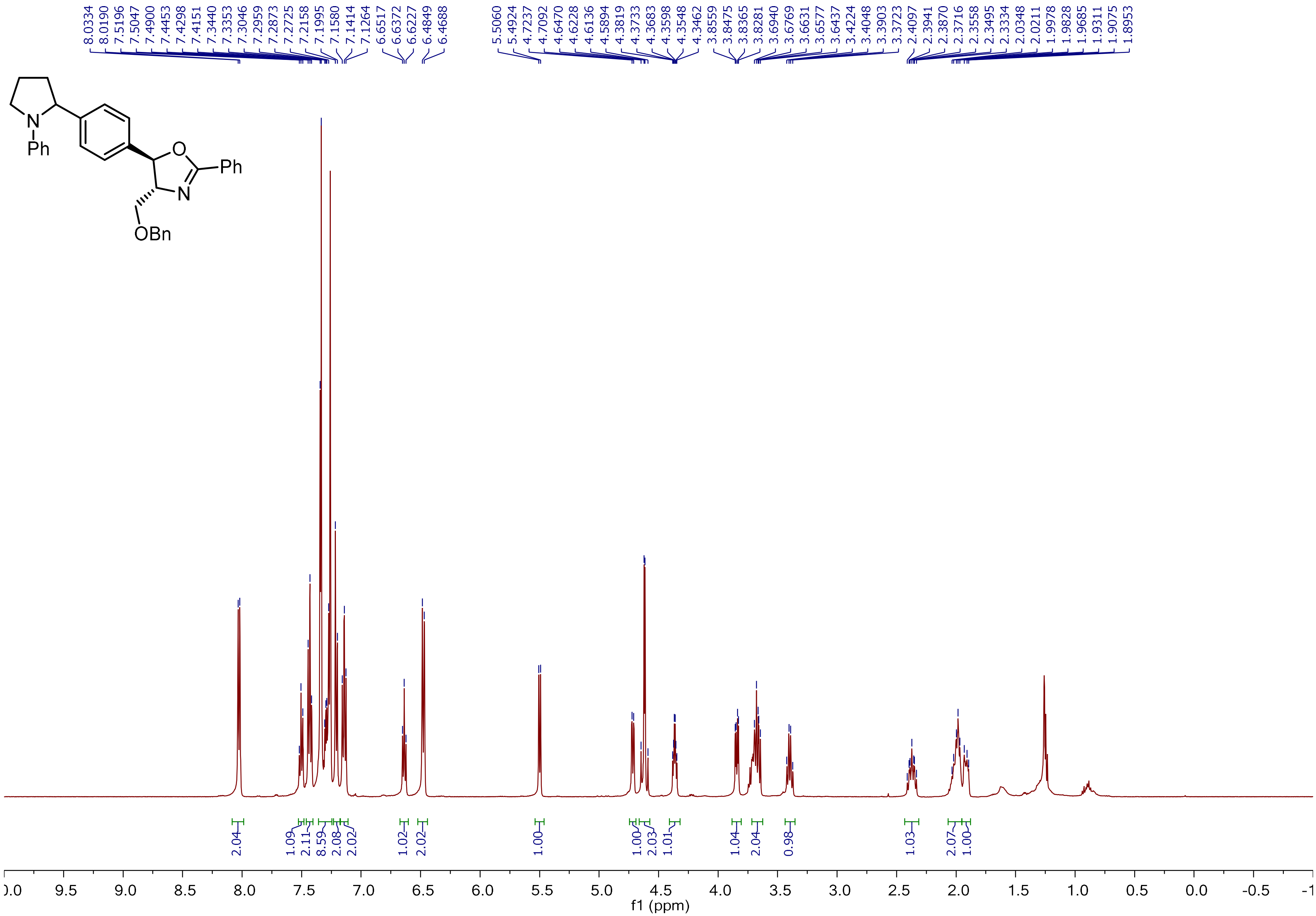

^{13}C NMR (125 MHz, $CDCl_3$): (4*R*,5*R*)-4-((benzyloxy)methyl)-2-phenyl-5-(4-(1-phenylpyrrolidin-2-yl)phenyl)-4,5-dihydrooxazole (24)

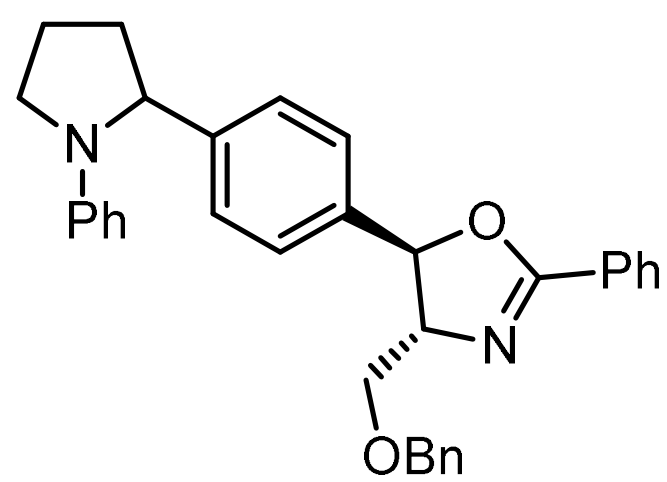

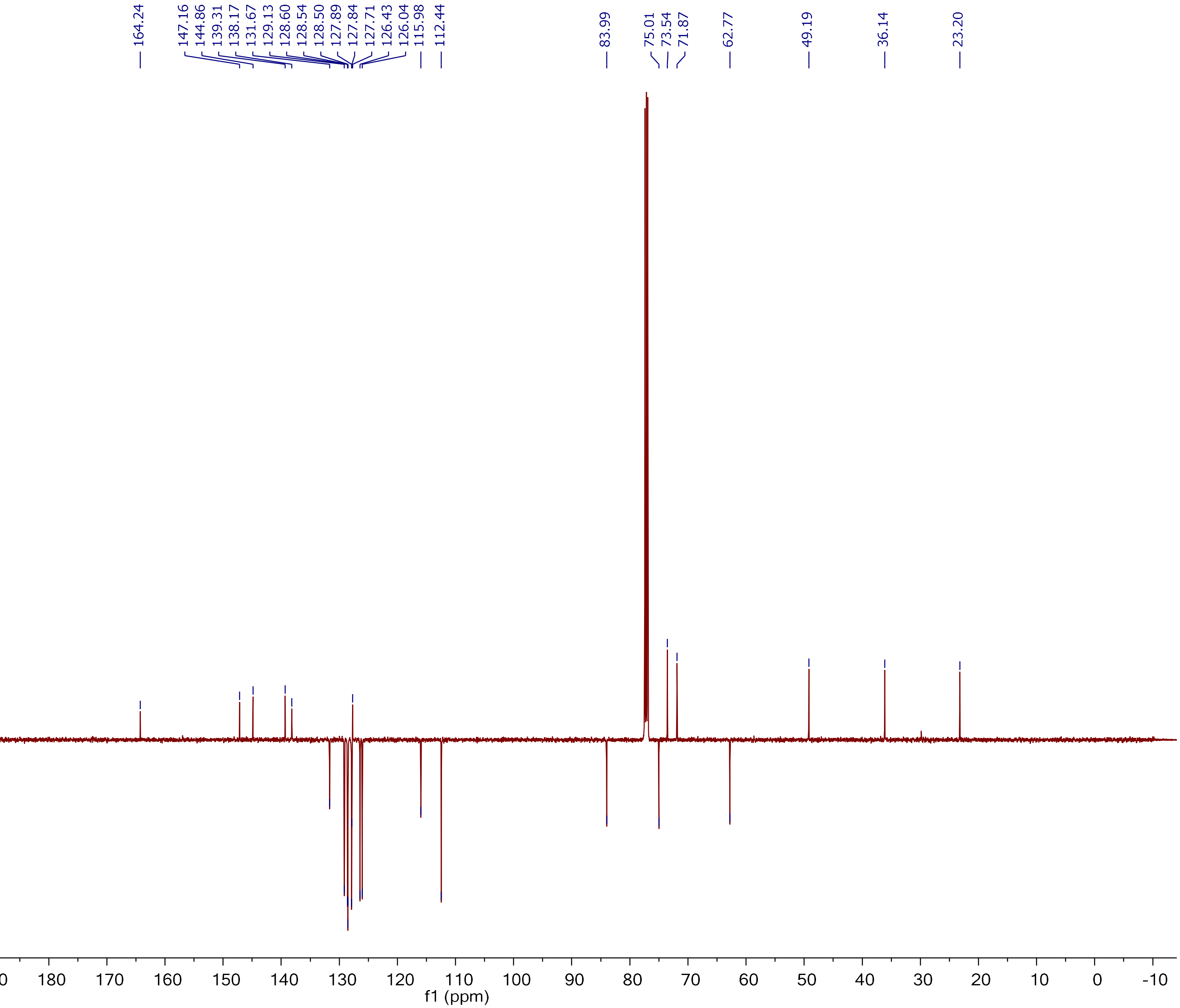

^{1}H NMR (500 MHz, $CDCl_3$): 2,5-dimethyl-1-(4-(1-phenylpyrrolidin-2-yl)phenyl)-1*H*-pyrrole-3-carbaldehyde (25)

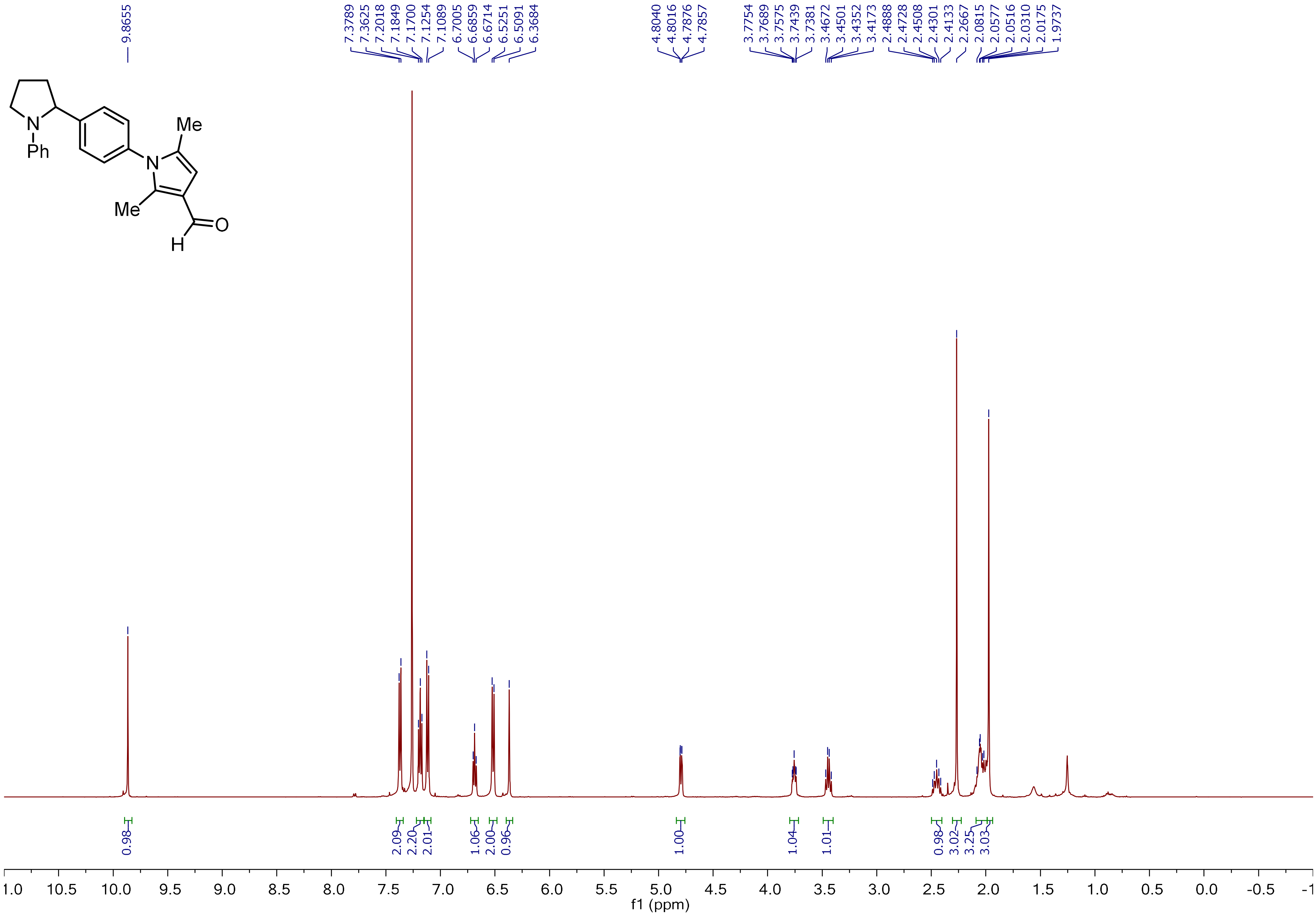

^{13}C NMR (125 MHz, $CDCl_3$): 2,5-dimethyl-1-(4-(1-phenylpyrrolidin-2-yl)phenyl)-1*H*-pyrrole-3-carbaldehyde (25)

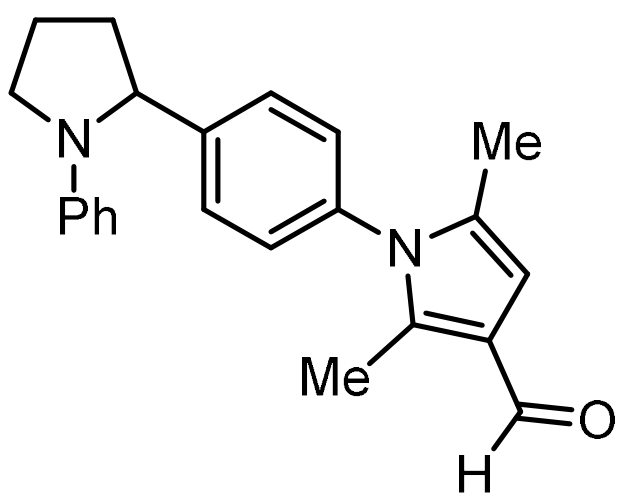

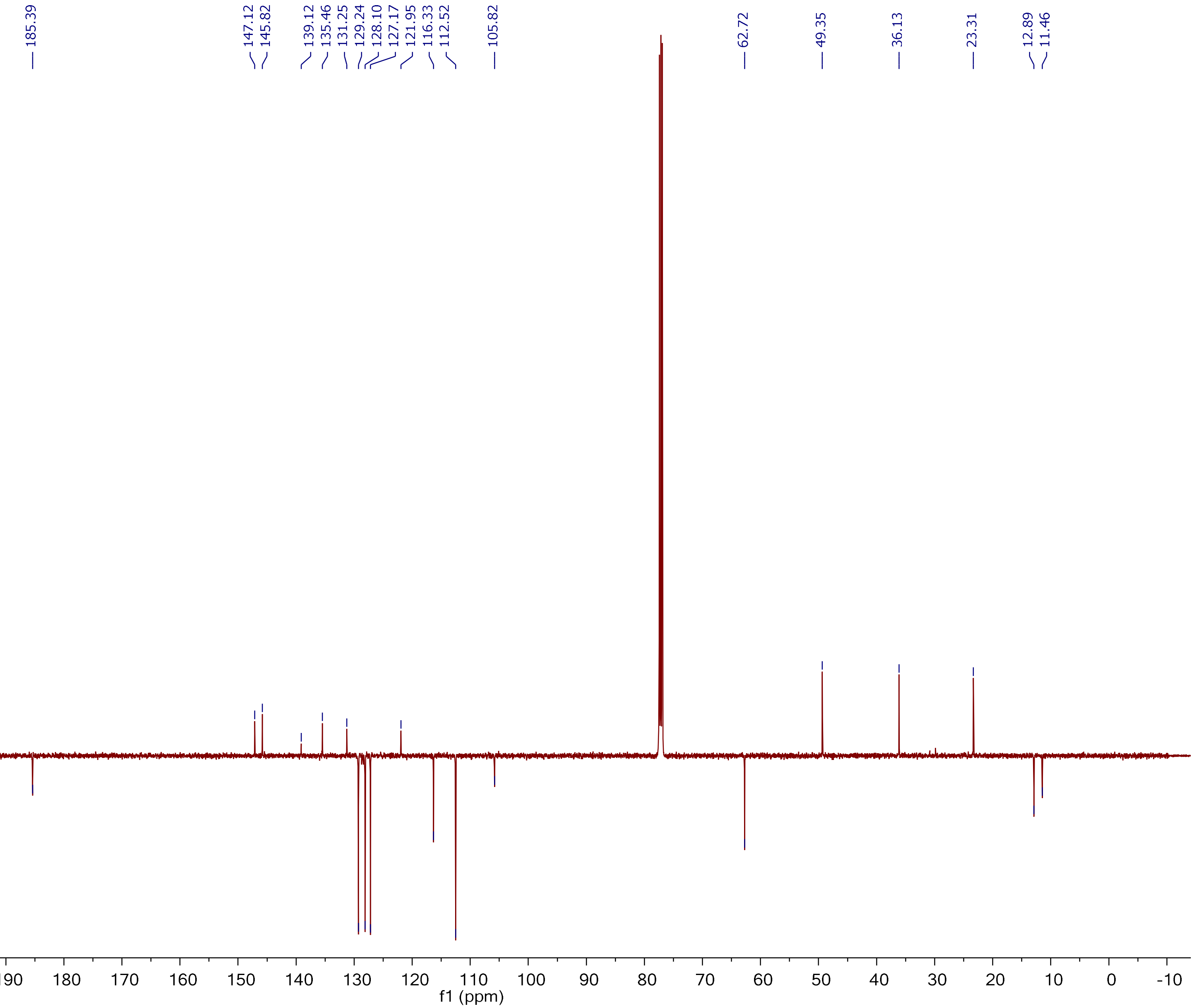

^{1}H NMR (500 MHz, $CDCl_3$): 6-bis(*tert*-butoxycarbonyl)-amino-3-cyclopropyl-1-(2-fluoro-4-(1-phenylpyrrolidin-2-yl)phenyl)pyrimidine-2,4(1*H*,3*H*)-dione (26)

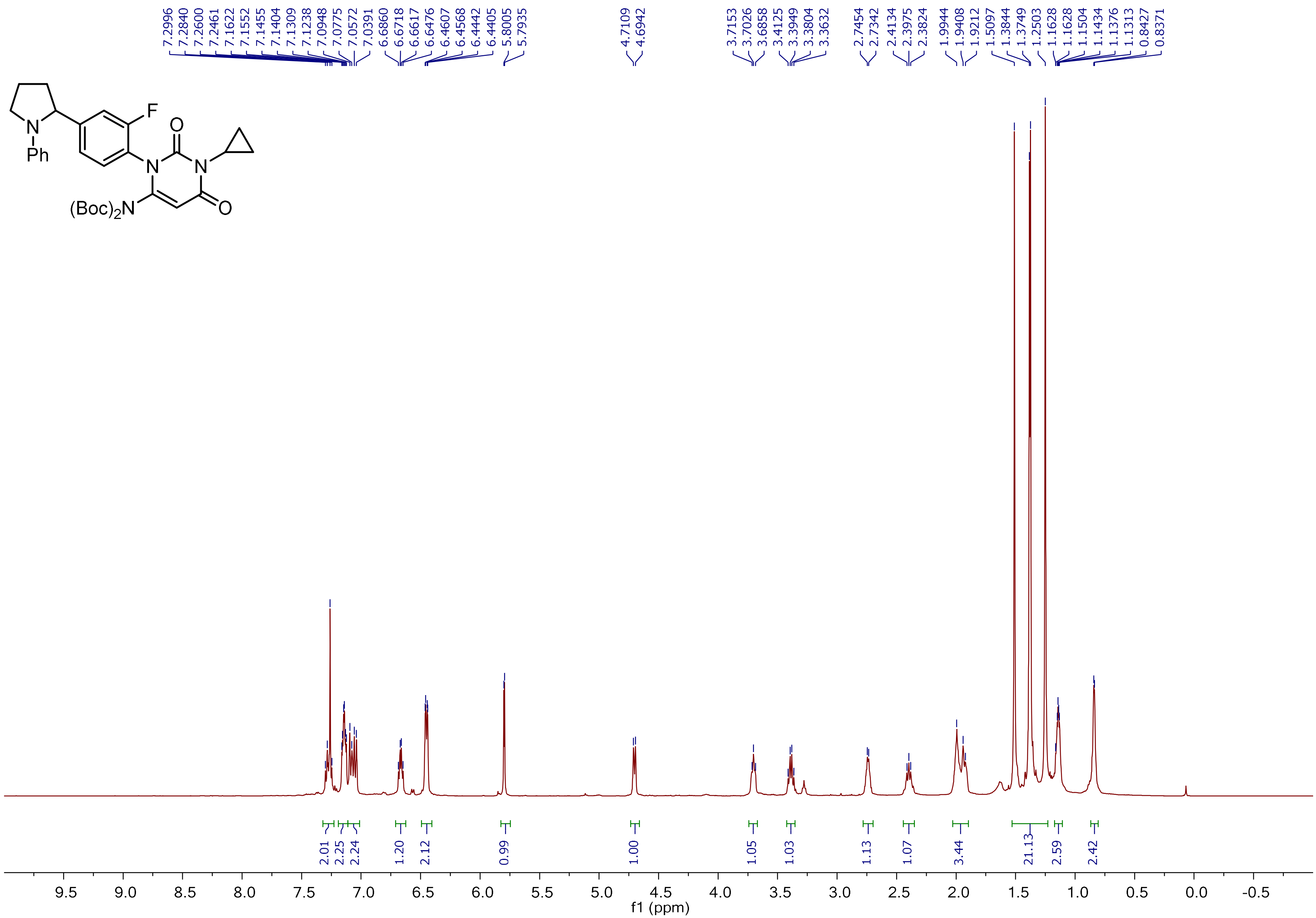

^{13}C NMR (125 MHz, CDCl$_3$): 6-bis(*tert*-butoxycarbonyl)-amino-3-cyclopropyl-1-(2-fluoro-4-(1-phenylpyrrolidin-2-yl)phenyl)pyrimidine-2,4(1*H*,3*H*)-dione (26)

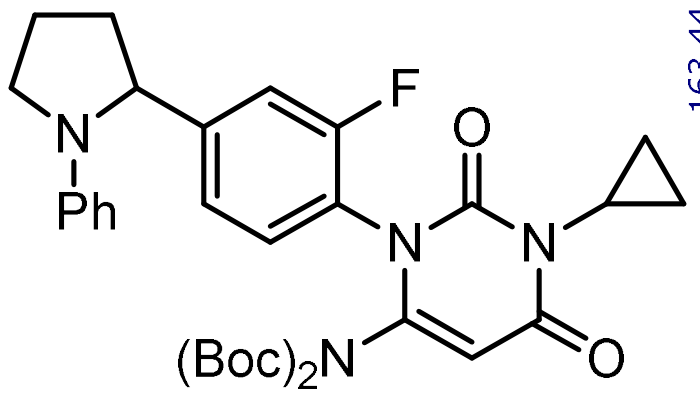

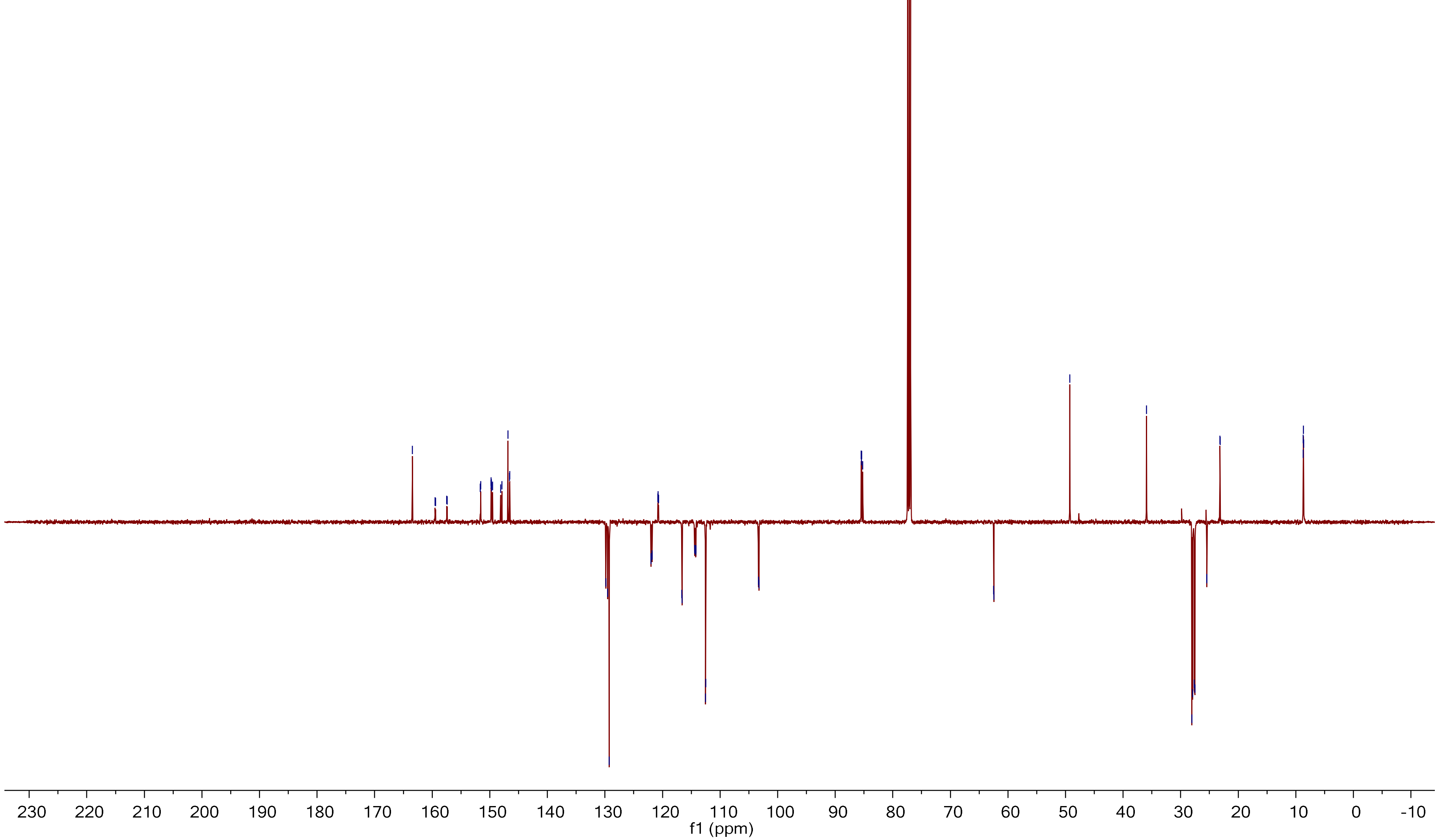

^{19}F NMR (282 MHz, $CDCl_3$): 6-bis(*tert*-butoxycarbonyl)-amino-3-cyclopropyl-1-(2-fluoro-4-(1-phenylpyrrolidin-2-yl)phenyl)pyrimidine-2,4(1*H*,3*H*)-dione (26)

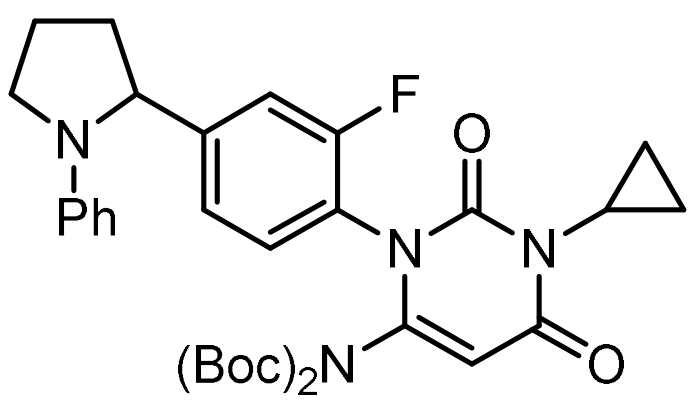

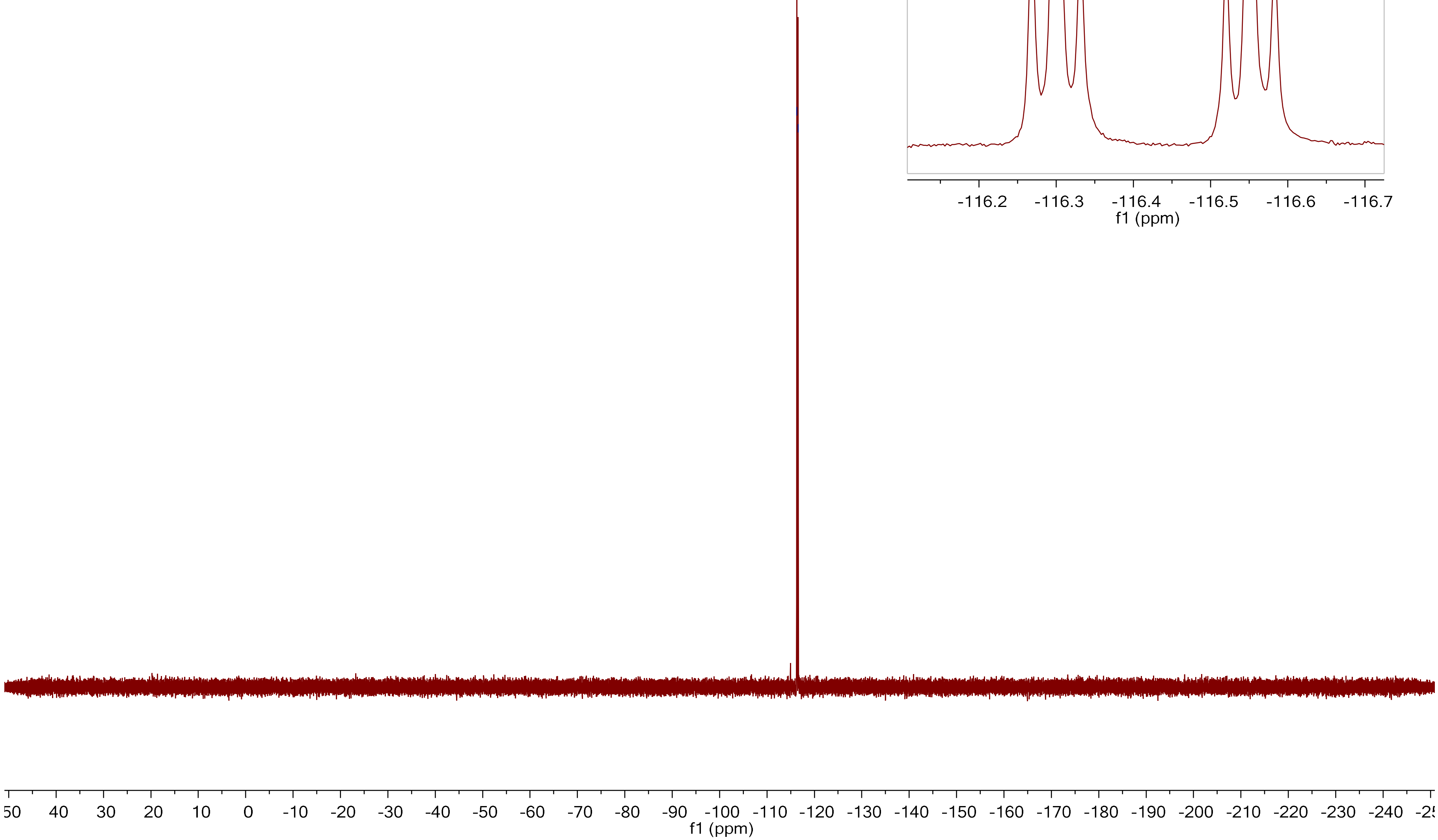

HSQC NMR ($CDCl_3$): 6-bis(*tert*-butoxycarbonyl)-amino-3-cyclopropyl-1-(2-fluoro-4-(1-phenylpyrrolidin-2-yl)phenyl)pyrimidine-2,4(1*H*,3*H*)-dione (26)

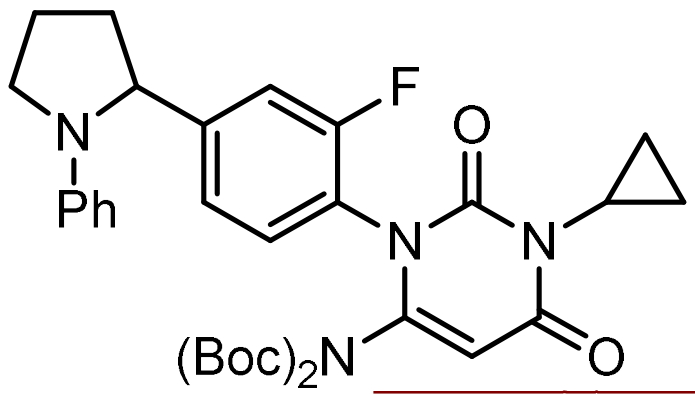

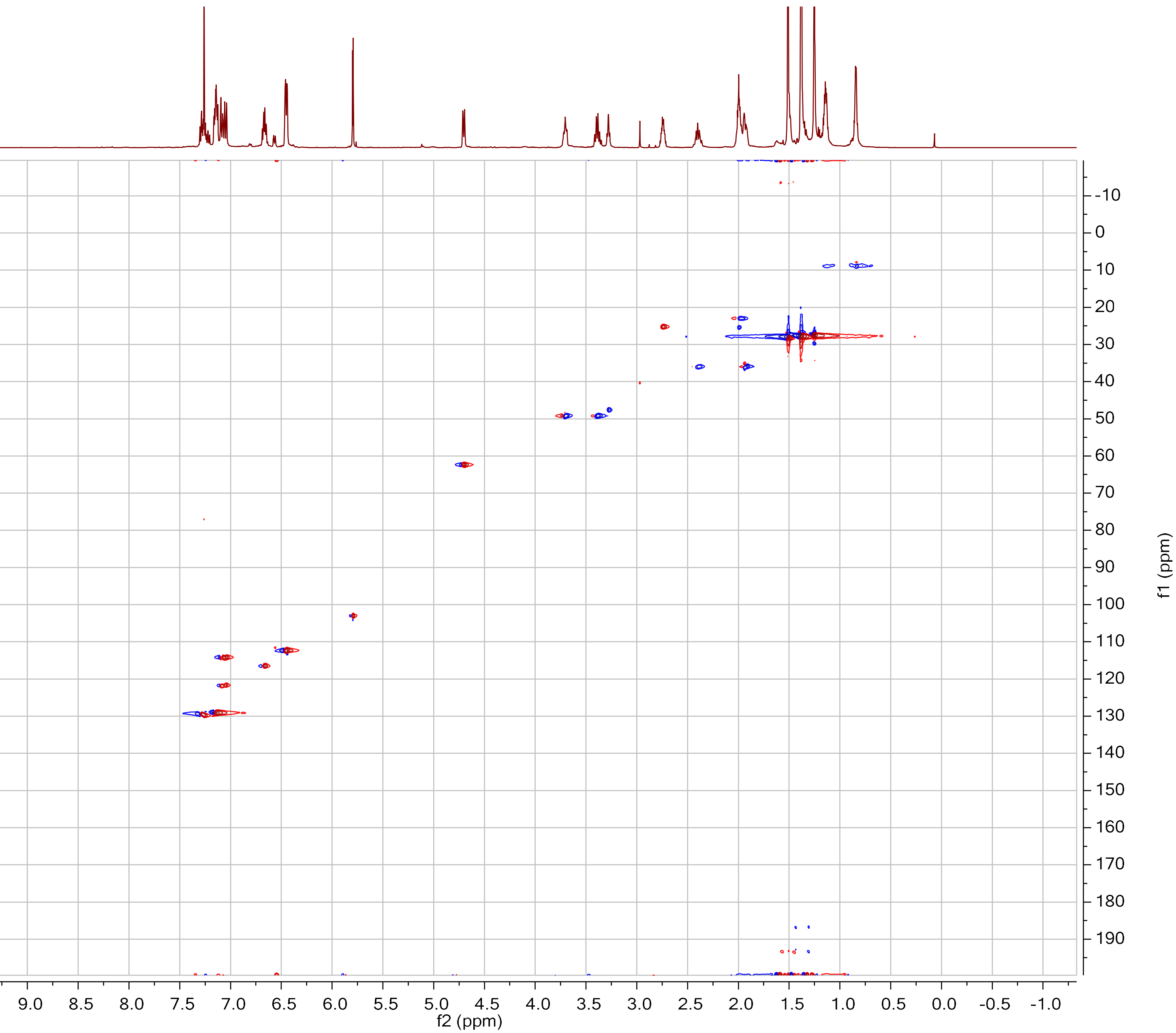

HMBC NMR ($CDCl_3$): 6-bis(*tert*-butoxycarbonyl)-amino-3-cyclopropyl-1-(2-fluoro-4-(1-phenylpyrrolidin-2-yl)phenyl)pyrimidine-2,4(1*H*,3*H*)-dione (26)

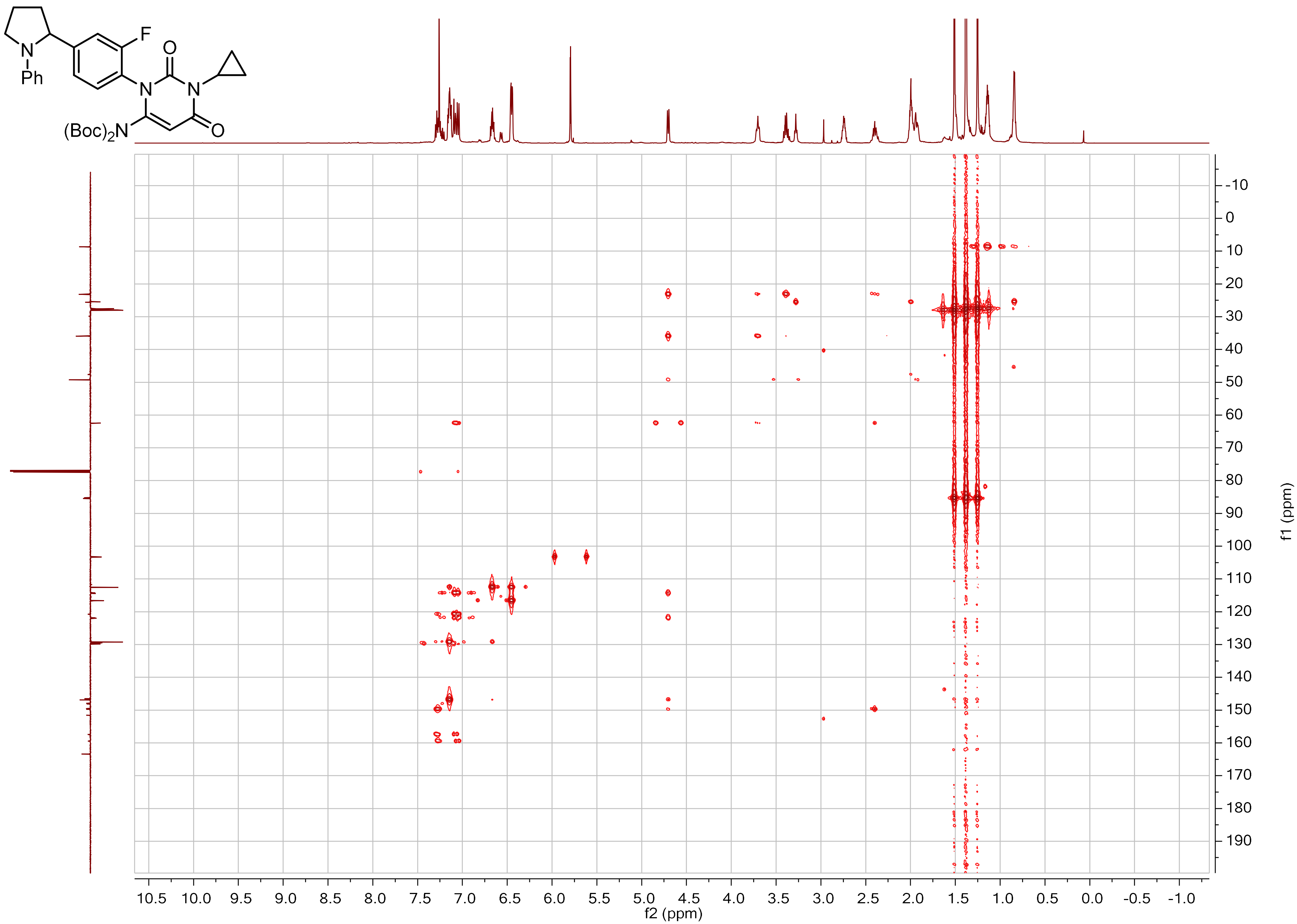

Supplement: Supplementary file 1 [file SC-007-C6SC02815B-s001.pdf]
